# A new chiral phenomenon of orientational chirality, its synthetic control and computational study

**DOI:** 10.3389/fchem.2022.1110240

**Published:** 2023-01-05

**Authors:** Shengzhou Jin, Ting Xu, Yao Tang, Jia-Yin Wang, Yu Wang, Junyi Pan, Sai Zhang, Qingkai Yuan, Anis Ur Rahman, Adelia J. A. Aquino, Hans Lischka, Guigen Li

**Affiliations:** ^1^ School of Chemistry and Chemical Engineering, Nanjing University, Nanjing, China; ^2^ Department of Chemistry and Biochemistry, Texas Tech University, Lubbock, TX, United States; ^3^ Continuous Flow Engineering Laboratory of National Petroleum and Chemical Industry, Changzhou University, Changzhou, Jiangsu, China; ^4^ Department of Mechanical Engineering, Texas Tech University, Lubbock, TX, United States

**Keywords:** orientational chirality, atropisomerism, rotamer, suzuki-miyaura coupling, and sonogashira coupling

## Abstract

A new type of chirality, orientational chirality, consisting of a tetrahedron center and a remotely anchored blocker, has been discovered. The key structural element of this chirality is characterized by multiple orientations directed by a through-space functional group. The multi-step synthesis of orientational chiral targets was conducted by taking advantage of asymmetric nucleophilic addition, Suzuki-Miyaura cross-coupling and Sonogashira coupling. An unprecedented catalytic species showing a five-membered ring consisting of C (sp^2^)-Br-Pd-C (sp^2^) bonds was isolated during performing Suzuki-Miyaura cross-coupling. X-ray diffraction analysis confirmed the species structure and absolute configuration of chiral orientation products. Based on X-ray structures, a model was proposed for the new chirality phenomenon to differentiate the present molecular framework from previous others. DFT computational study presented the relative stability of individual orientatiomers. This discovery would be anticipated to result in a new stereochemistry branch and to have a broad impact on chemical, biomedical, and material sciences in the future.

## 1 Introduction

Chirality and its control have been among the most active topics in science, technology, and public society for over a century (Wang et al., 1983; Dunitz, 2001; Ojima, 2010; Taniguchi et al., 2011; Bao et al., 2020; Bryliakov, 2020; Zhang and Kürti, 2021). Chirality widely exists in functional biomolecules, such as peptides/proteins, DNA/RNA, and carbohydrates, and has been heavily involved in biological mechanisms in human beings, animals, and plants on the Earth (Wagner and Musso, 1983; Pace and Scholtz, 1998; Williams and Perlmutter, 2013). It has been becoming increasingly important since a large number of modern drugs and their building blocks exhibit chirality in their structures and subunits. The chirality of molecular medicine often governs medical treatments in regard to potency and selectivity of drugs in regard to reducing dosages and unwanted side effects (Lung et al., 1995; Hruby et al., 1997; Markwell, 2008; Rouh et al., 2022). In modern materials science, the control of chirality is necessitated so as to achieve challenging optoelectronic properties (Huang et al., 2018; Feng et al., 2019; Li et al., 2020; Zhao et al., 2020; Liu et al., 2021; Oki et al., 2021). It is worth noting that the progress of aforementioned fields should be greatly attributed to achievements made in asymmetric synthesis and catalysis (Dai et al., 2003; Zeng and Chemler, 2007; Cui et al., 2011; Zhou, 2011; Lorion et al., 2017; Song et al., 2017; Liao et al., 2018; Wang and Tan, 2018; Guo et al., 2019; Chen et al., 2020; Ge et al., 2020; Liu et al., 2020; Ma et al., 2020; Zhang et al., 2020; Huang et al., 2021; Wang et al., 2021; Zhao et al., 2021).

**FIGURE 1:**
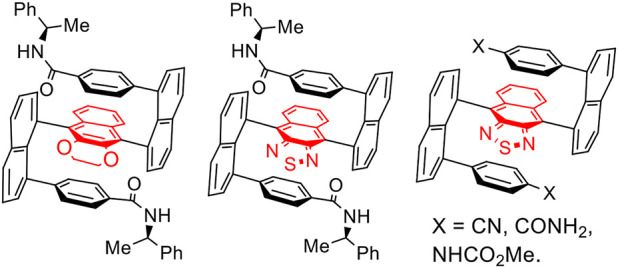
Targets of multilayer folding chirality.

**FIGURE 2:**
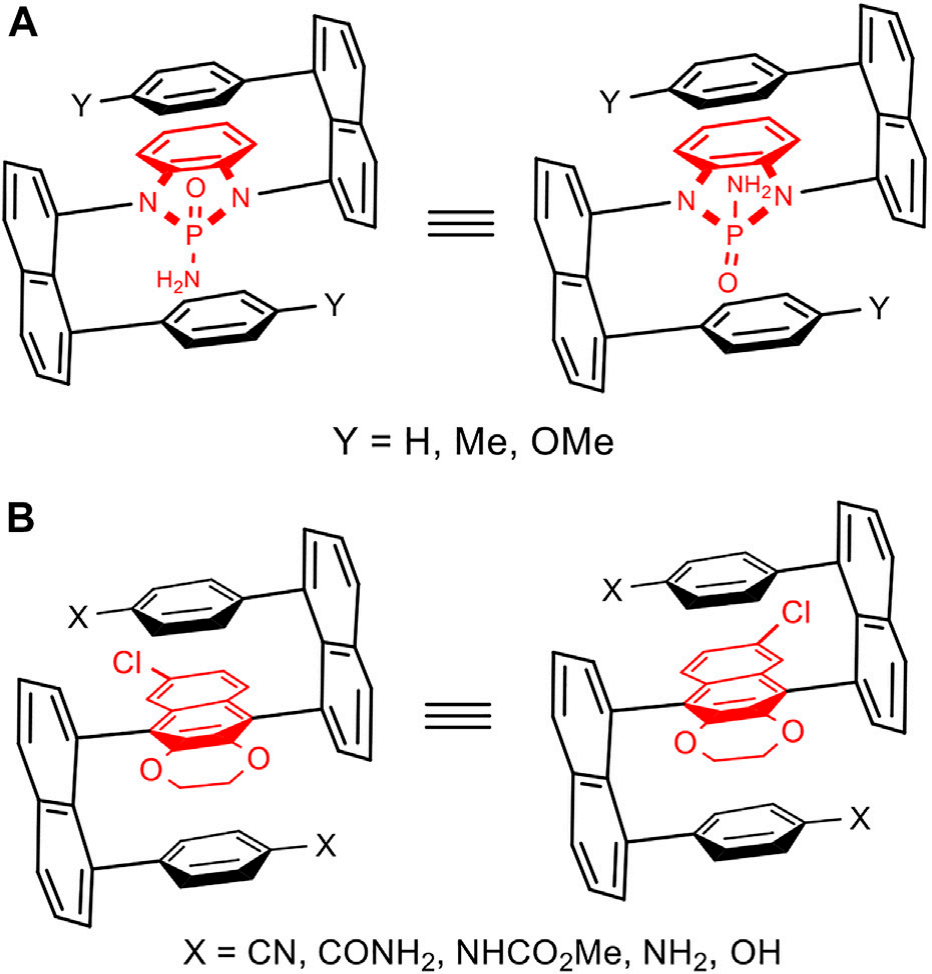
Pseudo C_2_ symmetry in multilayer folding chirality **(A)** and **(B)**.

Chirality is commonly divided into the following categories: central (Zhao et al., 2021), axial (Wang and Tan, 2018; Zhang et al., 2020), spiral (Zhou, 2011), metallic (Dai et al., 2003) and organo (Wu et al., 2019; Liu Y. et al., 2020) sandwich-type, rigid helical (Shen and Chen, 2012; Shirakawa et al., 2016) and flexible folding (Wu, et al., 2019; Wu et al., 2020; Tang et al., 2022; Wang et al., 2022) multilayered chirality (Figure 1). Among these categories, *C*_2_ symmetry has been occupying a special position concerning chirality control, catalyst design and applications (Zhou, 2011; Zhao et al., 2021). Meanwhile, pseudo or quasi *C*_2_ symmetry has also become an interesting and important addition to chirality documents. For instance, organo sandwich chirality shows unique “S” and “Ƨ” (*anti* S) patterns of pseudo *C*_2_ symmetry (Tang et al., 2022b; Jin et al., 2022), and their chiral aggregates displayed various spectroscopic properties (Figure 2).

**FIGURE 3:**
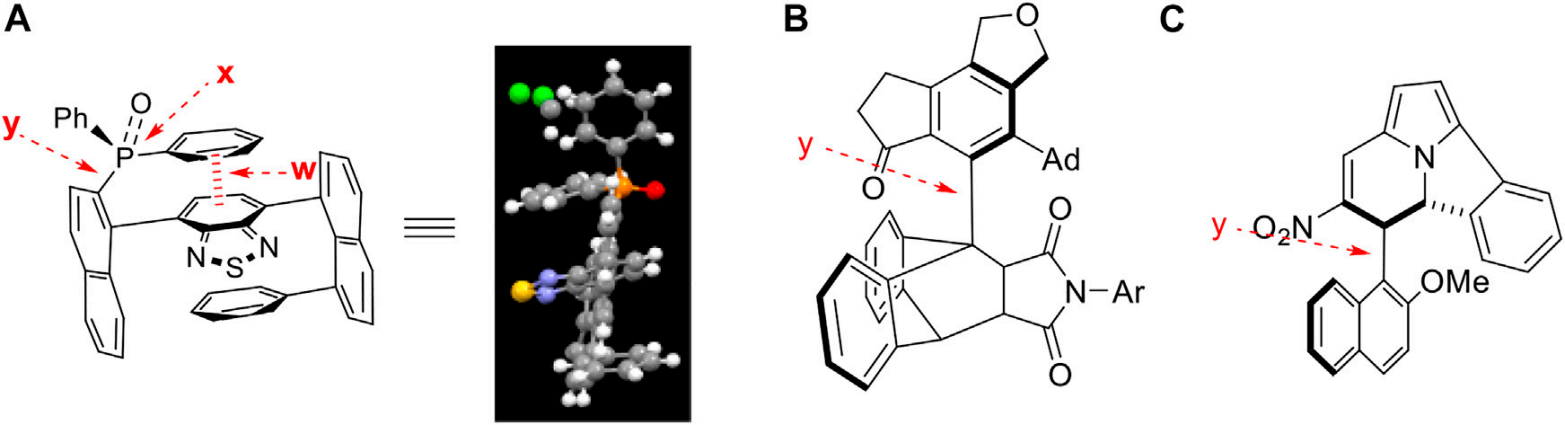
Targets with pseudo chiral center **(A)** and orientational axis **(A–C)**.

Very recently, we reported a new chirality pattern stabilized by aromatic/aromatic interaction (w in Figure 3), as proven by X-ray diffraction analysis (Wu et al., 2021). Interestingly, the new target showed a new chiral subunit containing a pseudo chiral center (x in Figure 3) and orientational axis (y in Figure 3A). The pseudo chiral center on the phosphorus atom was directly connected to naphthalenyl ring and two Ph groups which are differentiated by internally paralleled packing. This pseudo phosphorous center would be extended to other similar centers of tetrahedron (*e.g.,* C and Si) or polyhedron. The atropisomerism along the C-P axis would be made possible not only by Ar-Ar interaction, but also by the special arrangement of the layered phenyl ring on the bottom. Concurrently, Sparr and Jørgensen labs have successfully designed and achieved asymmetric catalytic approaches to stable atropisomers containing C (sp^2^)−C (sp^3^) σ bonds as axes [y in Figures 3B, C] (Wu X. et al., 2021; Bertuzzi et al., 2022). In fact, the tetrahedron-plane based rotamers had not become atropiosmers until when the aforementioned labs were involved in this research (Wu X. et al., 2021; Wu et al., 2021; Bertuzzi et al., 2022), this is due to the fact that rotational barriers around the tetrahedron-plane axis is not large enough.

While continuing the project on multi-layer folding chirality, we found a new pattern of chirality–orientational chirality in which both the chiral center and stereochemical blocker are remotely anchored. The orientational chiral isomers have been stabilized and asymmetrically synthesized through structural analysis and design. Herein, we would like to report our preliminary results of this discovery.

## 2 Discussion and results

**FIGURE 4:**
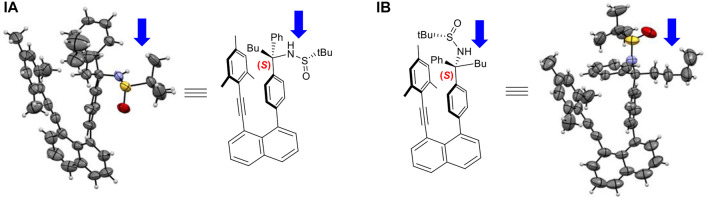
Two differentiated orientantional isomers of **(IA)** and **(IB)**.

This project was initiated by our vision that chirally layered structures would lead to asymmetric control of synthetic reactions by taking advantage of through-space asymmetric induction. For instance, using a chirally attached para-carbon center on the right phenyl ring would generate stereospecific control for reactions on mesitylene subunit nearby. 8-(Mesitylethynyl)naphthalen-1-yl)phenyl-derived sulfonamide was thus generated in the formation of crystalline solids. We pleasingly found that two different rotamers co-exist in their crystals, as revealed by X-ray diffraction analysis (Figure 4). This observation would indicate the possibility of achieving individual atropisomers centered on sp^3^ carbon. It is worth noting that this atropisomerism is based on four independent flexible motifs on sp^3^ carbon, which made the present atropisomerism to be differentiated from previous systems containing cyclized rigid substituents centered on sp^3^ carbon (Wu X. et al., 2021; Bertuzzi et al., 2022).

**FIGURE 5:**
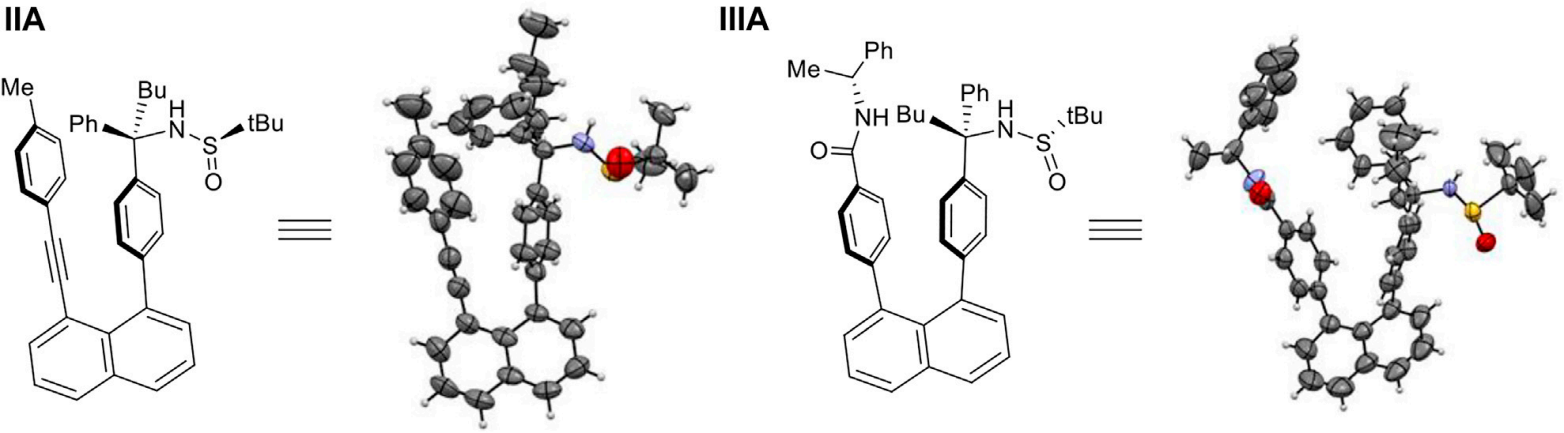
Stabilized individual orientantional isomers **(IIA)** and **(IIIA)**.

**FIGURE 6:**
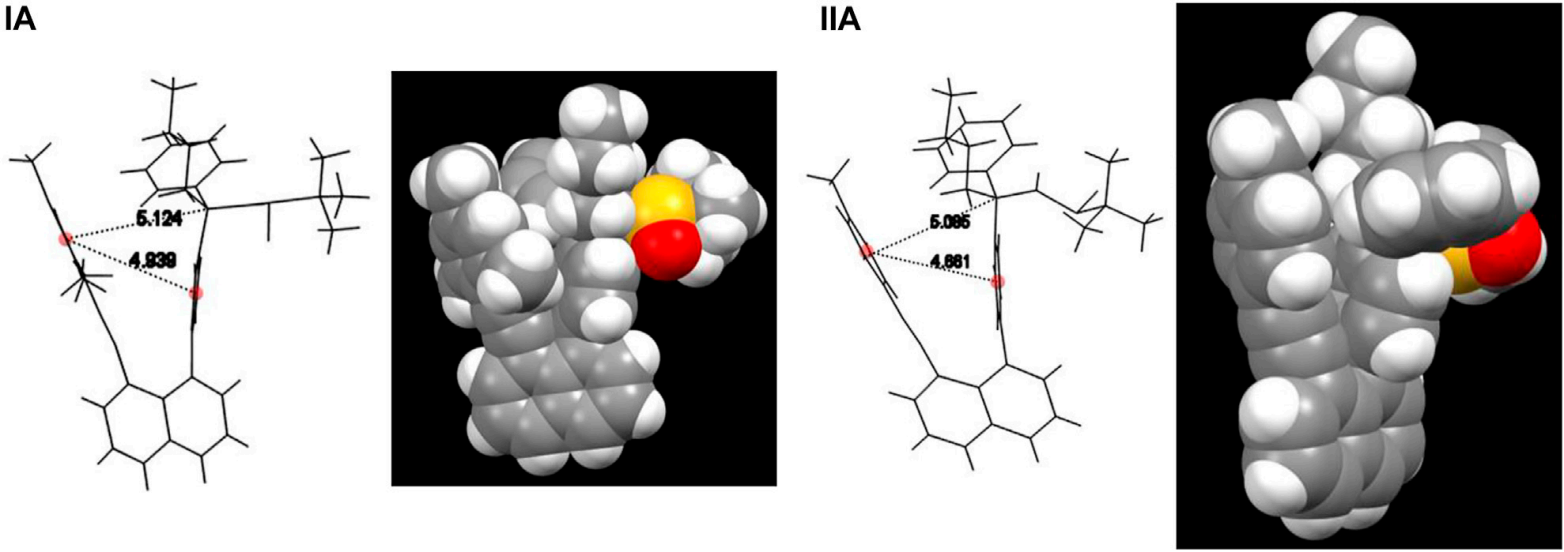
Models and distances of orientational isomers **(IA)** and **(IIA)**.

This encouraging observation inspired us to conduct the structural analysis, design and synthesize derivatives of (**IA**) or (**IB**) to increase energy barriers to prevent individual isomers from rotating. To our pleasant, the stable orientatiomers were successfully achieved through structural design, as shown in Figure 5. The first crystals of **IA** showed the co-existence of two rotamers in a ratio of 1:1, in which n-butyl- and sulfinyl-amino groups are directed away from the shielding mestyl plane, respectively. This indicates that low energy barriers led to freely rotating along sp^2^-sp^3^ σ bond in solution prior to forming crystals. Rotational barriers heavily depend on the distance between two anchored levers/arms on 1,8-positions of naphthalene which is opened wider gradually toward the two ends on top of the structural framework. While the *p*-methyl group on the mesityl ring plays a crucial role in controlling rotation along sp^2^-sp^3^ σ bond, its 2,5-methyl groups widen the distance between two levers/arms (Figure 6); this is attributed to sp^3^ hybridization of methyl groups appearing as two round balls connected on two sides of aromatic ring symmetrically. For this reason, we utilized the *p*-methylphenyl group to replace its mesityl counterpart to avoid the steric effects by two symmetric round balls on the left arms/levers. (*R*)-2-Methyl-*N*-((*R*)-1-phenyl-1-(4-(8-(p-tolylethynyl)naphthalen-1-yl)phenyl)pentyl)propane-2-sulfinamide (**IIA**) was thus designed and synthesized to give crystalline solids. X-ray diffraction analysis of single crystals has proven the aforementioned hypothesis, *i.e*., removing the two methyl groups on mesityl ring did shorten the distance of Ph-to-Ph (phenyl rings’ center-to-center) from 4.939 Å to 4.661 Å, and sp^3^ chiral center to the left phenyl ring center from 5.124 Å to 5.095 Å, respectively (Figure 6). The shortened distance enabled the steric effect by tolyl blocker to be large enough leading to the formation of an atropisomer (**IIA**). In this atropisomer, the sulfinyl amino group is directed away from the *p*-tolyl plane of the left lever.

Next, we make efforts to replace 1-ethynyl-4-methylphenyl lever by using (*R*)-4-*N*-(1-phenylethyl)benzamide counterpart, which has been widely employed for multi-layer chirality investigation. Similarly, the stable atropisomer (**IIIA**) has been designed, synthesized, and analyzed by X-ray diffraction analysis. In this atropisomer, the sulfinyl amino group is also pushed away from the blocking group, (*R*)-4-*N*-(1-phenylethyl)amide on benzene ring of the left arm (Figure 5).

**FIGURE 7:**
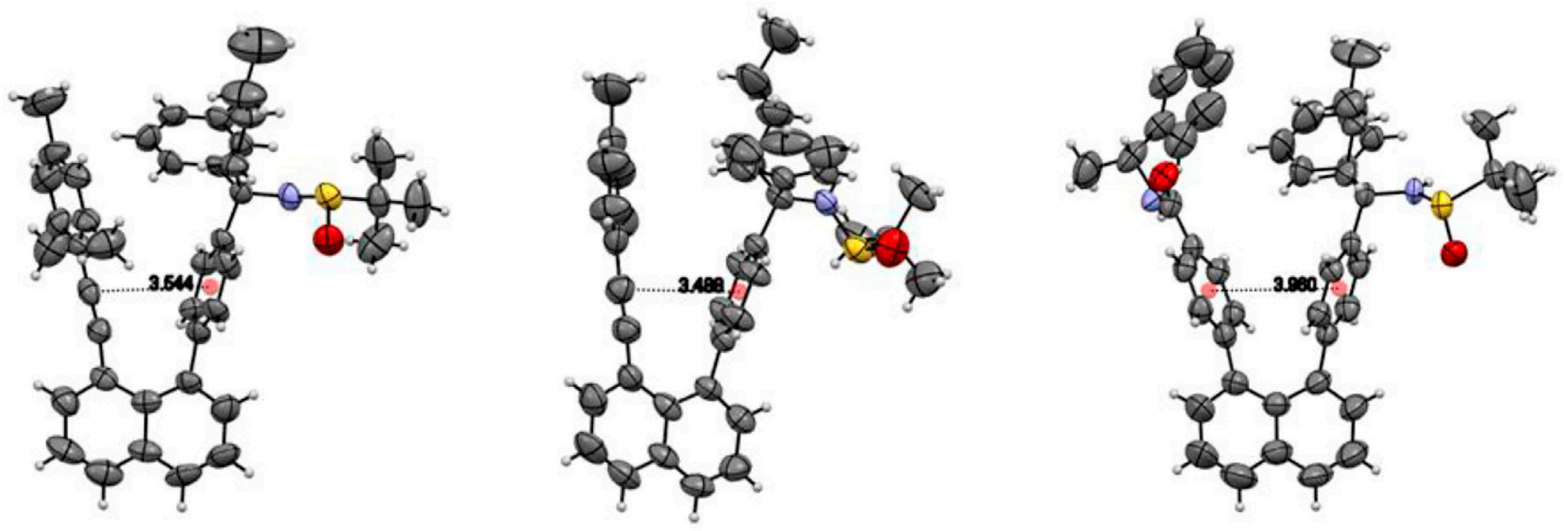
Comparison of distances of anchor centers.

Lines were drawn and measured between two centers of phenyl rings on two arms/levers parallel to the line between 1,8-positions of naphthalenyl anchor. The distance from two ring centers of (**IIIA)** was measured to be 3.960 Å. Similar lines of (**IA**) and (**IIA**) were drawn for the comparing purpose, which is also nearly parallel to that between 1,8-positions of naphthalenyl ring. These two lines were measured to be 3.544 Å and 3.488 Å, respectively. Interestingly, the two levers/arms in (**IIIA**) are more widely opened than those in (**IA**) and (**IIA**), but chiral carbon’s free rotation cannot occur in (**IIIA**). This is due to the fact that (*R*)-4-*N*-(1-phenylethyl)amide is much bulkier than the methyl group, particularly, the phenyl ring on the amide group is directed toward the chiral center side. This phenyl ring could partially shield groups on sp^3^ carbon, making it even more difficult to rotate (Figure 7).

## 3 Orientational chirality model

**FIGURE 8:**
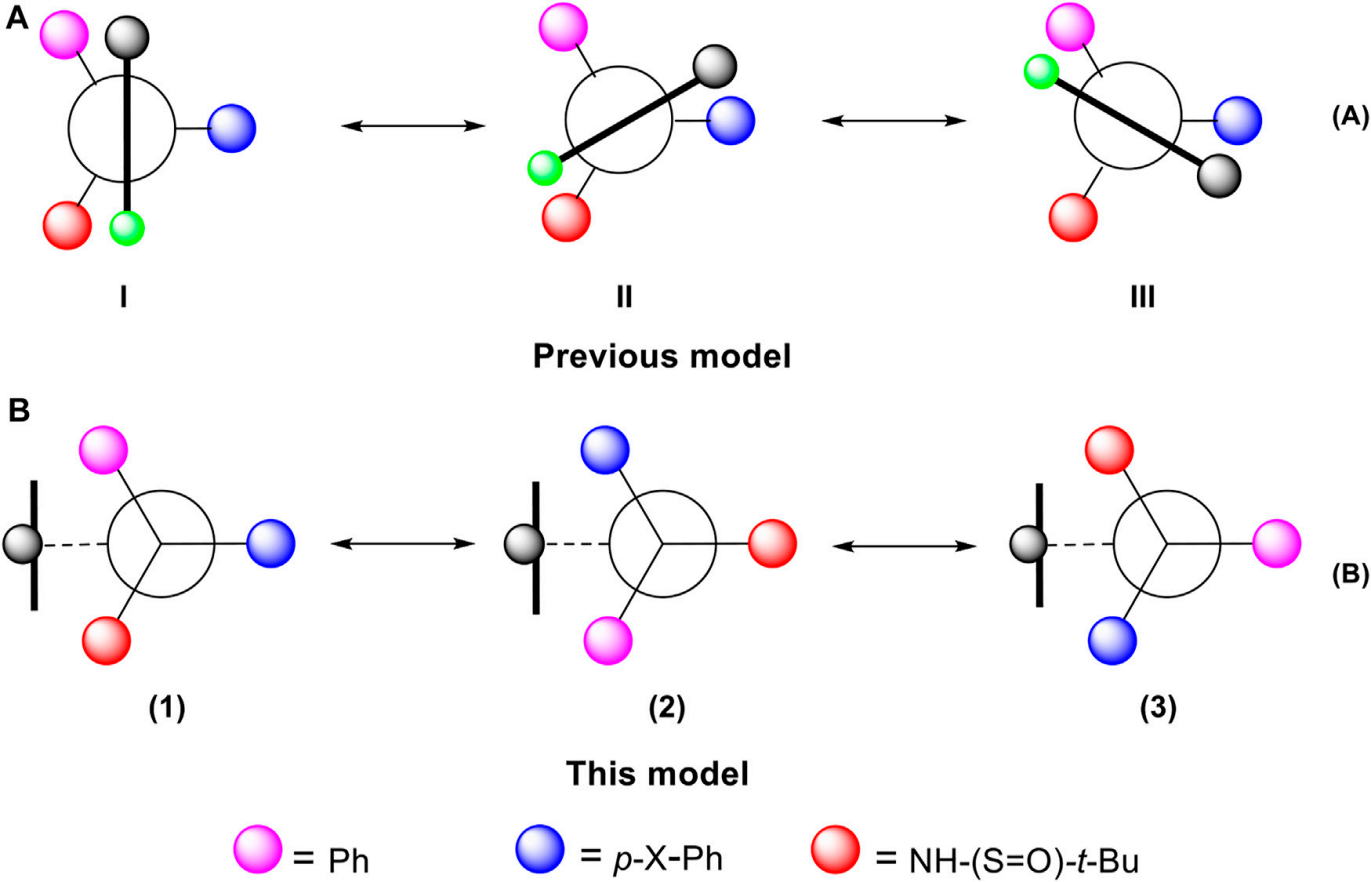
Orientantion chirality models: previous I-III and present 1-3.

The conformationally stable architectures containing axial C (sp^2^)− C (sp^3^) stereogenic axis was achieved *via* asymmetric catalytic [2 + 2 + 2] cyclotrimerization leading to forming one of six rotamers (Wu X. et al., 2021). The resulting axial C (sp^2^)−C (sp^3^) atropisomers have been proven by X-ray diffraction analysis to follow Felkin-Ahn-type model, *i.e*, one of the three groups on C (sp3) is arranged perpendicularly to C (sp^2^) plane (Figure 8A). There are six energy barriers existing during the rotating process. *This multi-fold chirality is focused on the dialog relationship between two adjacent blocking C(sp* ^*2*^ *) and chiral C(sp* ^*3*^ *) scaffolds*. However, in our chirality framework, there is no direct controlling force between chiral C (sp^3^) stereogenicity and blocking C (sp^2^) subunit.The remotely anchored aromatic ring is the only functional group that blocks rotation along C (sp^2^)− C (sp^3^) axis (Figure 8B). Therefore, *the present orientational chirality is focused on the dialog relationship between C(sp* ^*3*^ *) center and a remotely anchored functional group*. Since there is only a single interaction (the heavy black line in the model, Figure 8B) existing in each of the three atropisomers, there are three energy barriers instead of six in the previous atropisomerism to be expected.

It should be pointed out that the nomenclature of previous molecular architectures follows the Cahn−Ingold−Prelog (CIP) rules (Tang et al., 2022b; Jin et al., 2022). However, finding a nomenclature rule for the present chirality framework seems difficult. The relationship among three atropisomers (I, II and III in Figure 8A) would not belong to the classical enantiomeric or diastereomeric isomerism. For comparison, a representative example is given in Figure 8B for three atropisomers, (1)–(3). In these cases, there exist three pairs of enantiomers and six pairs of diastereomers, which is very rare in stereochemistry.

Consequently, the stereochemical measurements for the atropisomers, (1), (2) and (3), would not fit the classical *ee*/*er* or *de*/*dr* descriptions. Therefore, new descriptions would be temporally suggested for measuring outcomes of asymmetric synthesis and catalysis for assembling these three chiral atropisomers, *e.g*, orientatiomeric selectivity of orientatiomeric excess (*oe*) and orientatiomeric ratios (*or*) would be utilized, respectively.

## 4 Asymmetric synthesis

**SCHEME 1:**
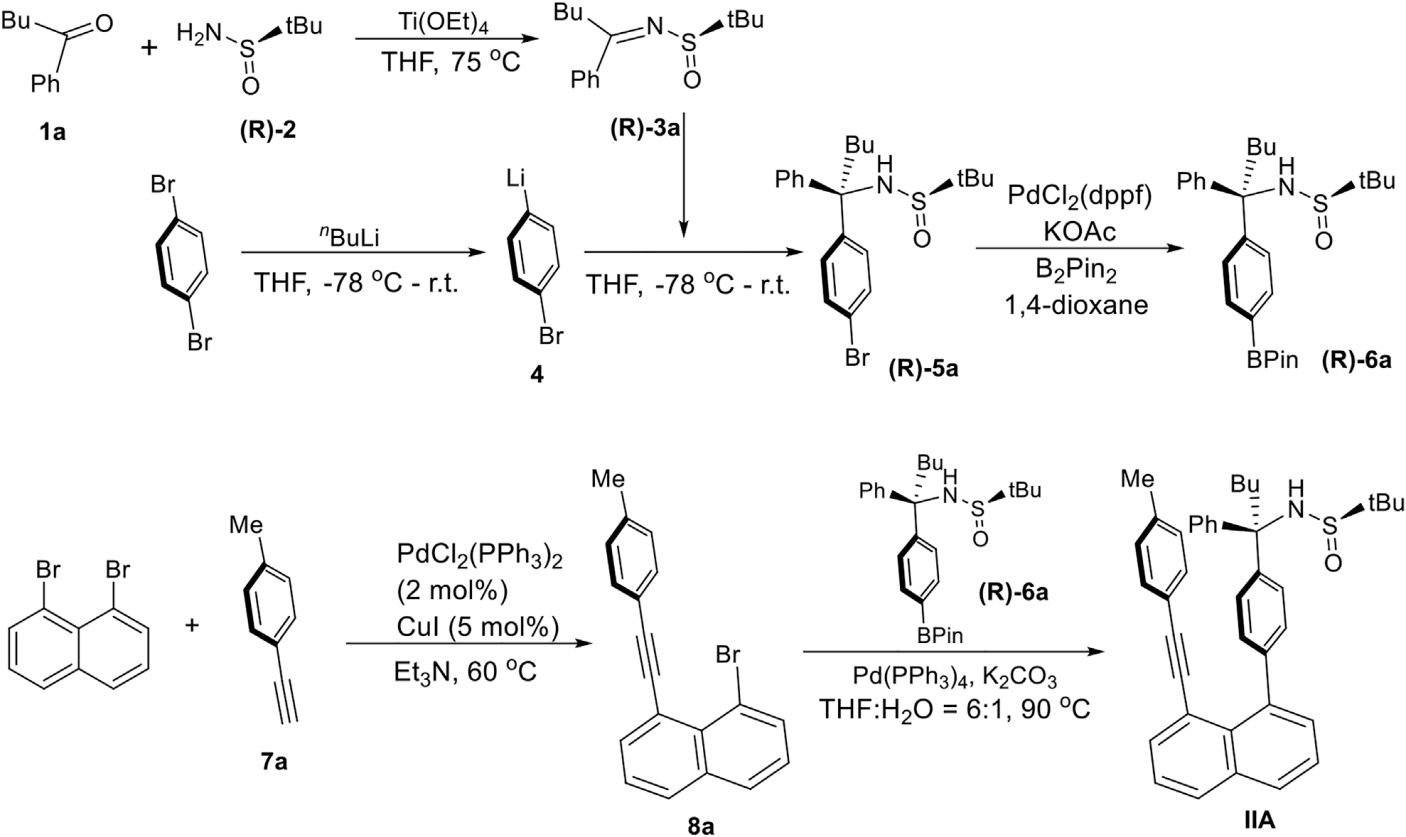
Asymmetric synthesis of orientational chirality with different anchors.

Asymmetric syntheses of atropisomers **IIA** are represented by assembling (*R*)-2-Methyl-*N*-((*R*)-1-phenyl-1-(4-(8-(p-tolylethynyl)naphthalen-1-yl)phenyl)pentyl)propane-2-sulfinamide (**(R)-6a**) (Scheme 1). The preparation of (*R*)-2-methyl-*N*-(1-phenylpentylidene)propane-2-sulfinamide (**(R)-3a**) was performed by dehydration of 1-phenylpentan-1-one (**1a**) with (*R*)-2-methylpropane-2-sulfinamide (**(R)-2**) by using Ti(OEt)_4_ in dry THF at 75°C to room temperature to give 93% yield (Zhang et al., 2019). 1,4-Dibromobenzene was converted into (4-bromophenyl)lithium precursor *via* the treatment with n-BuLi in THF at the same temperatures followed by reacting with (*R*,Z)-2-methyl-*N*-(1-phenylpentylidene)propane-2-sulfinamide (**(R)-3a**) to give (*R*)-*N*-((*R*)-1-(4-bromophenyl)-1-phenylpentyl)-2-methylpropane-2-sulfinamide (**(R)-5a**, 75% yield), which was then transformed into its BPin derivative (**(R)-6a**) by reacting with B_2_Pin_2_ in the presence of PdCl_2_(dppf) as the catalyst and KOAc as an additive in 1,4-dioxane to give a yield of 58%.

1,8-Dibromonaphtalene was subjected to Sonogashira coupling reaction (Sonogashira, 2002) with 1-ethynyl-4-methylbenzene in the presence of PdCl_2_(PPh_3_)_2_ and Cu(I) iodide as co-catalysts in Et_3_N solution to afford 1-bromo-8-(p-tolylethynyl)naphthalene (**8a**) in 74% chemical yield. Suzuki-Miyaura cross-coupling (Miyaura and Suzuki, 1995) of 1-bromo-8-(p-tolylethynyl)naphthalene (**8a**) with (*R*)-2-methyl-*N*-((*R*)-1-phenyl-1-(4-(4,4,5,5-tetramethyl-1,3,2-dioxaborolan-2-yl)phenyl)pentyl)propane-2-sulfinamide (**(R)-6a**) resulted in the final product, (*R*)-2-methyl-N-((*R*)-1-phenyl-1-(4-(8-(p-tolylethynyl)naphthalen-1-yl)phenyl)pentyl)propane-2-sulfinamide (**IIA**) in a yield of 67%.

**SCHEME 2:**
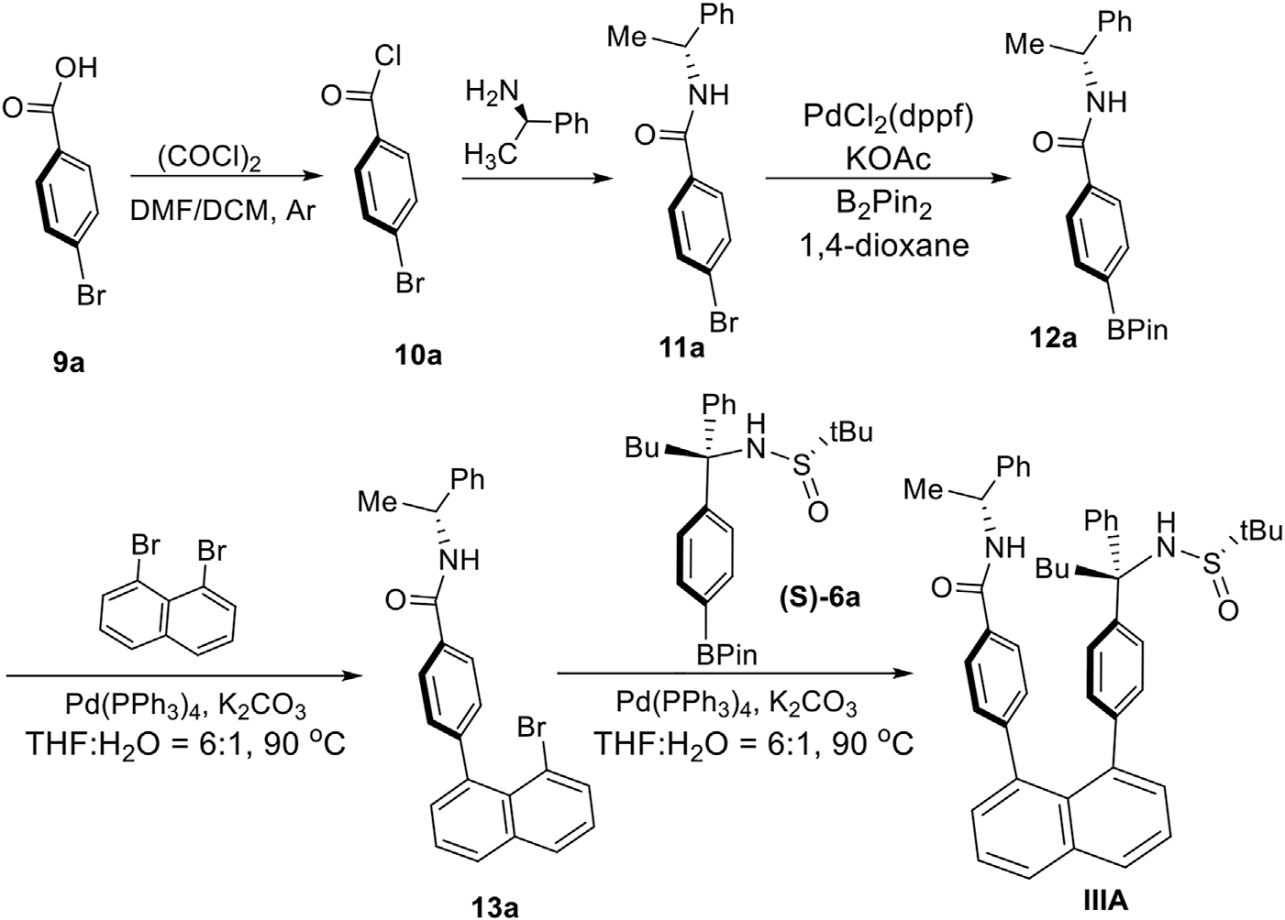
Asymmetric synthesis of orientational chirality with two aryl anchors.

Atropisomers **IIIA** was assembled from three building blocks (Scheme 2): (*R*)-*N*-(1-phenylethyl)-4-(4,4,5,5-tetramethyl-1,3,2-dioxaborolan-2-yl)benzamide (**12a**), 1,8-dibromonaphtalene, and (*S*)-2-methyl-*N*-((*S*)-1-phenyl-1-(4-(4,4,5,5-tetramethyl-1,3,2-dioxaborolan-2-yl)phenyl)pentyl)propane-2-sulfinamide (**(S)-6a**). The synthesis of (*S*)-2-methyl-N-((*S*)-1-phenyl-1-(4-(4,4,5,5-tetramethyl-1,3,2-dioxaborolan-2-yl)phenyl)pentyl)propane-2-sulfinamide (**(S)-6a**) is similar to that described above for **IIA**. (*R*)-4-(8-Bromonaphthalen-1-yl)-N-(1-phenylethyl)benzamide (**13a**) was obtained by reacting (*R*)-*N*-(1-phenylethyl)-4-(4,4,5,5-tetramethyl-1,3,2-dioxaborolan-2-yl)benzamide (**12a**) with 1,8-dibromonaphtalene under the Suzuki-Miyaura cross-coupling condition (Miyaura and Suzuki, 1995). The precursor (**12a**) was generated by treating (*R*)-4-bromo-N-(1-phenylethyl)benzamide with B_2_Pin_2_ in the presence of PdCl_2_(dppf) as the catalyst and KOAc as an additive in 1,4-dioxane to give a yield of 54%.

**FIGURE 9:**
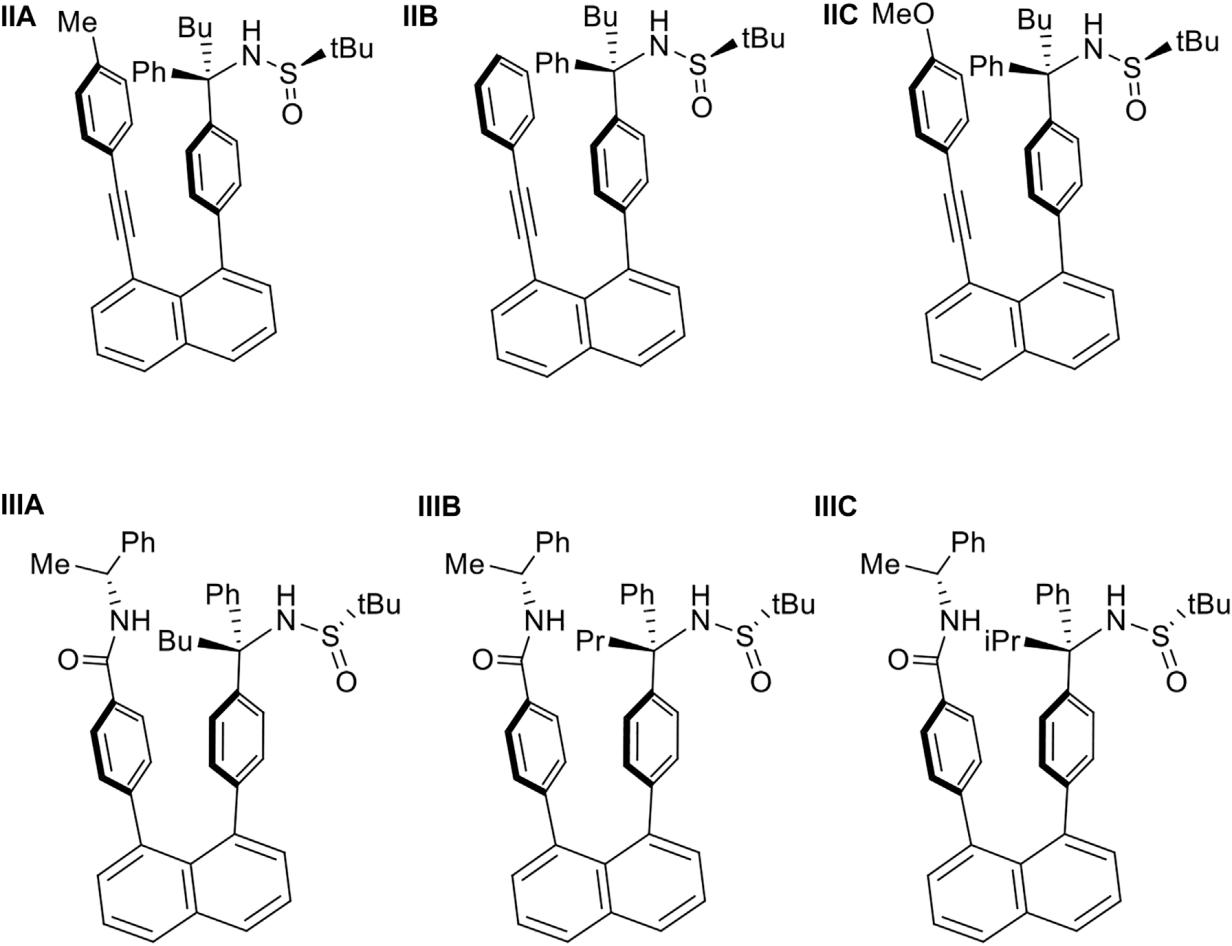
Targets of orientational chirality: **(IIA‐IIC)** and **(IIIA-IIIC)**.

According to the above procedures, the derivatives of **IIA** and **IIIA** were synthesized as the two pairs of **IIB**/**IIC** and **IIIB**/**IIIC**, respectively (Figure 9). Not only *p*-MeO substituent on benzene ring is anticipated to block the rotation along the C (chiral sp^3^)-C (sp^3^) bond as *p*-Me group does, but also the none-substituent benzene group (*p*-H) still can perform blocking as revealed by spectra analysis. For cases **IIIB** and **IIIC** in which n-butyl group in **IIIA** was replaced by propyl and isopropyl groups, respectively, showed stable rotamers as expected.

**FIGURE 10:**
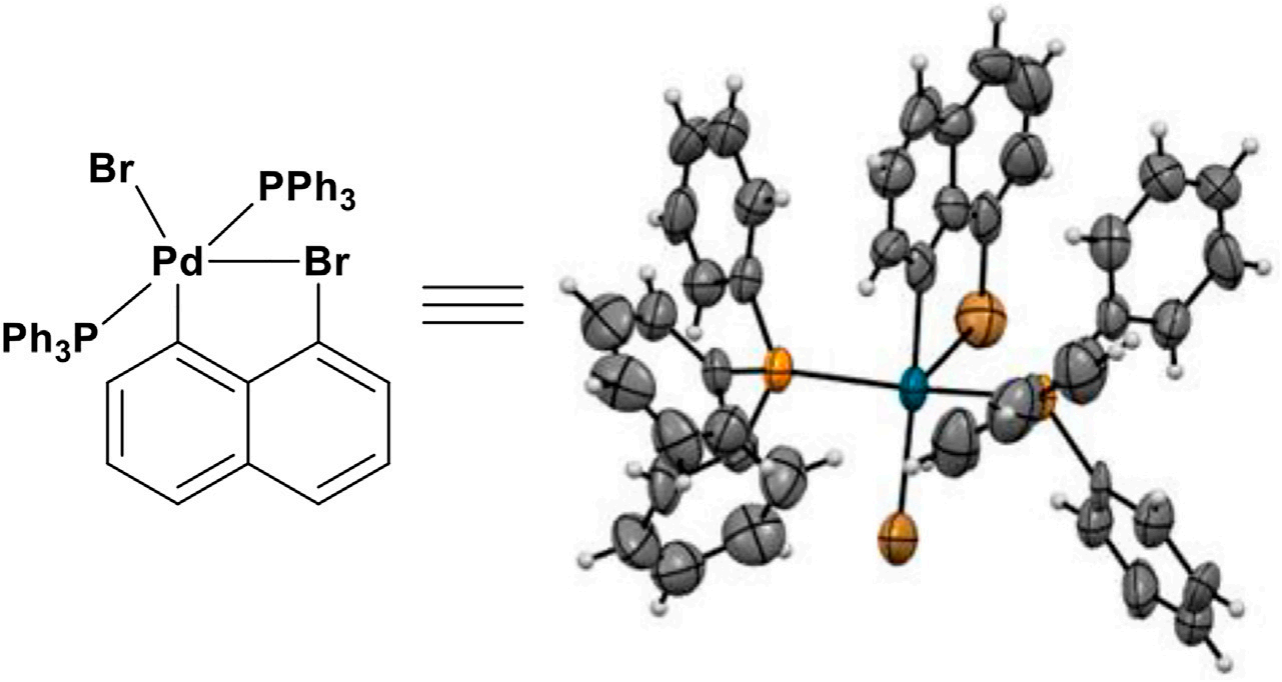
X-ray structure of a new catalytic species.

During the Suzuki-Miyaura cross-coupling involving 1,8-dibromonaphtalene, a key catalytic species was successfully isolated. Its x-ray diffraction analysis revealed that this metal-ligand species displays a five-membered ring system consisting of C (sp^2^)-Br-Pd-C (sp^2^) bonds (Figure 10), which has not been documented in the literature to the best of our knowledge. A CSD search revealed that there has been only one similar catalytic species of five-membered ring complex formed during the catalytic synthesis of tetrahydrobenzo [b]azepines (THBAs), but it is based on C (sp^3^)-Br-Pd-C (sp^3^) bonds (Liu X. et al., 2021). We believe this new catalytic complex would find applications in future, especially, when two phosphine ligands are replaced by their chiral counterparts (Figure 10).

## 5 Computational Studies

### 5.1 Computational details

**FIGURE 11:**
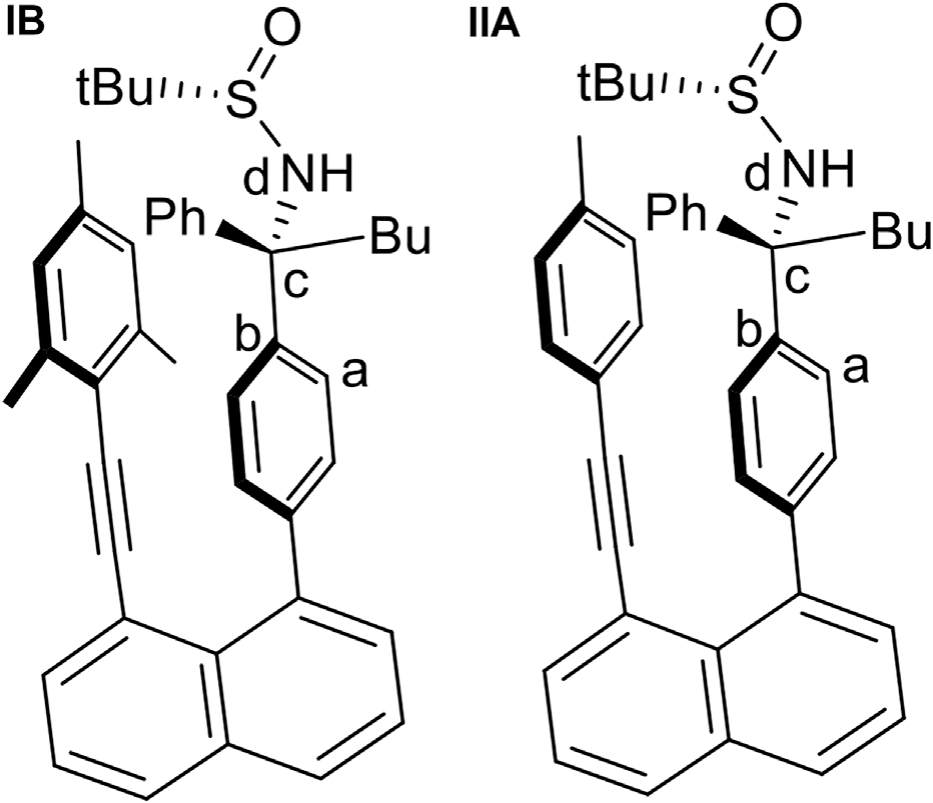
The two initial structures, **(IB)** and **(IIA)**, used for geometry optimization and the definition of the torsional angle τ(a,b,c,d) used for the calculation of the rotational potential energy curves.

The quantum chemical calculations have been performed at density functional theory (DFT) level using the Becke, 3-parameter, Lee–Yang–Par (B3-LYP functional including the D3 dispersion correction (Becke, 1993; Grimme et al., 2010) and the 6-31G* basis set (Hariharan and Pople, 1973). Calculations were performed for a solvent consisting of a 1:1 mixture of tetrahydrofuran and water with an average dielectric constant ε of 42.89. The crystal structure of Structure **IB** (Figure 11) was used as starting geometry for a full geometry optimization. Starting from this geometry, the torsional angle τ defined in Figure 11 was increased in steps of 20° to obtain an energy profile on the different rotated structures. Keeping τ fixed for each value, the remaining geometry was optimized, and potential energy curves (PECs) were computed. Using the geometries of the minima in the PEC as starting points, full geometry optimizations were performed. The PEC for structure **IIA** (Figure 11) in which the mesityl group was replaced by the p-methylphenyl group (see also structure 9aa, Figure 5) has been obtained in an analogous way. All calculations were carried out using the Gaussian 09 program suite (Gaussian 09 Citation, 2022).

### 5.2 Theoretical results

The PEC (Supplementary Figures S1, S2 of the Supplementary Material S1) for the torsion around the bc bond (Figure 11) for structure **IB** shows an energy profile with several minima. The most stable ones are found for τ values of -25° and -165°. Another one is located at 55° with a relative energy of ∼3.5 kcal/mol. The region with positive τ values shows a broad peak with a height of around 6–8 kcal/mol and a shallow minimum at 55°. The structures corresponding to these three minima are displayed in Supplementary Figure S2. The analogous PEC for structure II (Supplementary Figure S3) looks similar with the difference that the broad region of positive torsional values has only shallower formation of two minima.

**FIGURE 12:**
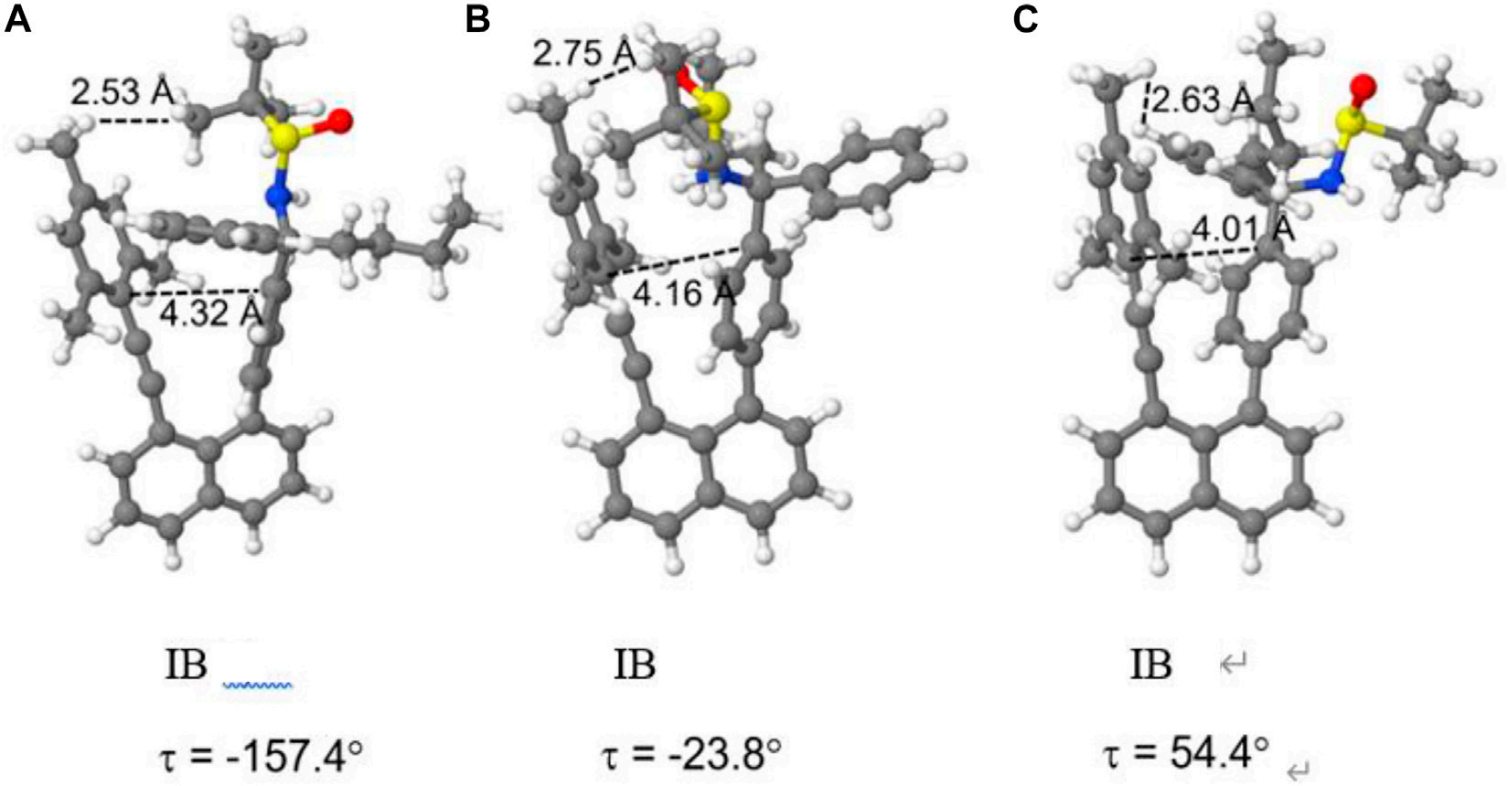
Fully optimized structures of the three rotamers of structure **IB** **(A–C)**.

**FIGURE 13:**
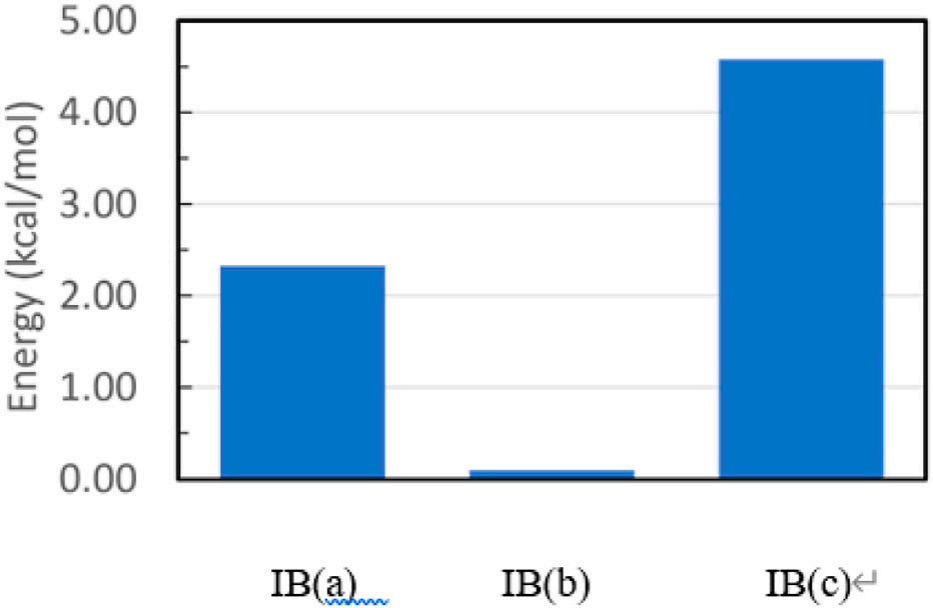
Relative energies of the three fully optimized geometries for Structure **(IB)**.

For structure **IB**, the three geometries obtained by restricted optimization were used as starting points for complete optimization without any restriction in the torsional angle τ. This optimization led to the final three rotamer structures displayed in Figure 12. The corresponding energetic stabilities are given in Figure 13. The most stable structure is **IB(b)** where the butyl and sulfamide groups interact with the opposite benzene ring and the phenyl ring is turned to the outside. The next stable structure (at relative energy of 2.3 kcal/mol) is **IB(a)** showing the interaction of the t-butyl group and the phenyl ring in contact with the opposite benzene ring. Finally, the least stable structure at 4.6 kcal/mol is the one with the butyl and phenyl ring oriented toward the opposite benzene ring.

**FIGURE 14:**
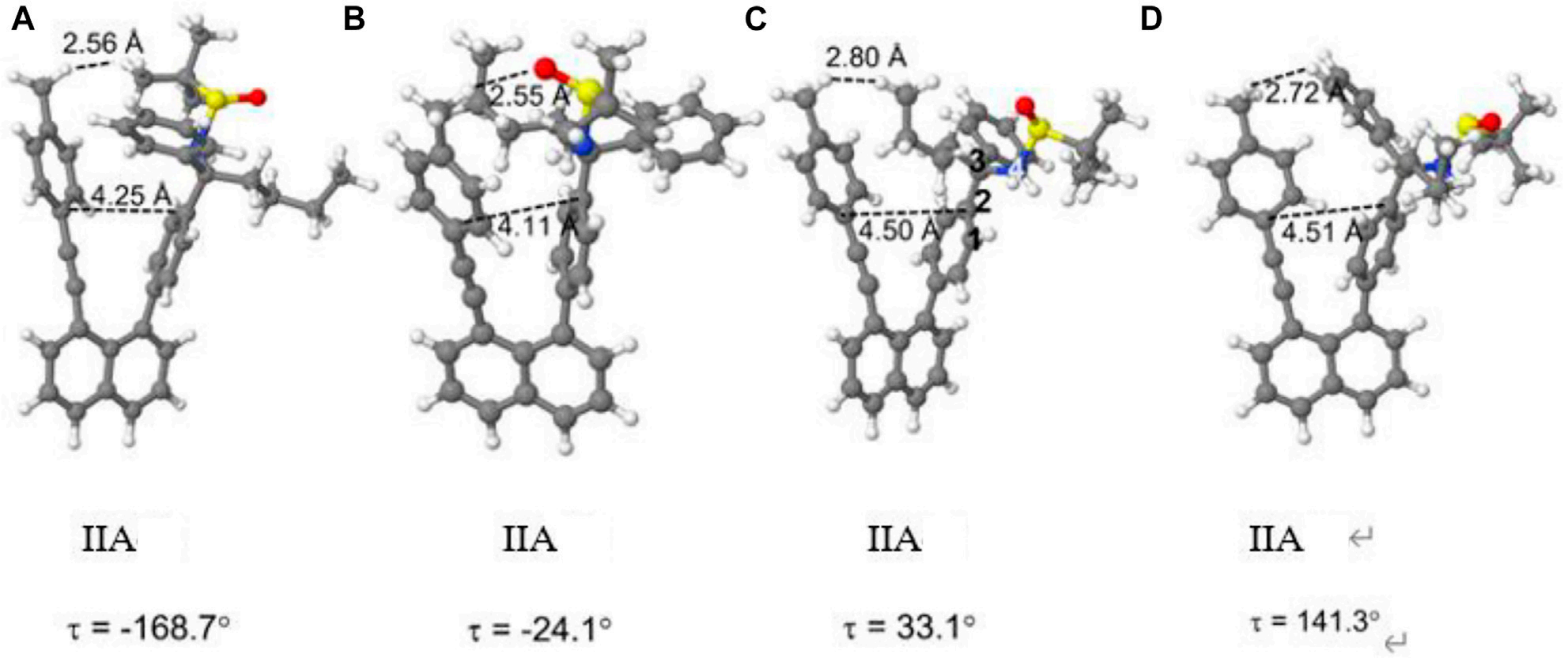
Fully optimized structures of the four rotamers of structure **IIA**.

**FIGURE 15:**
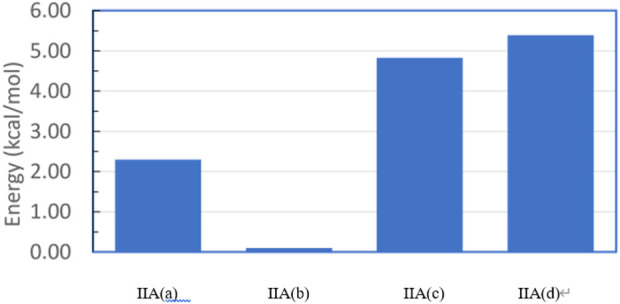
Relative energies of the four fully optimized geometries for structure **IIA**.

The PEC for structure **IIA** shows a similar, but slightly more extended manifold of energetic minima and structures (Supplementary Figures S3, S4). Full geometry optimization of these structures shows a similar, but slightly more extended manifold of energetic minima (Figure 14). Structures IIA (a-c) resemble closely the three structures **IB** cases. Structure **IIA(b)** is the most stable structure (Figure 15), followed by structures **IIA(a)** and **IIA(c)**. The fourth structure **IIA(d)**, is slightly higher in energy than **IIA(c)**.

For both structures **IB** and **IIA**, the most stable structure is of type (b) where the polar S=O bond is directly oriented toward the opposite phenyl ring, interacting with its aromatic π system. In both cases, the second stable structure involves the sulfinyl-amino group as well; it is, however, not directly oriented toward the opposing phenyl ring. In the least stable structures, the sulfinyl-amino group is oriented completely away from this phenyl ring. Selected non-bonded distances are displayed in Figures 12, 14 as well. Most interesting is the comparison of the distance of the mesityl group to the opposing ring in comparison to case where two of the methyl groups had been removed. For the most stable structures (a) and (b), the distance between the two rings, as measured by the C···C distances shown in Figures 12, 14, is larger for the mesitylene substitution (4.32 Å vs. 4.25 Å for structures (a), and 4.16 Å vs. 4.11 Å for structures (b) indicating the repulsive effect of the bulkier methyl groups pushing the two aromatic rings a bit farther away. This structural opening up due to the methyl groups agrees well with the widening between the two levers/arms as shown in Figure 6 for the X-ray structures.

## 6 Summary

We have discovered the orientational chirality showing that multiple orientations can be controlled by remotely anchored and through-space functional blockers. The multi-step synthesis of several orientational chiral targets were achieved by conducting asymmetric nucleophilic addition, Suzuki-Miyaura cross-coupling and Sonogashira coupling reactions. Single orientational atropisomers were obtained in modest to good yields as crystalline solids. A novel catalytic complex was isolated during performing Suzuki-Miyaura cross-coupling, and analyzed by X-ray diffraction analysis displaying a five-membered ring consisting of C (sp^2^)-Br-Pd-C (sp^2^) bonds. The present orientational chirality is focused on the dialog relationship between C (sp^3^) center and a remotely anchored functional group. X-ray structures of orientational chiral targets leads to a conceptually new stereochemistry model which is differentiated from the previous Felkin-Ahn-type of models containing adjacent C (sp^2^)-C (sp^3^) σ bonds. In this new model, there are three main energy barriers during orientational rotation instead of six barriers in previous multi-fold systems. DFT computational study was carried out, and present the relative stability with rotating of individual orientatiomers. This discovery would be anticipated to result in a new stereochemistry area, and to have a broad impact on chemical, biomedical and material sciences in future.

## Data Availability

The original contributions presented in the study are included in the article/Supplementary Material, further inquiries can be directed to the corresponding authors.
