# Supplementary material for: A new chiral phenomenon of orientational chirality, its synthetic control and computational study
Source: Front Chem. 2023 Jan 5;10:1110240. doi: 10.3389/fchem.2022.1110240 (PMC9850238; doi:10.3389/fchem.2022.1110240)
Supplement: Supplementary file 1 [file DataSheet1.PDF]

*Supplementary Material*

# A New Chiral Phenomenon of Orientational Chirality, its Synthetic Control and Computational Study

**Shengzhou Jin,[‡,§] Ting Xu,[‡,§] Yao Tang,[†,§] Jia-Yin Wang,[α] Yu Wang,[‡] Junyi Pan,[‡] Sai Zhang,[†] Qingkai Yuan,[†] Anis Ur Rahman,[†] Adelia J. A. Aquino,[#] Hans Lischka, [†,]* and Guigen Li*[,†,‡]**

[†]Department of Chemistry and Biochemistry, Texas Tech University, Lubbock, Texas 79409-1061, USA.

[‡]School of Chemistry and Chemical Engineering, Nanjing University, Nanjing, 210093, China.

[α]Continuous Flow Engineering Laboratory of National Petroleum and Chemical Industry, Changzhou University, Changzhou, Jiangsu 213164, China.

[#]Department of Mechanical Engineering, Texas Tech University, Lubbock, TX, 79409, USA

[§]These authors made equal contributions to this work.

*** Correspondence:** Guigen Li: guigen.li@ttu.edu; Hans Lischka: hans.lischka@univie.ac.at

# 1 General Information

Unless otherwise stated, all reactions were magnetically stirred and conducted in oven-dried glassware in anhydrous solvents under Ar. Solvents and liquid reagents, as well as solutions of solid or liquid reagents were added directly or via syringes, or micropipette. Cooling baths were prepared in Dewar vessels filled with ice/water (0 °C). Heated oil baths were used for reactions requiring elevated temperatures. Solvents were removed under reduced pressure at 40-65 °C using a rotavapor. All given yields for small molecules are isolated yields of chromatographically and NMR spectroscopically materials.

All commercially available chemicals were used as received without further purification. Solvents as follows: MeOH, EtOH, toluene, EtOAc, DCM, dioxane, hexane, acetone and THF were used without further purification.

The $^{1}H$ and $^{13}C$ NMR spectra were recorded in $CDCl_3$ on 400 MHz and 100 MHz instruments with TMS as internal standard. For referencing of the $^{1}H$ NMR spectra, the residual solvent signal ($\delta = 7.26$ for $CDCl_3$) were used. In the case of the $^{13}C$ NMR spectra, the signal of solvents ($\delta = 77.06 \pm 0.03$ for $CDCl_3$) were used. Chemical shifts(δ) were reported in ppm with respect to TMS. Data are represented as follows: chemical shift, multiplicity (s = singlet, d = doublet, t = triplet, m = multiplet), coupling constant (*J*, Hz), and integration. MALDI-TOF analyses were carried out using an ABI/MDS SCIEX 4800 Mass Spectrometer with HABA matrix. UV-Vis spectra were collected on an Agilent 8453 UV-Visible Spectroscopy system. Fluorescence spectra were collected by Agilent Technologies Cary Eclipse Fluorescence Spectrophotometer G9800A and Eclipse ADL program.

X-ray data were collected on a Rigaku XtaLAB Synergy-*i* Kappa diffractometer equipped with a PhotonJet-*i* X-ray source operated at 50 W (50kV, 1 mA) to generate Cu Kα radiation (λ = 1.54178 Å) and a HyPix-6000HE HPC detector. Crystals were transferred from the vial and placed on a glass slide in polyisobutylene. A Zeiss Stemi 305 microscope was used to identify a suitable specimen for X-ray diffraction from a representative sample of the material. The crystal and a small amount of the oil were collected on a MīTiGen cryoloop and transferred to the instrument where it was placed under a cold nitrogen stream (Oxford 700 series) maintained at 100K throughout the duration of the experiment. The sample was optically centered with the aid of a video camera to insure that no translations were observed as the crystal was rotated through all positions. A unit cell collection was then carried out. After it was determined that the unit cell was not present in the CCDC database a data collection strategy was calculated by *CrysAlis*$^{Pro}$[1]. The crystal was measured for size, morphology, and color. These values were reported in Tables.

# 2 Synthetic and Analysis

## 2.1 General procedure for the synthesis of substrates 3a

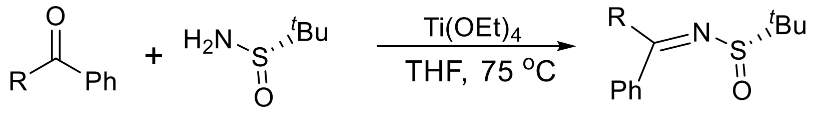


**1** **(S)-2** **3**

To a solution of (*S*)-(+)-2-methyl-2-propane sulfinamide (0.6 g, 5 mmol, 1 eq) and aryl ketones (6 mmol, 1.2 eq) in anhydrous THF (30 mL) at room temperature was added $Ti(OEt)_4$ (6 mL, 15 mmol,

3 eq). The mixture was heated at 75 °C for 24 h. After cooling to room temperature, the mixture was poured into 30 mL of brine under vigourous stirring. The resulting suspension was filtered through a pad of Celite and the solid was washed with EtOAc (3 x 20 mL). The filtrate was washed with brine (30 mL), dried ($Na_2SO_4$), and concentrated in vacuo. The residue was chromatographed on silica by eluting with PE/EA (20:1 to 10:1) to get the pure product.

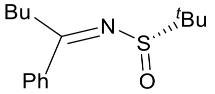

**(S)-3a**

***(S, Z)*-2-methyl-*N*-(1-phenylpentylidene)propane-2-sulfinamide ((S)-3a)**: Purified by using a flash column chromatography (PE/EA = 15/1); isolated yield = 91%, 1.2077 g; yellow liquid. $^1$H NMR (400 MHz, Chloroform-*d*) 7.83 (d, *J* = 5.2 Hz, 2H), 7.46–7.39 (m, 3H), 3.21 (d, *J* = 43.6 Hz, 2H), 1.67–1.59 (m, 2H), 1.48–1.42 (m, 2H), 1.31 (s, 9H), 0.93 (t, *J* = 7.2 Hz, 3H) $^{13}$C NMR (101 MHz, Chloroform-*d*) 138.0, 131.4, 128.5, 127.5, 57.3, 32.4, 30.7, 23.0, 22.6, 13.7.

The product of **(*R*, *Z*)-2-methyl-N-(1-phenylpentylidene)propane-2-sulfinamide ((R)-3a)**: was synthesized in the same way as above.

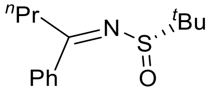

**(S)-3b**

**(*S*, *Z*)-2-methyl-N-(1-phenylbutylidene)propane-2-sulfinamide ((S)-3b)**: Purified by using a flash column chromatography (PE/EA = 20/1); isolated yield = 90%, 1.1313 g; yellow liquid. $^1$H NMR (400 MHz, Chloroform-*d*) $\delta$ 7.82 (s, 2H), 7.47–7.39 (m, 3H), 3.23–3.14 (m, 2H), 1.78–1.65 (m, 2H), 1.31 (s, 9H), 1.02 (t, *J* = 7.2 Hz, 3H). $^{13}$C NMR (101 MHz, Chloroform-*d*) $\delta$ 180.1, 138.0, 131.4, 128.5, 127.4, 57.4, 34.4, 22.6, 22.2, 14.2.

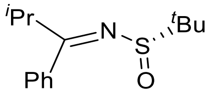

**(S)-3c**

**(*S*, *Z*)-2-methyl-N-(2-methyl-1-phenylpropylidene)propane-2-sulfinamide ((S)-3c)**: Purified by using a flash column chromatography (PE/EA = 20/1); isolated yield = 87%, 1.0935 g; yellow liquid. $^1$H NMR (400 MHz, Chloroform-*d*) $\delta$ 7.43–7.38 (m, 3H), 7.29 (d, *J* = 6.4 Hz, 2H), 3.03 (s, 1H), 1.21 (d, *J* = 6.1 Hz, 15H). $^{13}$C NMR (101 MHz, Chloroform-*d*) $\delta$ 189.8, 138.1, 129.3, 128.1, 126.7, 56.2, 22.1, 20.1, 19.7.

### 2.2 General procedure for the synthesis of substrates 5

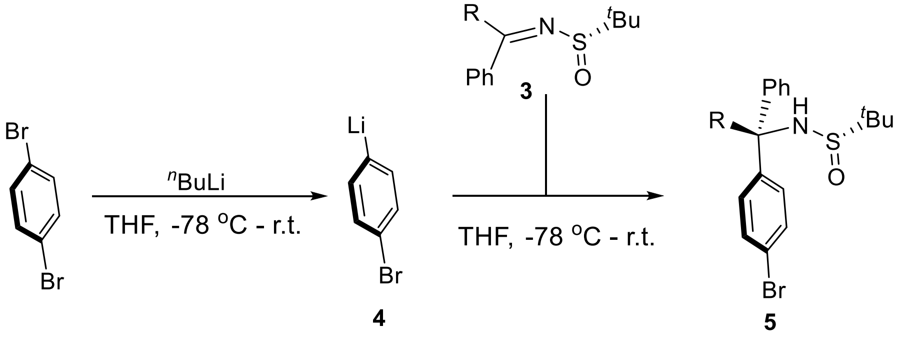

To a solution of 1,4-Dibromobenzene (2.3590 g, 10 mmol) in dry THF (30 mL) at -78 °C was added *n*-butyllithium (6.25 mL, 1.6 M in hexanes). The resulting solution was stirred for 2 hours, a solution of **3** (10 mmol, 1 equiv) in dry THF (15 mL) was slowly added via syringe. Stirring was continued at –78 °C for 2 hours, and then heat the solution to room temperature for 14 hours. Subsequently, the saturated aqueous $NH_4Cl$ was added to the solution, the organic phase was extracted with EA and dried over anhydrous $MgSO_4$. Chromatography (PE: EA= 10:1 - 6:1) afforded the diastereomerically pure propargyl **5**.

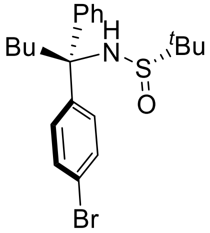


**(S)-5a**

**(*S*)-*N*-((*S*)-1-(4-bromophenyl)-1-phenylpentyl)-2-methylpropane-2-sulfinamide ((S)-5a)**: Purified by using a flash column chromatography (PE/EA = 6/1); isolated yield = 78%, 3.2950 g; yellow liquid. $^1$H NMR (400 MHz, Chloroform-*d*) $\delta$ 7.42 (d, $J$ = 8.8 Hz, 2H), 7.33–7.26 (m, 5H), 7.15 (d, $J$ = 8.8 Hz, 2H), 3.97 (s, 1H), 2.62–2.54 (m, 1H), 2.51–2.43 (m, 1H), 1.37–1.30 (m, 3H), 1.22 (s, 9H), 1.01–0.94 (m, 1H), 0.85 (t, $J$ = 7.2 Hz, 3H). $^{13}$C NMR (100 MHz, Chloroform-*d*) $\delta$ 145.6, 144.5, 131.4, 128.4, 128.1, 128.0, 127.9, 127.4, 120.9, 66.1, 56.5, 39.7, 25.8, 22.9, 22.8, 22.6, 13.9.

The product of **(*R*)-*N*-((*R*)-1-(4-bromophenyl)-1-phenylpentyl)-2-methylpropane-2-sulfinamide ((R)-5a)** was synthesized in the same way as above.

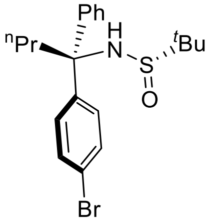


**(S)-5b**

**(*S*)-*N*-((*S*)-1-(4-bromophenyl)-1-phenylbutyl)-2-methylpropane-2-sulfinamide ((S)-5b)**: Purified by using a flash column chromatography (PE/EA = 10/1); isolated yield = 75%, 3.0630 g; yellow liquid. $^1$H NMR (400 MHz, Chloroform-*d*) $\delta$ 7.44 (d, $J$ = 8.8 Hz, 2H), 7.34–7.26 (m, 5H), 7.17 (d, $J$ = 8.8 Hz, 2H), 4.00 (s, 1H), 2.62–2.54 (m, 1H), 2.50–2.43 (m, 1H), 1.35–1.31 (m, 1H), 1.24 (s, 9H), 1.09–0.99 (m, 1H), 0.94 (t, $J$ = 7.2 Hz, 3H). $^{13}$C NMR (101 MHz, Chloroform-*d*) $\delta$ 145.6, 144.5, 131.4, 128.4, 128.1, 128.0, 127.4, 120.9, 66.2, 56.5, 42.2, 22.8, 17.1, 14.2.

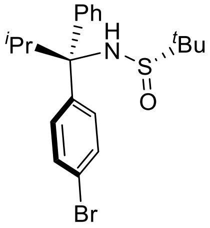


**(S)-5c**

**(S)-*N*-((*S*)-1-(4-bromophenyl)-2-methyl-1-phenylpropyl)-2-methylpropane-2-sulfinamide((S)-5c)**: Purified by using a flash column chromatography (PE/EA = 10/1); isolated yield = 80%, 3.2672

g; yellow liquid. $^1$H NMR (400 MHz, Chloroform-*d*) $\delta$ 7.47 (d, *J* = 8.4 Hz, 2H), 7.36–7.30 (m, 4H), 7.29–7.25 (m, 3H), 4.02 (s, 1H), 3.19–3.10 (m, 1H), 1.17 (s, 9H), 0.96 (d, *J* = 6.4 Hz, 3H), 0.88 (d, *J* = 6.4 Hz, 3H). $^{13}$C NMR (101 MHz, Chloroform-*d*) $\delta$ 137.9, 132.6, 130.3, 128.1, 128.0, 127.6, 126.6, 119.1, 56.0, 28.2, 22.7, 21.4, 19.5.

### 2.3 General procedure for the synthesis of substrates 6

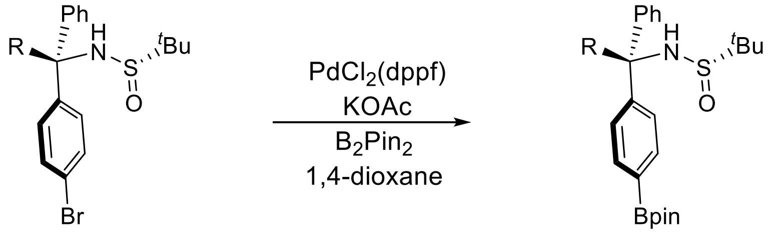


**(S)-5** **(S)-6**

The mixture of **(S)-5** (3.8 mmol, 1 equiv), bis(pinacolato)diboron (2.2918 g, 9.5 mmol, 2.5 equiv), potassium acetate (1.1187 g, 11.4 mol, 3 equiv), Pd(dppf)$Cl_2$-$CH_2Cl_2$ (0.3103 g, 0.38 mmol, 0.1 equiv) was protected with Ar, and then the 60 mL dry 1,4-dioxane was added. The mixture was heated at 110 °C (oil bath) for 20 h. After cooling down the mixture was filtered through a pad of celite. Removal of solvent under reduced pressure afforded a residue which is purified by chromatography on silica gel (PE: EA= 5:1 - 3:1) to afford the coupling product **(S)-6**.

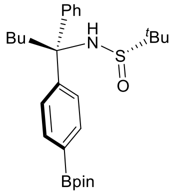


**(S)-6a**

**(*S*)-2-methyl-*N*-((*S*)-1-phenyl-1-(4-(4,4,5,5-tetramethyl-1,3,2-dioxaborolan-2-yl)phenyl)pentyl)propane-2-sulfinamide ((S)-6a)**: Purified by using a flash column chromatography (PE/EA = 3/1); isolated yield = 55%, 0.9812 g; white soild. $^1$H NMR (400 MHz, Chloroform-*d*) $\delta$ 7.77 (d, *J* = 7.6 Hz, 2H), 7.36 (d, *J* = 7.6 Hz, 2H), 7.31–7.27 (m, 4H), 7.24 (d, *J* = 7.2 Hz, 1H), 4.08 (s, 1H), 2.70–2.62 (m, 1H), 2.58–2.49 (m, 1H), 1.34 (s, 12H), 1.24 (s, 9H), 0.86 (t, *J* = 7.2 Hz, 3H). $^{13}$C NMR (101 MHz, Chloroform-*d*) $\delta$ 149.5, 144.8, 134.8, 128.2, 127.9, 127.2, 125.7, 83.8, 66.4, 56.4, 39.8, 25.7, 25.0, 24.9, 24.9, 22.9, 22.8, 13.9.

The product of **(*R*)-2-methyl-*N*-((*R*)-1-phenyl-1-(4-(4,4,5,5-tetramethyl-1,3,2-dioxaborolan-2-yl)phenyl)pentyl)propane-2-sulfinamide ((R)-6a)** was synthesized in the same way as above.

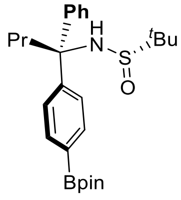


**(S)-6b**

**(*S*)-2-methyl-*N*-((*S*)-1-phenyl-1-(4-(4,4,5,5-tetramethyl-1,3,2-dioxaborolan-2-yl)phenyl)butyl)propane-2-sulfinamide ((S)-6b)**: Purified by using a flash column chromatography (PE/EA = 5/1); isolated yield = 60%, 1.0384 g; white solid. $^1$H NMR (400 MHz, Chloroform-*d*) $\delta$ 7.77 (d, *J* = 8.0 Hz, 1H), 7.39–7.33 (m, 3H), 7.31–7.26 (m, 5H), 4.09 (s, 1H), 2.68–2.60 (m, 1H), 2.55–2.47

(m, 1H), 1.34 (s, 9H), 1.32–1.27 (m, 2H), 1.24 (s, 12H), 0.94 (t, *J* = 5.6 Hz, 3H). $^{13}$C NMR (101 MHz, Chloroform-*d*) *δ* 145.3, 143.2, 133.8, 129.2, 128.8, 128.2, 127.5, 127.0, 83.8, 70.6, 56.6, 33.5, 24.8, 22.8, 18.6, 18.3.

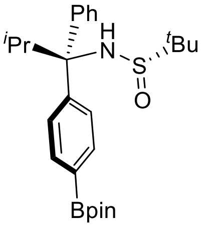


**(S)-6c**

**(S)-2-methyl-N-((S)-2-methyl-1-phenyl-1-(4-(4,4,5,5-tetramethyl-1,3,2-dioxaborolan-2-yl)phenyl)propyl)propane-2-sulfinamide ((S)-6c)**: Purified by using a flash column chromatography (PE/EA = 4/1); isolated yield = 55%, 0.9519 g; yellow liquid. $^{1}$H NMR (400 MHz, Chloroform-*d*) *δ* 7.78 (d, *J* = 8.4 Hz, 2H), 7.39 (d, *J* = 8.4 Hz, 2H), 7.34–7.27 (m, 5H), 4.01 (s, 1H), 3.25–3.17 (m, 1H), 1.37 (s, 12H), 1.17 (s, 9H), 0.98 (d, *J* = 6.8 Hz, 3H), 0.88 (d, *J* = 6.8 Hz, 3H). $^{13}$C NMR (101 MHz, Chloroform-*d*) *δ* 145.3, 143.2, 133.8, 129.9, 128.8, 127.5, 127.1, 83.8, 70.6, 56.7, 33.5, 25.0, 24.8, 22.8, 18.6, 18.3.

### 2.4 General procedure for the synthesis of substrates 12a

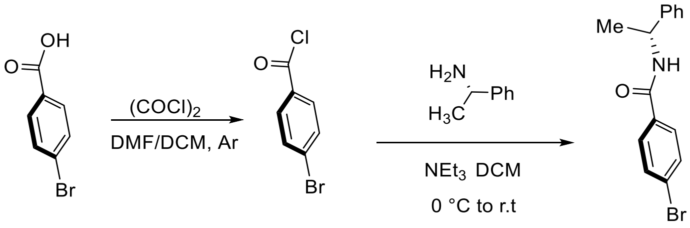


**10a** **11a** **12a**

A 50 mL round bottom flask was charged with the aryl carboxylic acid (10 mmol) and dichloromethane (40 mL) under an atmosphere of nitrogen. The solution was cooled to 0 ºC in an ice bath and oxalyl chloride (12 mmol) was added to the reaction mixture followed by three drop of DMF. The reaction was stirred at 0 ºC for one hour and then warmed to room temperature and stirred for two hours. The solution was then concentrated under reduced pressure to get the **11a** in 95% yield (2.1068 g, 9.6 mmol). To a cooled (0 °C) solution of **11a** (5 mmol) in dry DCM (30 mL) was added $Et_3N$ (15 mmol, 3 equiv) and (R)-1-phenylethan-1-amine (6 mmol, 1.2 equiv). The reaction mixture was warmed to room temperature and stirred for 2 h. Wash the mixture with aq. HCl (1 M, 15 mL), saturated aq. $NaHCO_3$ (15 mL) successively. The combined organic layers were dried by $MgSO_4$ and concentrated the resulting mixture under reduced pressure. Finally, the **12a** was obtained by recrystallizing the crude product.

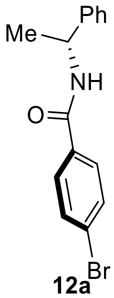


**12a**

**(*R*)-4-bromo-*N*-(1-phenylethyl)benzamide (12a)**: Employing the general procedure and purified by recrystallization as white solid; isolated yield = 88%, 1.6061 g. $^{1}$H NMR (400 MHz, Chloroform-*d*) δ 7.63 (d, *J* = 8.4 Hz, 2H), 7.51 (d, *J* = 8.4 Hz, 2H), 7.37–7.34 (m, 4H), 7.31–7.28 (m, 1H), 6.69 (d, *J* = 6.8 Hz, 1H), 5.33–5.26 (m, 1H), 1.59 (d, *J* = 6.8 Hz, 3H). $^{13}$C NMR (101 MHz, Chloroform-*d*) *δ* 165.8, 143.1, 133.5, 131.7, 128.7, 127.5, 126.2, 126.1, 49.4, 21.7.

**2.5 General procedure for the synthesis of substrates 13a**

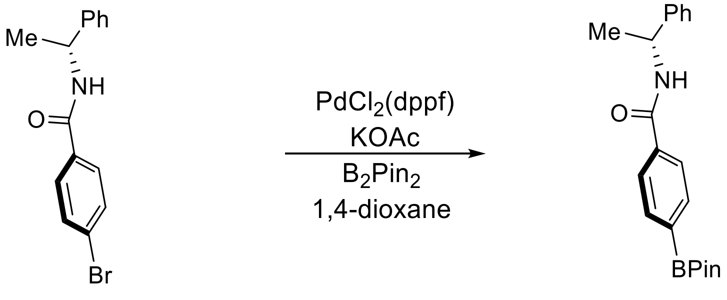


**12a** **13a**

The mixture of **12a** (3.0419 g, 10 mmol), bis(pinacolato)diboron (3.0473 g, 12 mmol), potassium acetate (1.9628 g, 20 mmol), Pd(dppf)$Cl_2$-$CH_2Cl_2$ (0.8166 g, 1 mmol) was protected with Ar, and then the 80 mL dry 1,4-dioxane was added. The mixture was heated at 110 °C (oil bath) for 20 h. After cooling down the mixture was filtered through a pad of celite. Removal of solvent under reduced pressure afforded a residue which is purified by chromatography on silica gel (PE: EA= 4:1) to afford the white product **13a**.

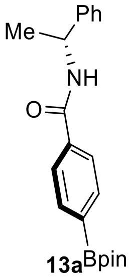


**13a**

**(*R*)-*N*-(1-phenylethyl)-4-(4,4,5,5-tetramethyl-1,3,2-dioxaborolan-2-yl)benzamide (13a)**: Purified by using a flash column chromatography (PE/EA = 6/1); isolated yield = 54%, 1.8967 g; white solid. $^{1}$H NMR (400 MHz, Chloroform-*d*) *δ* 7.86 (d, *J* = 8.4 Hz, 2H), 7.78 (d, *J* = 8.0 Hz, 2H), 7.41–7.34 (m, 4H), 7.30–7.26 (m, 1H), 6.64 (s, 1H), 5.37–5.30 (m, 1H), 1.60 (d, *J* = 6.8 Hz, 3H), 1.37 (s, 12H). $^{13}$C NMR (101 MHz, Chloroform-*d*) *δ* 166.6, 143.2, 136.9, 134.9, 128.7, 127.4, 126.3, 126.1, 84.1, 49.3, 24.9, 21.7.

### 2.6 General procedure for the synthesis of substrates 14a

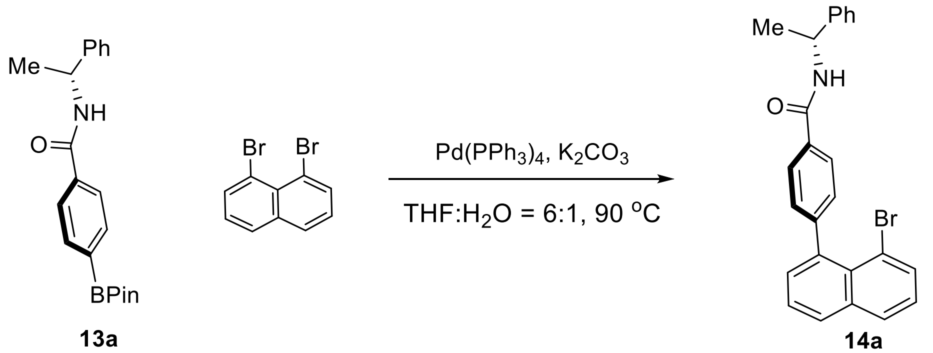


**13a** **14a**

Place 1,8-dibromonaphtalene (2.8596 g, 10 mmol), **13a** (3.5125 g, 10 mmol), $K_2CO_3$ (4.1460 g, 30 mmol) and $Pd(PPh_3)_4$ (1.1556, 1 mmol) successively to a flask under an argon atmosphere. Add THF (60 mL) and water (10 mL). Heat the mixture in an oil bath at 90 °C. Stir the mixture for 16 h. Cooled the mixture to room temperature and the liquid was separated with EA and water. The organic phase was taken and purified after concentration on silica gel (PE : EA = 5 : 1) to get the desired product **14a**.

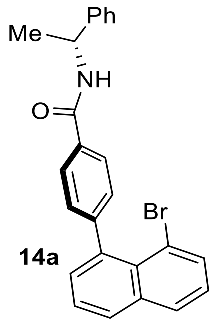

**14a**

**(*R*)-4-(8-bromonaphthalen-1-yl)-*N*-(1-phenylethyl)benzamide (14a)**: Purified by using a flash column chromatography (PE/EA = 4/1); isolated yield = 58%, 2.4960 g; yellow solid. $^1$H NMR (400 MHz, Chloroform-*d*) $\delta$ 7.92 (d, $J$ = 8.0 Hz, 2H), 7.84-7.80 (m, 3H), 7.53 (t, $J$ = 7.6 Hz, 1H), 7.47 (d, $J$ = 7.6 Hz, 2H), 7.43–7.39 (m, 5H), 7.35–7.30 (m, 2H), 6.49 (d, $J$ = 7.3 Hz, 1H), 5.46–5.39 (m, 1H), 1.68 (d, $J$ = 7.2 Hz, 3H). $^{13}$C NMR (101 MHz, Chloroform-*d*) $\delta$ 166.5, 146.5, 143.2, 139.3, 136.1, 133.8, 133.1, 130.4, 129.4, 129.3, 129.0, 128.8, 127.5, 126.4, 126.3, 126.1, 126.0, 125.3, 120.0, 49.3, 21.8.

### 2.7 General procedure for the synthesis of substrates 9

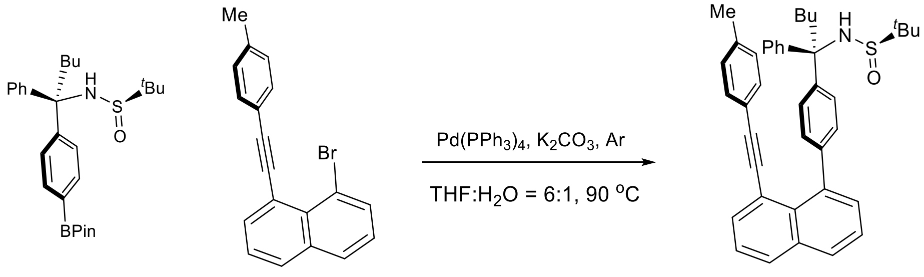


**(R)-6a** **8a** **9aa**

| entry | variation of the reaction conditions | yield (%)[b] |
|---|---|---|
| 1 | none | 67 |
| 2 | $K_3PO_4$ instead of $K_2CO_3$ | 58 |
| 3 | KOAc instead of $K_2CO_3$ | trace |
| 4 | THF instead of THF/$H_2O$ | 35 |
| 5 | $PdCl_2(PPh_3)_2$ instead of $Pd(PPh_3)_4$ | 35 |
| 6 | $PdCl_2$(dppf) instead of $Pd(PPh_3)_4$ | 45 |
| 7 | $Pd_2(dba)_3$ instead of $Pd(PPh_3)_4$ | trace |
| 8 | $Pd(OAc)_2$ instead of $Pd(PPh_3)_4$ | 56 |
| 9 | at 80 °C | 65 |
| 10 | at 100 °C | 52 |
| 11 | $Pd(PPh_3)_4$ (5 mol%) | 44 |
| 12 | $Pd(PPh_3)_4$ (20 mol%) | 62 |

[a]Reaction conditions: **(R)-6a** (0.1 mmol), **8a** (0.1 mmol), $Pd(PPh_3)_4$ (10 mol%), base (0.3 mmol) solvent 1.4 mL at 90 °C under Ar conditions. [b]Isolated yield.

To a round bottom flask, **8a** (0.1 mmol, 1 equiv), **(R)-6a** (0.1 mmol, 1 equiv), $K_2CO_3$ (41.4 mg, 0.3 mmol, 3 equiv) and $Pd(PPh_3)_4$ (12 mg, 0.01 mmol, 0.1 equiv) was added into THF/$H_2O$ (1.2 mL/0.2 mL). Heated the resulting mixture to 90 °C and stirred for 16 h. Reaction was monitored by TLC analysis and worked up for column to get pure product **9aa**.

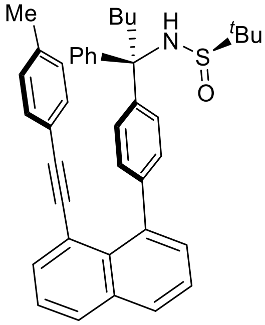


**9aa**

**(*R*)-2-methyl-*N*-((*R*)-1-phenyl-1-(4-(8-(phenylethynyl)naphthalen-1-yl)phenyl)pentyl)propane-2-sulfinamide (9aa)**: Purified by using a flash column chromatography (PE/EA = 5/1); isolated yield = 67%, 39.0 mg; clear liquid. $^1$H NMR (400 MHz, Chloroform-*d*) $\delta$ 7.89 (dd, $J$ = 16.0, 8.4 Hz, 2H), 7.78 (d, $J$ = 7.2 Hz, 1H), 7.53–7.45 (m, 3H), 7.40 (dd, $J$ = 15.2, 8.0 Hz, 3H), 7.21–7.06 (m, 10H), 3.91 (s, 1H), 2.53–2.36 (m, 2H), 2.33 (s, 3H), 1.27 (dd, $J$ = 14.4, 6.8 Hz, 2H), 1.23 (s, 9H), 1.07–0.98 (m, 1H), 0.98 - 0.86 (m, 1H), 0.83 (t, $J$ = 7.2 Hz, 3H). $^{13}$C NMR (101 MHz, Chloroform-*d*) $\delta$ 144.9, 144.4, 141.7, 140.2, 137.9, 134.5, 134.3, 140.0, 130.9, 130.5, 129.8, 129.7, 129.3, 128.8, 128.4, 128.2, 127.8, 126.8, 126.4, 125.7, 125.4, 125.2, 120.9, 120.7, 98.1, 89.8, 66.6, 56.3, 39.9, 25.7, 22.9, 22.8, 21.5, 13.9. HRMS (ESI-TOF) m/z: [M+H] $^+$ Calcd for $C_{40}H_{42}NOS$ 584.2987; Found 584.2974. $[a]_D^{25}$ = 14.588 (c = 2.55, $CHCl_3$).

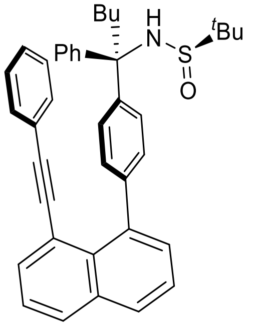


**9ba**

**(*R*)-2-methyl-*N*-((*R*)-1-phenyl-1-(4-(8-(phenylethynyl)naphthalen-1-yl)phenyl)pentyl)propane-2-sulfinamide (9ba)**: Purified by using a flash column chromatography (PE/EA = 5/1); isolated yield = 67%, 38.1 mg; clear liquid. $^1$H NMR (400 MHz, Chloroform-*d*) $\delta$ 7.92–7.75 (m, 3H), 7.54–7.36 (m, 6H), 7.28–7.08 (m, 11H), 3.90 (s, 1H), 2.66–2.25 (m, 2H), 1.28 (d, $J$ = 7.6 Hz, 4H), 1.22 (s, 9H), 0.84 (t, $J$ = 7.2 Hz, 3H). $^{13}$C NMR (101 MHz, Chloroform-*d*) $\delta$ 144.9, 144.5, 141.67,

140.1, 134.5, 131.1, 130.9, 130.5, 129.9, 129.8, 129.5, 128.4, 128.2, 128.1, 127.9(3), 127.9(8), 126.9, 126.5, 125.8, 125.5, 125.2, 123.9, 120.6, 97.9, 90.5, 66.7, 56.4, 39.9, 31.6, 25.8, 22.9(1), 22.9(6), 22.7, 14.2, 13.9. HRMS (ESI-TOF) m/z: $[M+H]^+$ Calcd for $C_{39}H_{40}NOS$ 570.2831; Found 570.2815. $[a]_D^{25}$ = 15.859 (c = 1.135, $CHCl_3$).

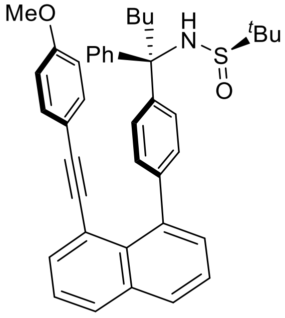

**9ca**

**(*R*)-*N*-((*R*)-1-(4-(8-((4-methoxyphenyl)ethynyl)naphthalen-1-yl)phenyl)-1-phenylpentyl)-2-methylpropane-2-sulfinamide (9ca)**: Purified by using a flash column chromatography (PE/EA = 5/1); isolated yield = 72%, 43.1 mg; clear liquid. $^1$H NMR (400 MHz, Chloroform-*d*) $\delta$ 7.89–7.83 (m, 2H), 7.75 (dd, $J$ = 7.2, 1.2 Hz, 1H), 7.51–7.35 (m, 6H), 7.19–7.07 (m, 8H), 6.77 (d, $J$ = 8.4 Hz, 2H), 3.91 (s, 1H), 3.77 (s, 3H), 2.52–2.36 (m, 2H), 1.28–1.24 (m, 2H), 1.20 (d, $J$ = 5.6 Hz, 9H), 1.09–1.01 (m, 1H), 0.92–0.85 (m, 1H), 0.82 (t, $J$ = 7.2 Hz, 3H). $^{13}$C NMR (101 MHz, Chloroform-*d*) $\delta$ 159.3, 144.9, 144.4, 141.8, 140.2, 134.6, 134.2, 132.5, 130.8, 130.5, 129.8, 129.7, 129.2, 128.4, 128.2, 127.8, 126.9, 126.4, 125.7, 125.4, 125.2, 120.9, 116.1, 113.7, 98.0, 89.2, 66.7, 56.4, 55.2, 40.0, 25.8, 22.9, 13.9. HRMS (ESI-TOF) m/z: $[M+H]^+$ Calcd for $C_{40}H_{42}NO_2S$ 600.2936; Found 600.2919. $[a]_D^{25}$ = 24.058 (c = 0.69, $CHCl_3$).

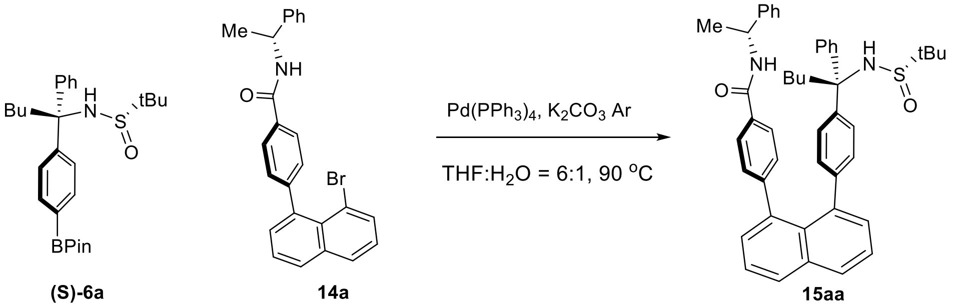


| entry | variation of the reaction conditions | yield (%)$^b$ |
|---|---|---|
| 1 | none | 75 |
| 2 | $Na_2CO_3$ instead of $K_2CO_3$ | 34 |
| 3 | KOAc instead of $K_2CO_3$ | 25 |
| 4 | $Cs_2CO_3$ instead of $K_2CO_3$ | 20 |
| 5 | dioxane/$H_2O$ instead of THF/$H_2O$ | 44 |
| 6 | THF instead of THF/$H_2O$ | 51 |
| 7 | toluene/$H_2O$ instead of THF/$H_2O$ | 37 |
| 8 | $Pd_2(dba)_3$ instead of $Pd(PPh_3)_4$ | 12 |
| 9 | $PdCl_2(dppf)$ instead of $Pd(PPh_3)_4$ | 40 |
| 10 | at 80 °C | 66 |
| 11 | at 100 °C | 71 |

$^a$Reaction conditions: **(S)-6a** (0.1 mmol), **14a** (0.1 mmol), $Pd(PPh_3)_4$ (10 mol%), base (0.3 mmol) solvent 1.4 mL at 90 °C under Ar conditions. $^b$Isolated yield.

To a dry reaction tube were added **(S)-6a** (0.1 mmol, 1 equiv), **14a** (43.0 mg, 0.1 mmol, 1equiv), $K_2CO_3$ (41.5 mg, 0.3 mmol, 3equiv), $Pd(PPh_3)_4$ (11.6 mg, 0.01 mmol, 0.1 equiv) and THF/$H_2O$ (1.2 mL/0.2 mL) under Ar atmosphere, heated the reaction to 90 °C for 16 h. Reaction was monitored by TLC analysis and worked up for column (PE: EA = 4:1) to afford the desired product **15aa**.

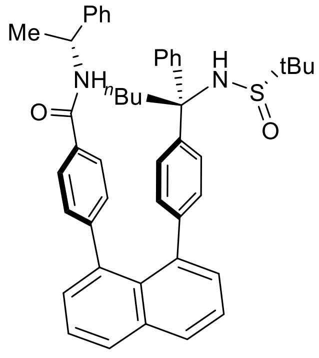

**15aa**

**4-(8-(4-((*S*)-1-(((*S*)-tert-butylsulfinyl)amino)-1-phenylpentyl)phenyl)naphthalen-1-yl)-*N*-((*R*)-1-phenylethyl)benzamide (15aa)**: Purified by using a flash column chromatography (PE/EA = 4/1); isolated yield = 75%, 52.0 mg; clear liquid. $^{1}$H NMR (400 MHz, Chloroform-*d*) $\delta$ 7.96 (t, $J$ = 7.3 Hz, 2H), 7.56 (q, $J$ = 7.4 Hz, 3H), 7.46 (d, $J$ = 7.0 Hz, 1H), 7.41 (d, $J$ = 7.6 Hz, 3H), 7.38–7.33 (m, 5H), 7.31–7.28 (m, 1H), 7.23 (t, $J$ = 7.2 Hz, 1H), 7.18 (d, $J$ = 7.8 Hz, 2H), 7.11 (d, $J$ = 8.2 Hz, 2H), 7.04–6.94 (m, 3H), 6.81 (d, $J$ = 6.9 Hz, 1H), 6.52 (d, $J$ = 7.6 Hz, 1H), 5.36 (p, $J$ = 6.7 Hz, 1H), 3.91 (s, 1H), 2.38 (t, $J$ = 7.8 Hz, 2H), 1.58 (d, $J$ = 6.9 Hz, 3H), 1.36–1.30 (m, 2H), 1.21 (s, 9H), 1.01 (m, 2H), 0.87 (t, $J$ = 7.2 Hz, 3H). $^{13}$C NMR (101 MHz, Chloroform-*d*) $\delta$ 166.1, 147.0, 145.1, 143.8, 143.4, 141.7, 139.8, 139.4, 135.5, 135.2, 131.8, 131.3, 131.2, 129.8, 129.4, 129.2, 128.9, 128.7, 128.4, 128.0, 127.9, 127.4, 127.0, 126.3, 125.4, 125.1, 66.4, 56.4, 49.2, 39.8, 26.1, 22.9, 22.0, 14.0. HRMS (ESI-TOF) m/z: [M+H] $^{+}$ Calcd for $C_{46}H_{49}N_2O_2S$ 693.3509; Found 693.3498. $[a]_D^{25}$ = -24.8 (c = 1.00, $CHCl_3$).

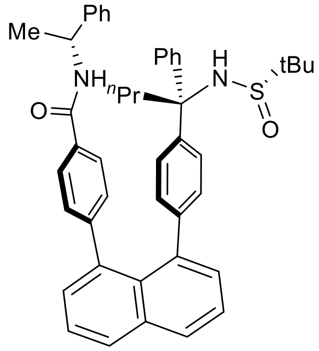

**15ab**

**4-(8-(4-((*S*)-1-(((*S*)-tert-butylsulfinyl)amino)-1-phenylbutyl)phenyl)naphthalen-1-yl)-*N*-((*R*)-1-phenylethyl)benzamide (15ab)**: Purified by using a flash column chromatography (PE/EA = 3/1); isolated yield = 55%, 37.3 mg; clear liquid. $^{1}$H NMR (400 MHz, Chloroform-*d*) $\delta$ 7.98–7.94 (m, 2H), 7.56 (dt, $J$ = 8.1, 3.6 Hz, 3H), 7.47 – 7.45 (m, 1H), 7.42–7.38 (m, 3H), 7.36–7.29 (m, 6H), 7.22 (t, $J$ = 7.3 Hz, 1H), 7.19–7.16 (m, 2H), 7.11 (d, $J$ = 8.6 Hz, 2H), 7.05 (s, 2H), 6.95 (d, $J$ = 7.8 Hz, 1H), 6.77 (d, $J$ = 7.5 Hz, 1H), 6.48 (d, $J$ = 7.7 Hz, 1H), 5.38–5.31 (m, 1H), 3.92 (s, 1H), 2.40–2.30 (m, 2H), 1.58 (d, $J$ = 6.9 Hz, 3H), 1.21 (s, 9H), 1.10 (m, 1H), 0.99–0.94 (m, 1H), 0.91 (d, $J$ = 5.7 Hz, 3H). $^{13}$C NMR (101 MHz, Chloroform-*d*) $\delta$ 166.1, 147.0, 145.1, 143.7, 143.3, 141.7, 139.8, 139.4, 135.5, 131.7, 131.3, 131.2, 129.8, 129.5, 129.4, 129.1, 128.9, 128.7, 128.4, 128.0, 127.9, 127.4, 127.0, 126.3, 125.4, 125.1, 66.4, 56.4, 49.2, 42.3, 22.9, 21.9, 17.4, 14.3. HRMS (ESI-TOF) m/z: [M+H] $^{+}$ Calcd for $C_{45}H_{47}N_2O_2S$ 679.3353; Found 679.3331. $[a]_D^{25}$ = -4.2 (c = 0.33, $CHCl_3$).

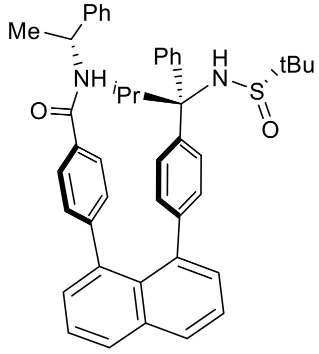

**15ac**

**4-(8-(4-((*S*)-1-(((*S*)-tert-butylsulfinyl)amino)-2-methyl-1-phenylpropyl)phenyl)naphthalen-1-yl)-*N*-((*R*)-1-phenylethyl)benzamide (15ac)**: Purified by using a flash column chromatography (PE/EA = 4/1); isolated yield = 55%, 37.3 mg; clear liquid. $^1$H NMR (400 MHz, Chloroform-*d*) $\delta$ 7.99 (d, *J* = 8.2 Hz, 2H), 7.71 (d, *J* = 7.4 Hz, 1H), 7.59 (q, *J* = 7.7 Hz, 2H), 7.52 – 7.43 (m, 5H), 7.37 (m, 4H), 7.28–7.26 (m, 1H), 7.22–7.11 (m, 8H), 6.98 (d, *J* = 8.2 Hz, 1H), 6.86 (d, *J* = 8.2 Hz, 1H), 5.30 (m, 1H), 4.57 (s, 1H), 2.82 (p, *J* = 6.6 Hz, 1H), 1.28 (d, *J* = 7.2 Hz, 3H), 1.17 (s, 9H), 0.78 (d, *J* = 6.8 Hz, 3H), 0.62 (d, *J* = 6.5 Hz, 3H). $^{13}$C NMR (101 MHz, Chloroform-*d*) δ 167.2, 146.6, 144.1, 142.0, 139.9, 139.6, 138.3, 135.5, 132.7, 131.5, 131.1, 129.8, 129.0, 128.9, 128.6, 128.5, 128.4, 128.0, 127.9, 127.8, 126.8, 126.2, 126.1, 125.3, 70.4, 56.1, 48.7, 36.6, 23.1, 22.0, 18.6, 17.5. HRMS (ESI-TOF) m/z: [M+H] $^+$ Calcd for $C_{45}H_{47}N_2O_2S$ 679.3353; Found 679.3328. $[a]_D^{25}$ = -16.0 (c = 0.40, $CHCl_3$).

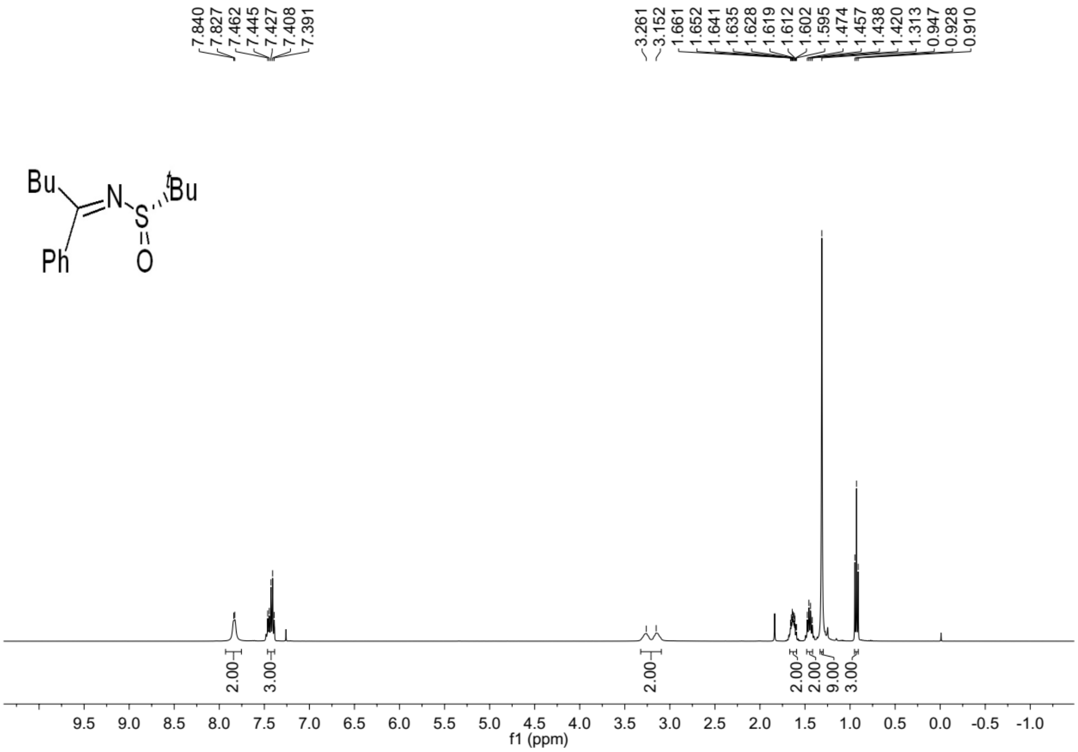
7.840
7.827
7.462
7.445
7.427
7.408
7.391
3.261
3.152
1.661
1.652
1.641
1.635
1.628
1.619
1.612
1.602
1.595
1.474
1.457
1.438
1.420
1.313
0.947
0.928
0.910
Bu
N
S
tBu
O
Ph
2.00
3.00
2.00
2.00
2.00
9.00
3.00
9.5 9.0 8.5 8.0 7.5 7.0 6.5 6.0 5.5 5.0 4.5 4.0 3.5 3.0 2.5 2.0 1.5 1.0 0.5 0.0 -0.5 -1.0
f1 (ppm)

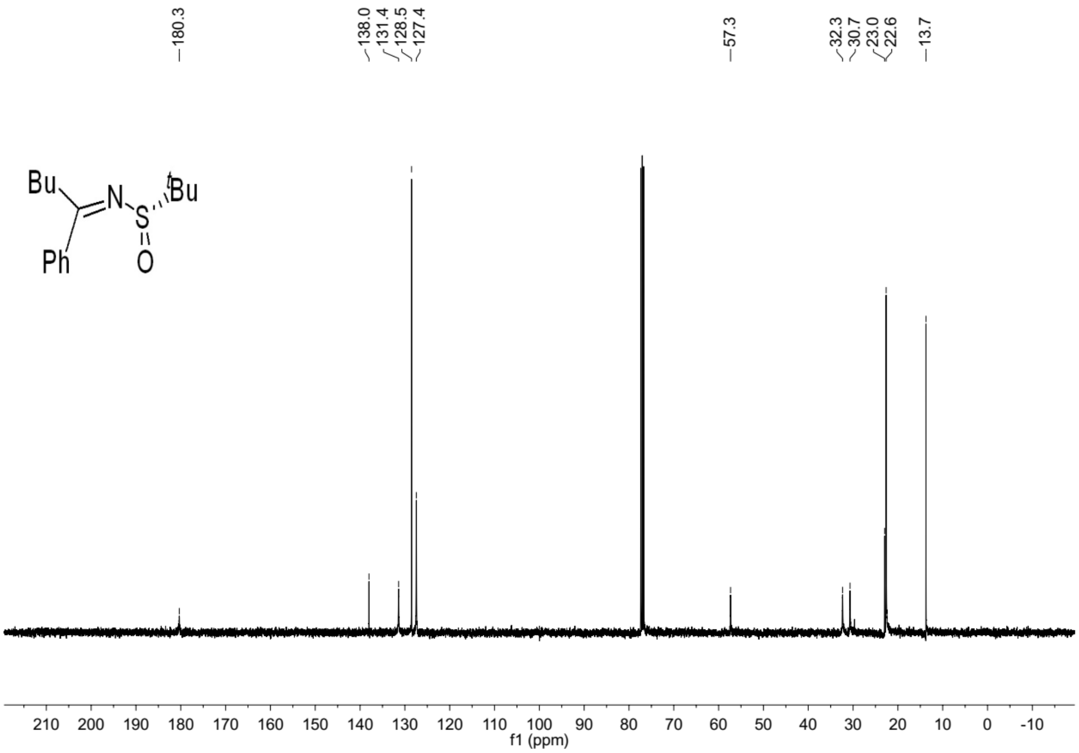
180.3
138.0
131.4
128.5
127.4
57.3
32.3
30.7
23.0
22.6
13.7
Bu
N
S
tBu
O
Ph
210 200 190 180 170 160 150 140 130 120 110 100 90 80 70 60 50 40 30 20 10 0 -10
f1 (ppm)

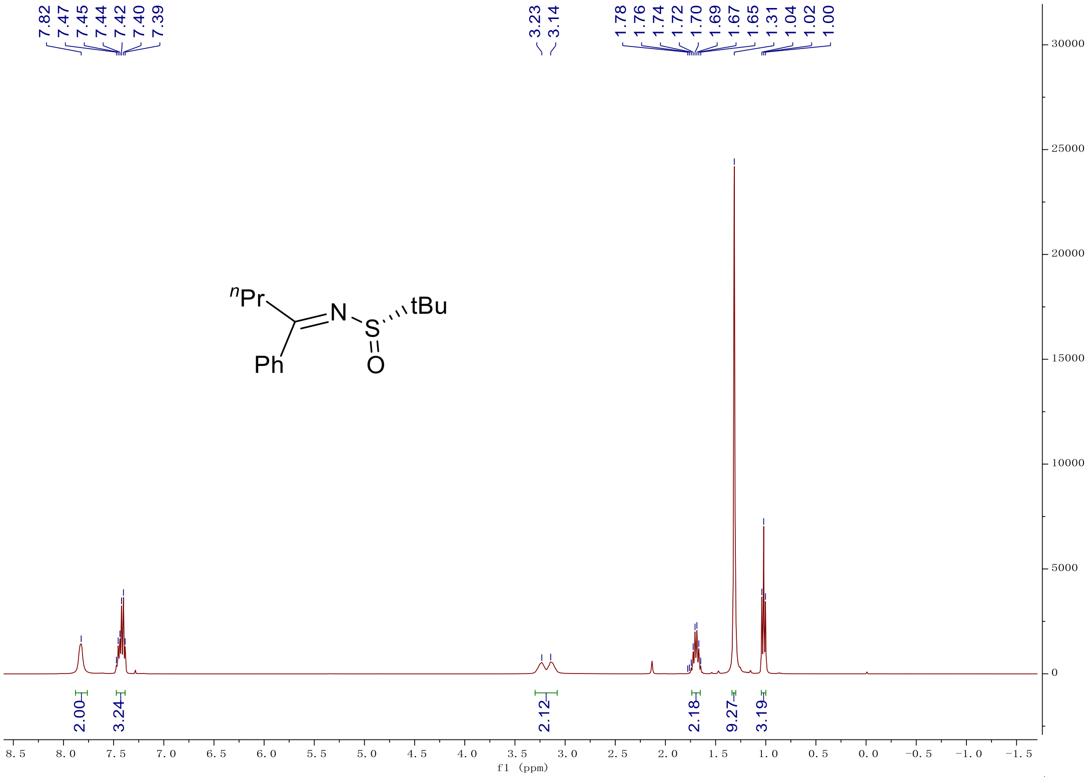
7.82
7.47
7.45
7.44
7.42
7.40
7.39
3.23
3.14
1.78
1.76
1.74
1.72
1.70
1.69
1.67
1.65
1.31
1.04
1.02
1.00
nPr
N
S
tBu
Ph
O
2.00
3.24
2.12
2.18
9.27
3.19
f1 (ppm)

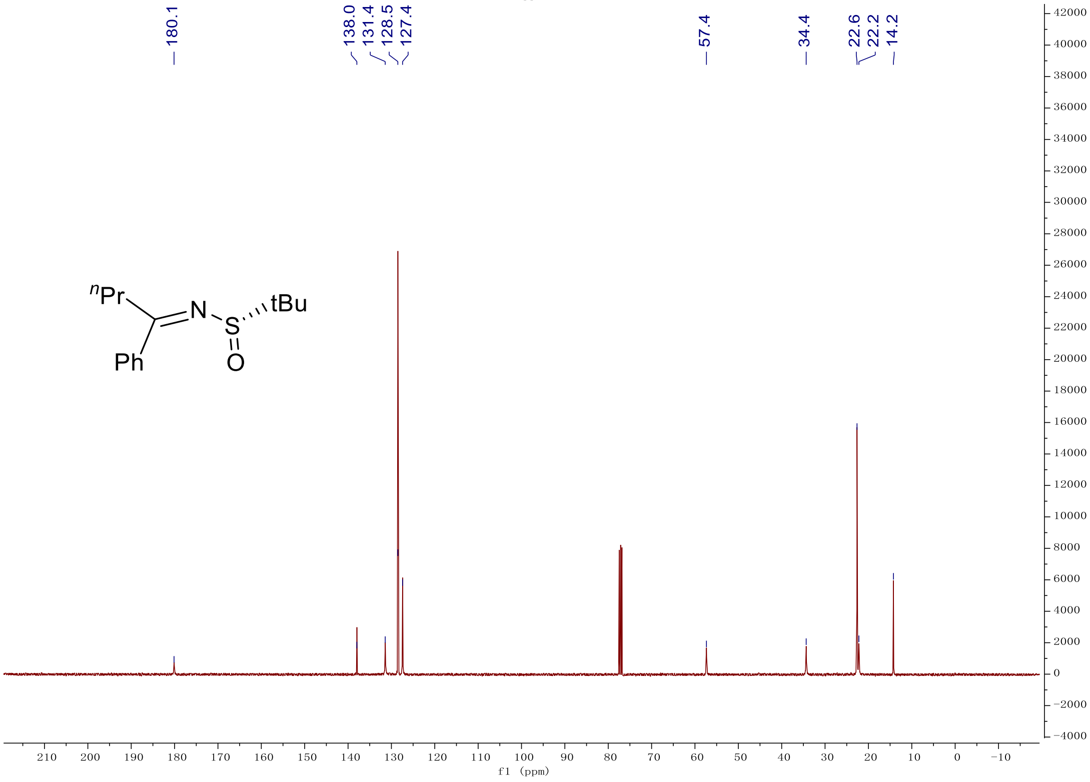
180.1
138.0
131.4
128.5
127.4
57.4
34.4
22.6
22.2
14.2
nPr
N
S
tBu
Ph
O
f1 (ppm)

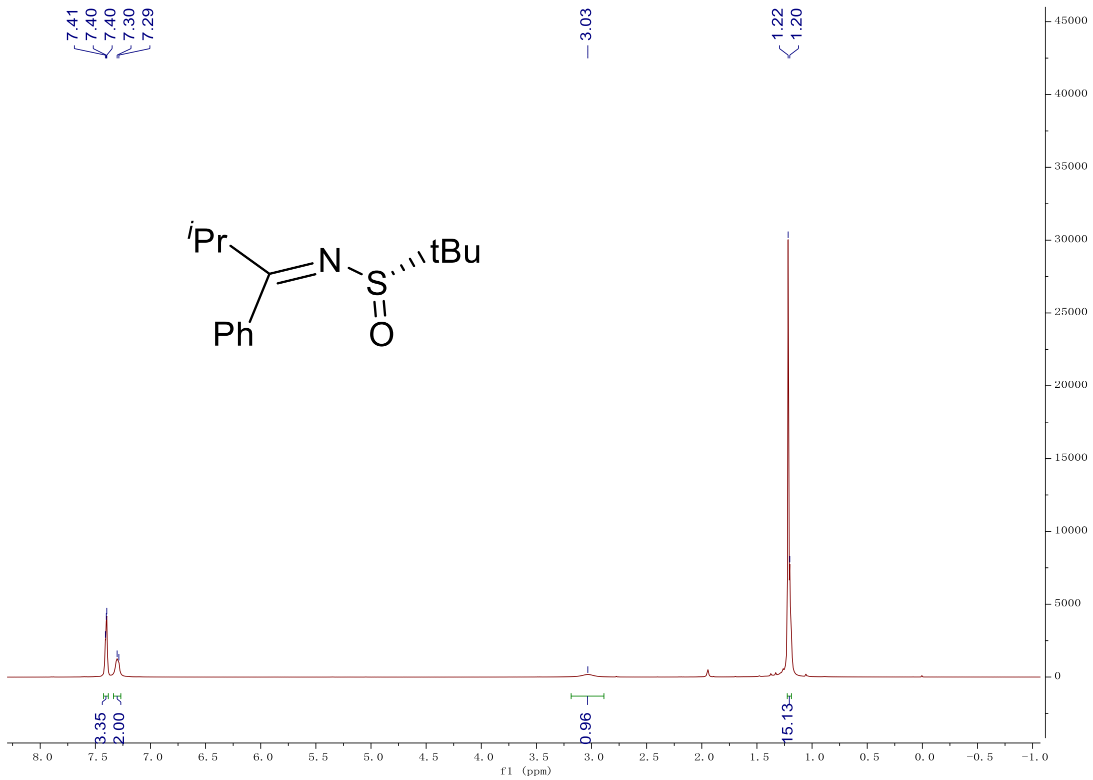
7.41
7.40
7.40
7.30
7.29
3.03
1.22
1.20
iPr
N
S
tBu
O
Ph
3.35
2.00
0.96
15.13
f1 (ppm)

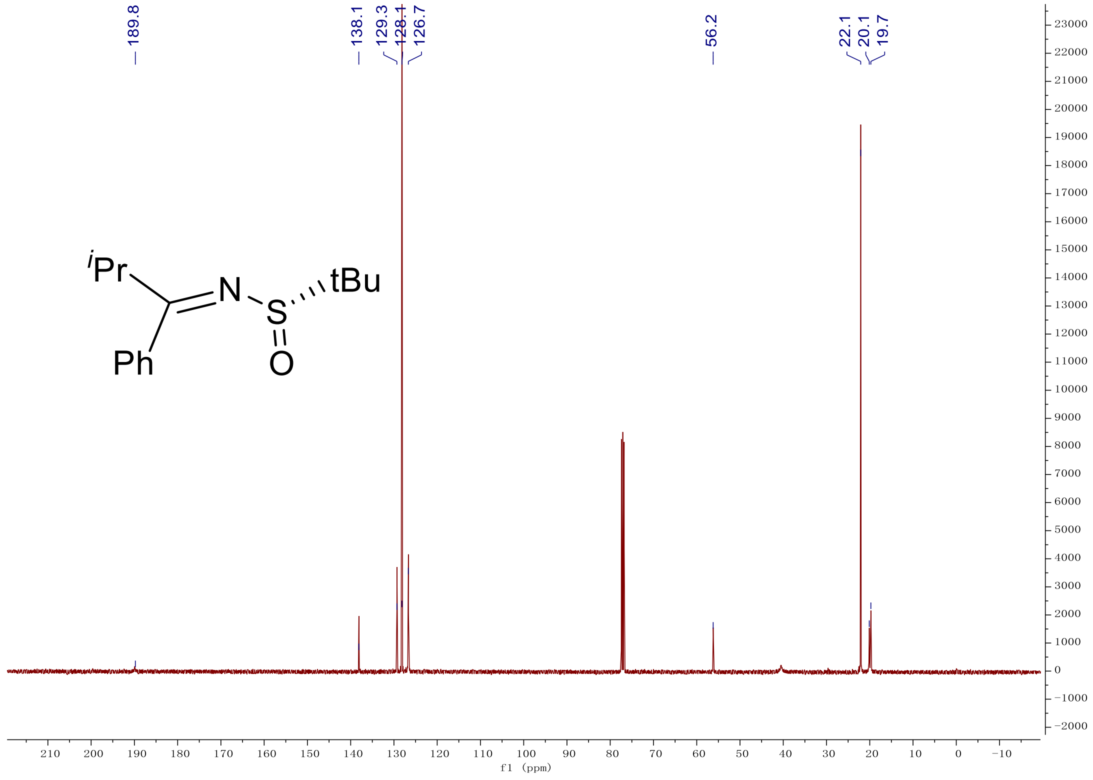
189.8
138.1
129.3
128.4
126.7
56.2
22.1
20.1
19.7
iPr
N
S
tBu
O
Ph
f1 (ppm)

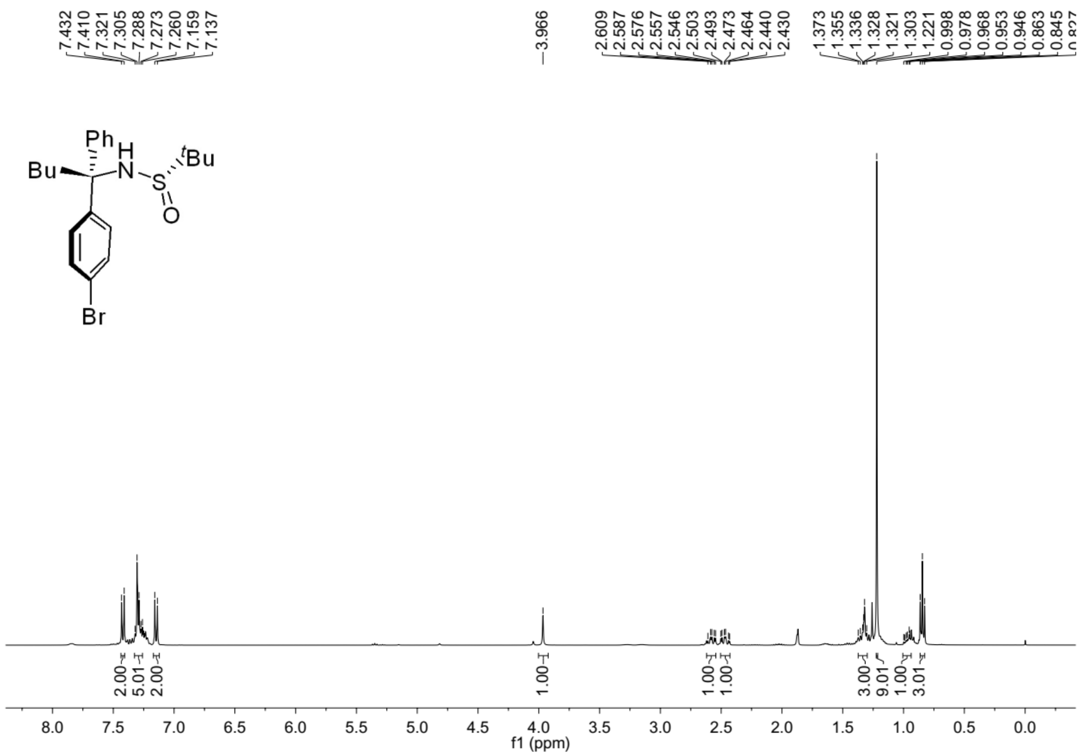
7.432
7.410
7.321
7.305
7.288
7.273
7.260
7.159
7.137
3.966
2.609
2.587
2.576
2.557
2.546
2.503
2.493
2.473
2.464
2.440
2.430
1.373
1.355
1.336
1.328
1.321
1.303
1.221
0.998
0.978
0.968
0.953
0.946
0.863
0.845
Ph
H
Bu
N
S
tBu
O
Br
2.00
5.01
2.00
1.00
1.00
1.00
3.00
9.01
1.00
3.01
8.0 7.5 7.0 6.5 6.0 5.5 5.0 4.5 4.0 3.5 3.0 2.5 2.0 1.5 1.0 0.5 0.0
f1 (ppm)

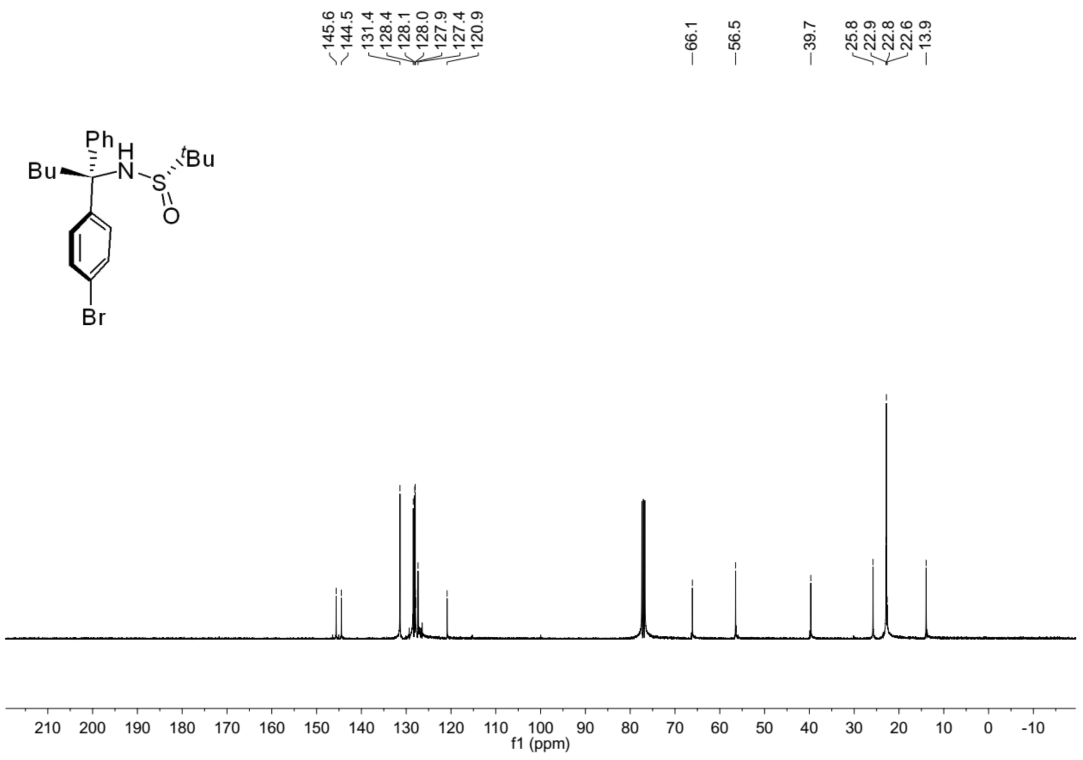
145.6
144.5
131.4
128.4
128.1
128.0
127.9
127.4
120.9
66.1
56.5
39.7
25.8
22.9
22.8
22.6
13.9
Ph
H
Bu
N
S
tBu
O
Br
210 200 190 180 170 160 150 140 130 120 110 100 90 80 70 60 50 40 30 20 10 0 -10
f1 (ppm)

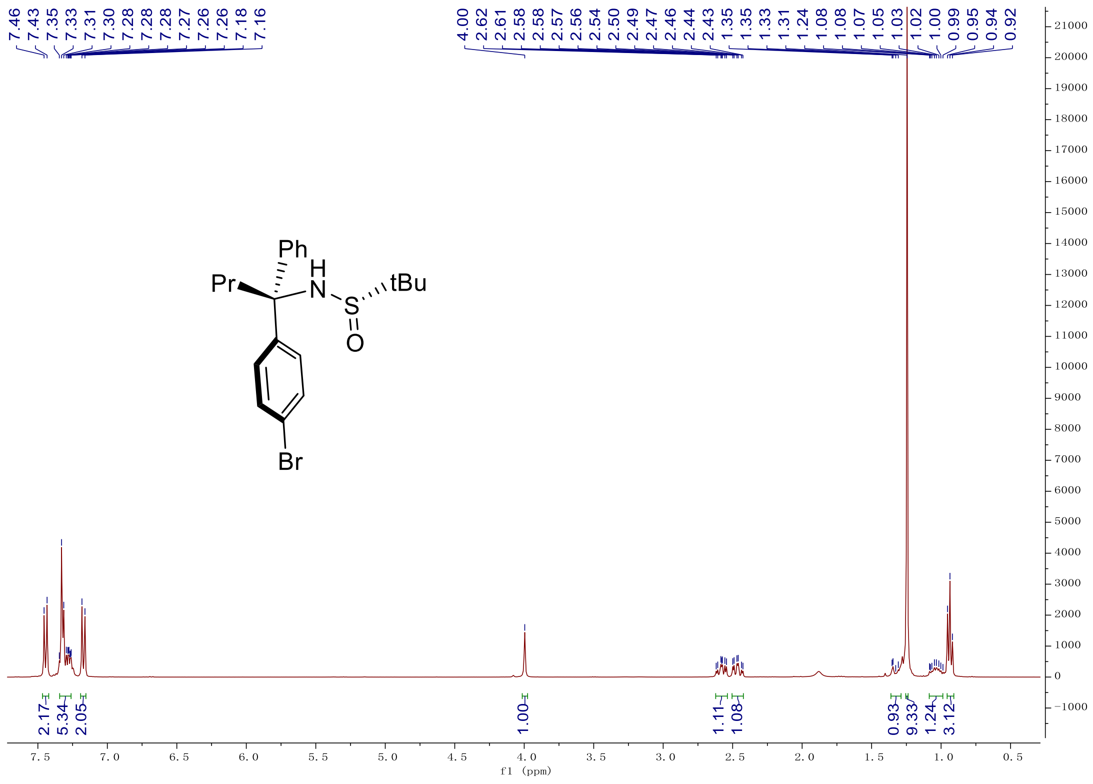

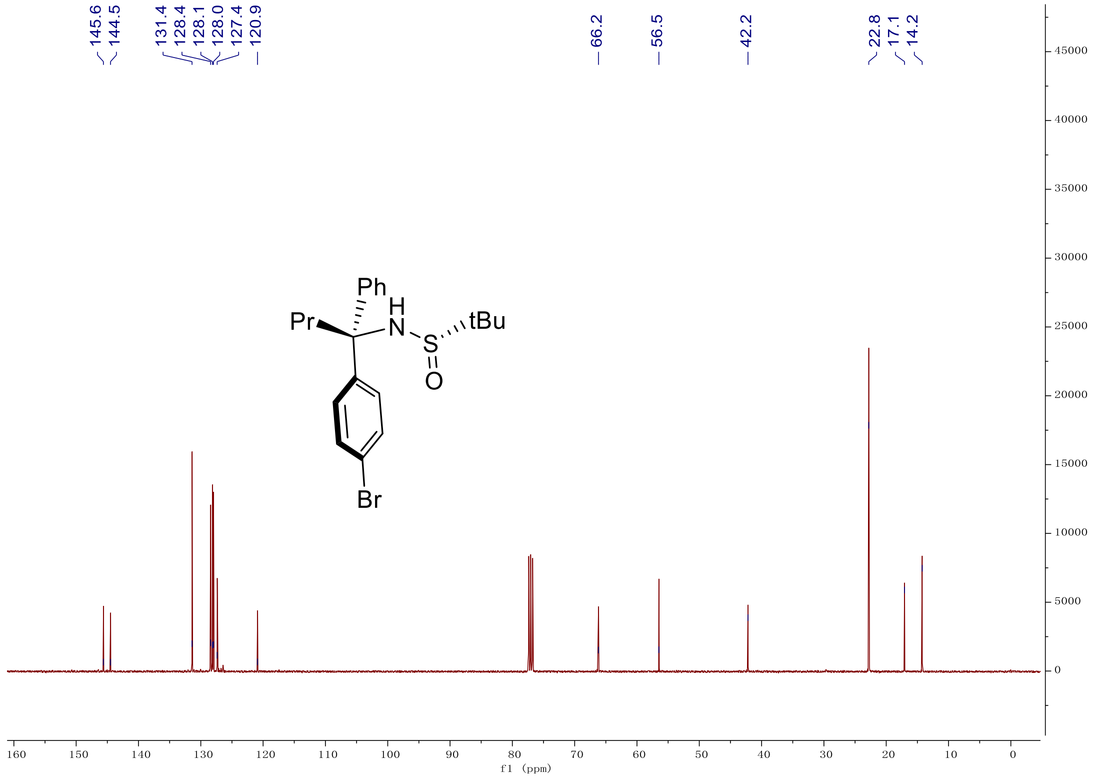

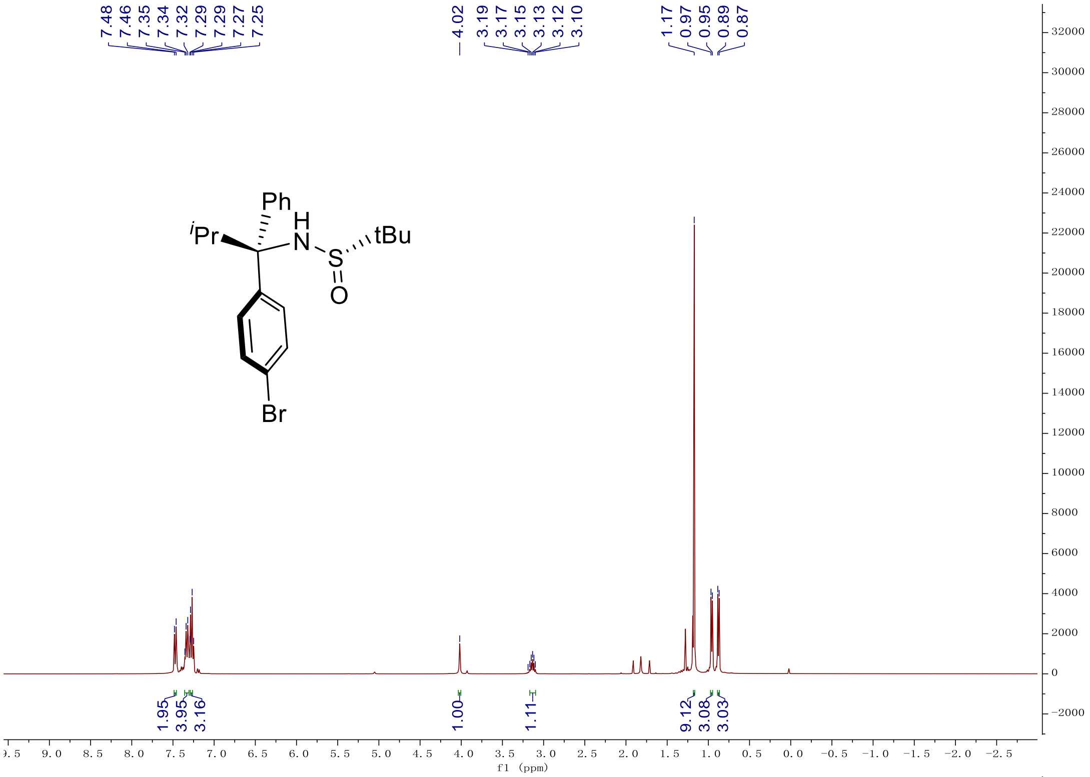
7.48 7.46 7.35 7.34 7.32 7.29 7.29 7.27 7.25
4.02 3.19 3.17 3.15 3.13 3.12 3.10
1.17 0.97 0.95 0.89 0.87
Ph
H
$^i$Pr
N
S
tBu
O
Br
1.95 3.95 3.16
1.00
1.11
9.12 3.08 3.03
f1 (ppm)

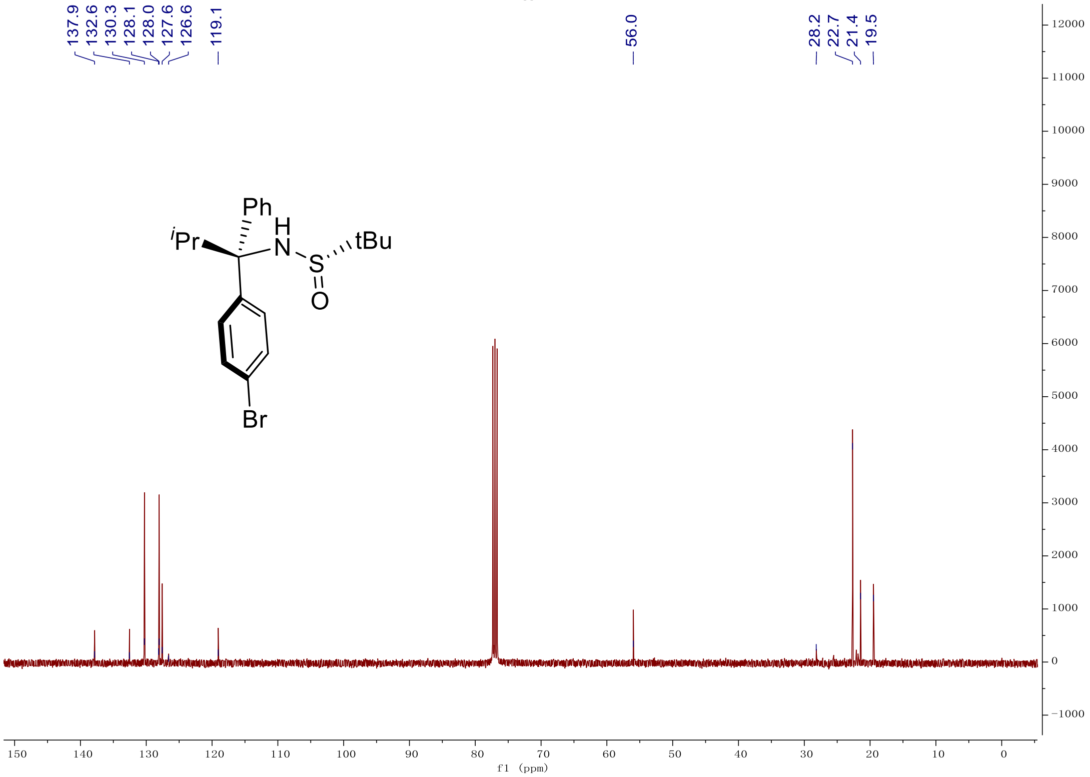
137.9 132.6 130.3 128.1 128.0 127.6 126.6 119.1
56.0
28.2 22.7 21.4 19.5
Ph
H
$^i$Pr
N
S
tBu
O
Br
f1 (ppm)

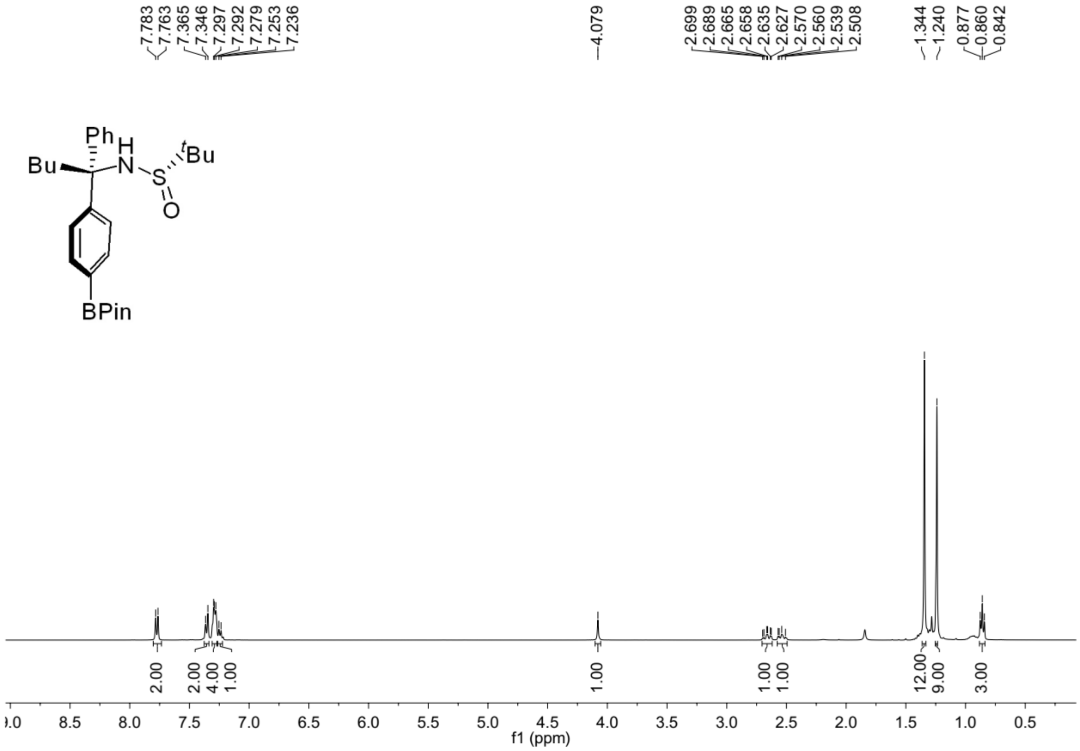

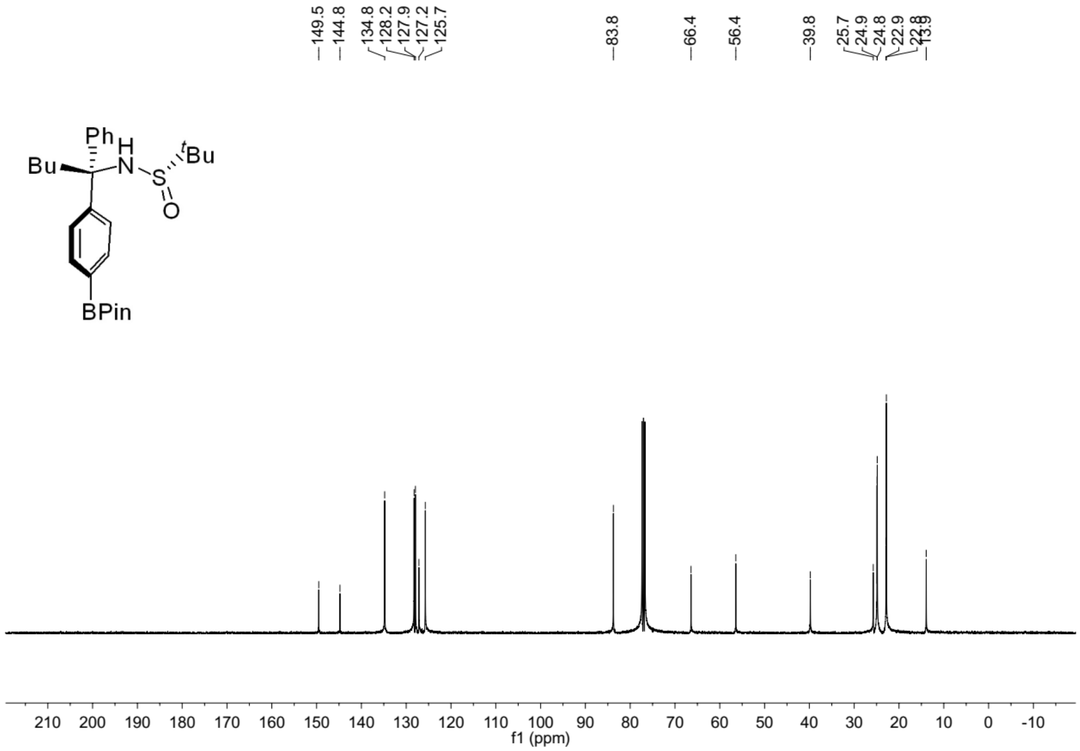

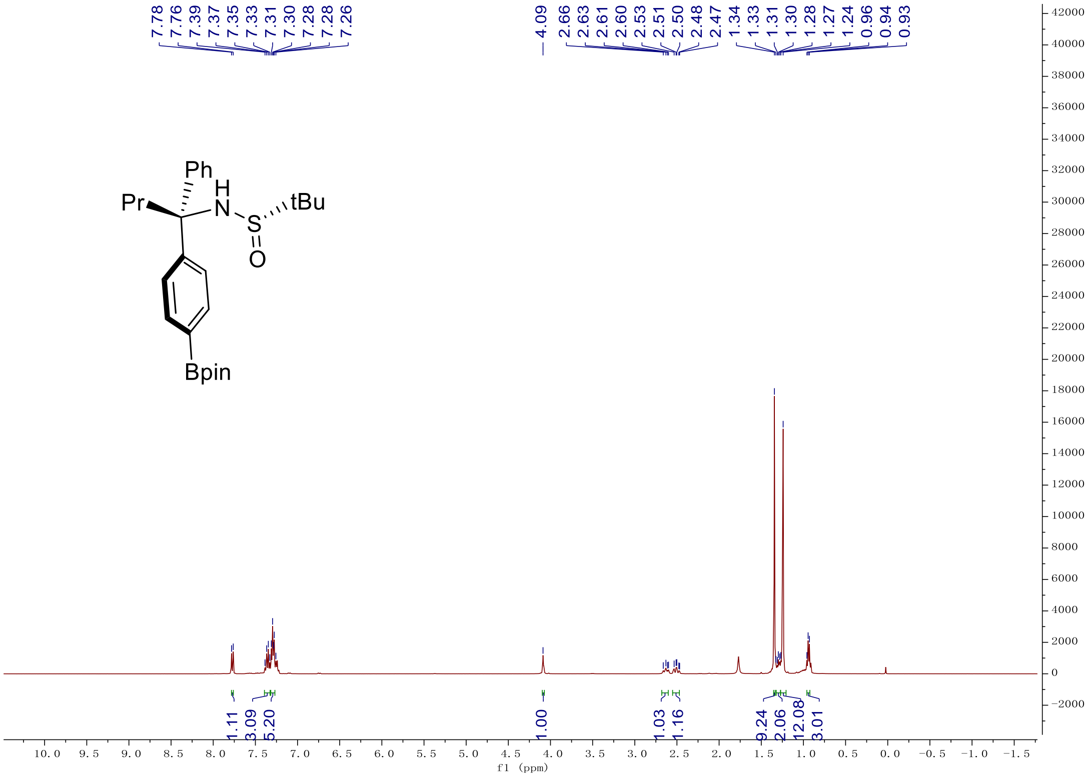
7.78
7.76
7.39
7.37
7.35
7.33
7.31
7.30
7.28
7.26
4.09
2.66
2.63
2.61
2.60
2.53
2.51
2.50
2.48
2.47
1.34
1.33
1.31
1.30
1.28
1.27
1.24
0.96
0.94
0.93
Ph
Pr
H
N
S
tBu
O
Bpin
1.11
3.09
5.20
1.00
1.03
1.16
9.24
2.06
12.08
3.01
f1 (ppm)

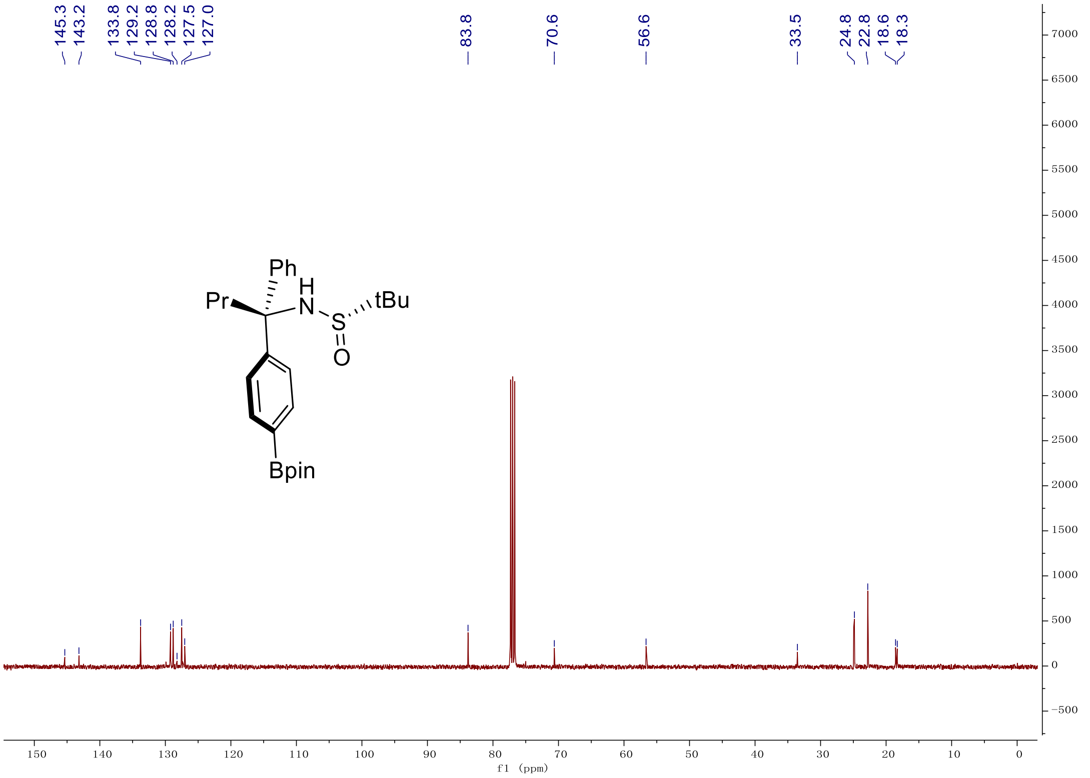
145.3
143.2
133.8
129.2
128.8
128.2
127.5
127.0
83.8
70.6
56.6
33.5
24.8
22.8
18.6
18.3
Ph
Pr
H
N
S
tBu
O
Bpin
f1 (ppm)

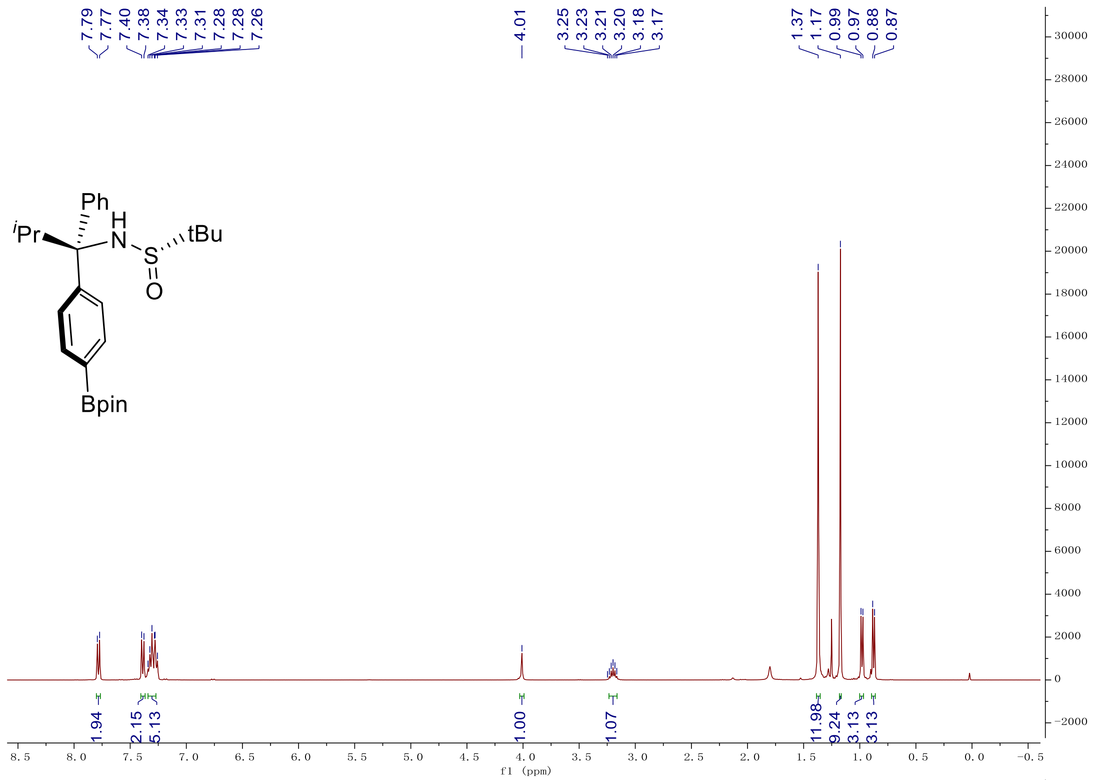
7.79
7.77
7.40
7.38
7.34
7.33
7.31
7.28
7.28
7.26
4.01
3.25
3.23
3.21
3.20
3.18
3.17
1.37
1.17
0.99
0.97
0.88
0.87
Ph
H
iPr
N
S
tBu
O
Bpin
1.94
2.15
5.13
1.00
1.07
11.98
9.24
3.13
3.13
f1 (ppm)

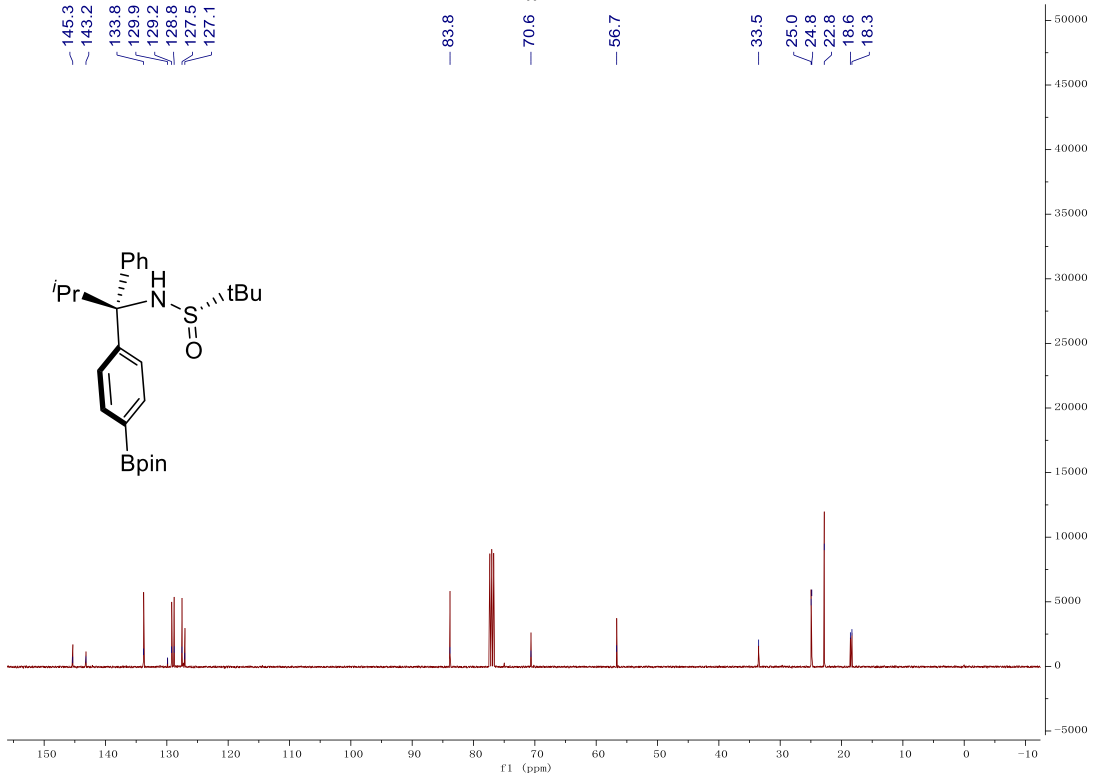
145.3
143.2
133.8
129.9
129.2
128.8
127.5
127.1
83.8
70.6
56.7
33.5
25.0
24.8
22.8
18.6
18.3
Ph
H
iPr
N
S
tBu
O
Bpin
f1 (ppm)

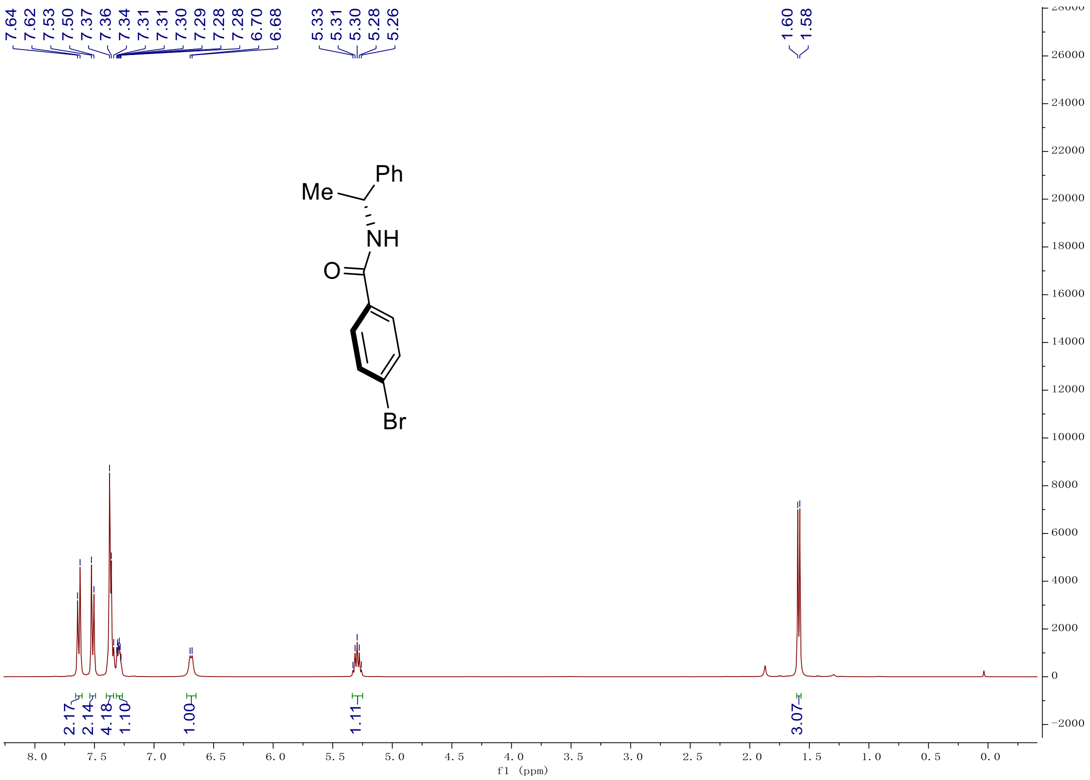
7.64
7.62
7.53
7.50
7.37
7.36
7.34
7.31
7.31
7.30
7.29
7.28
7.28
6.70
6.68
5.33
5.31
5.30
5.28
5.26
1.60
1.58
Ph
Me
NH
O
Br
2.17
2.14
4.18
1.10
1.00
1.11
3.07
f1 (ppm)

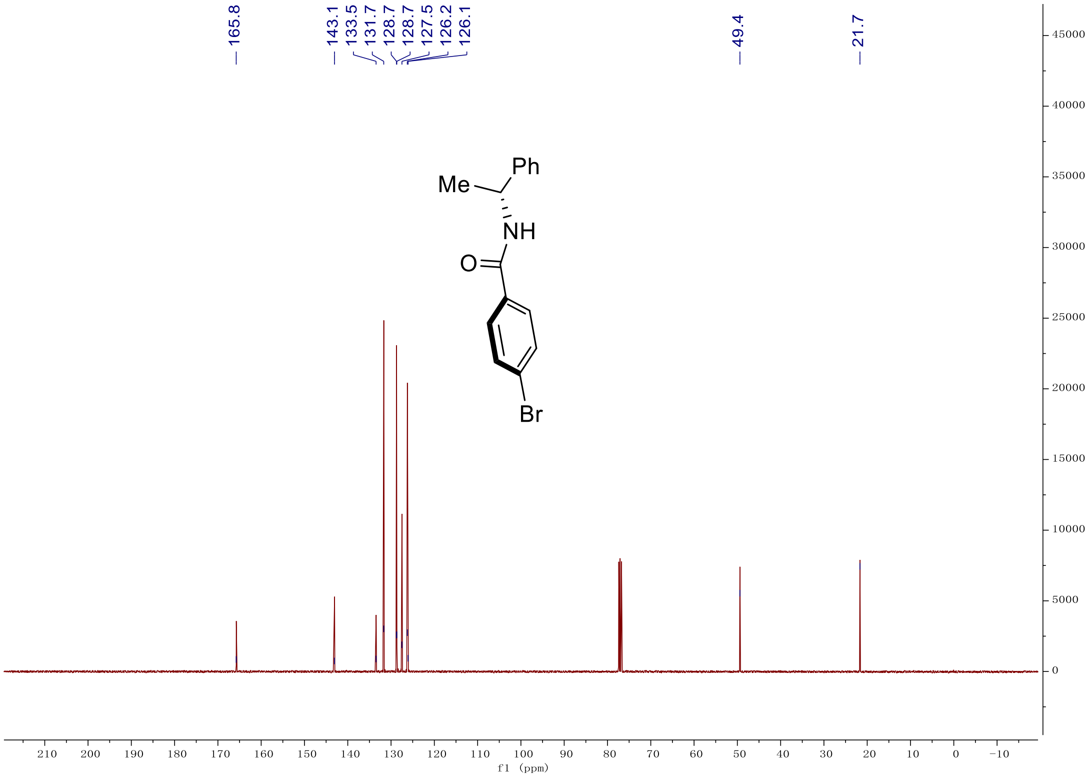
165.8
143.1
133.5
131.7
128.7
128.7
127.5
126.2
126.1
49.4
21.7
Ph
Me
NH
O
Br
f1 (ppm)

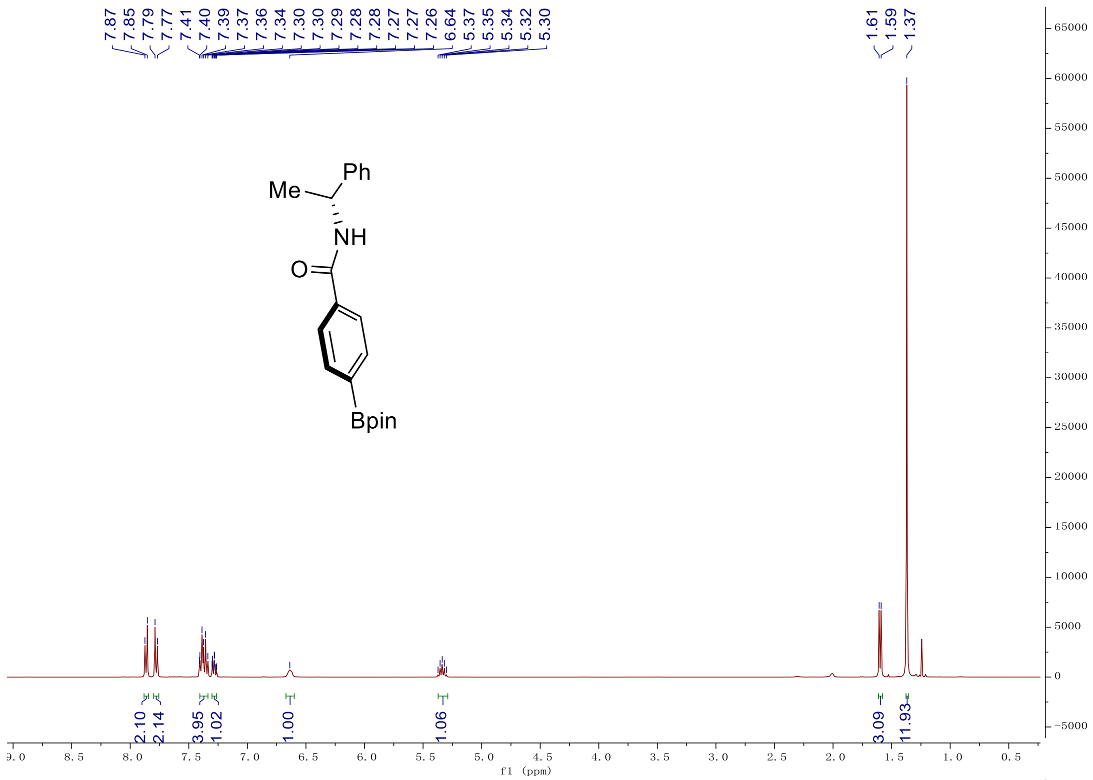
7.87 7.85 7.79 7.77 7.41 7.40 7.39 7.37 7.36 7.34 7.30 7.30 7.29 7.28 7.28 7.27 7.27 7.26 6.64 5.37 5.35 5.34 5.32 5.30
1.61 1.59 1.37
Me
Ph
NH
O
Bpin
2.10 2.14 3.95 1.02 1.00 1.06 3.09 11.93
f1 (ppm)

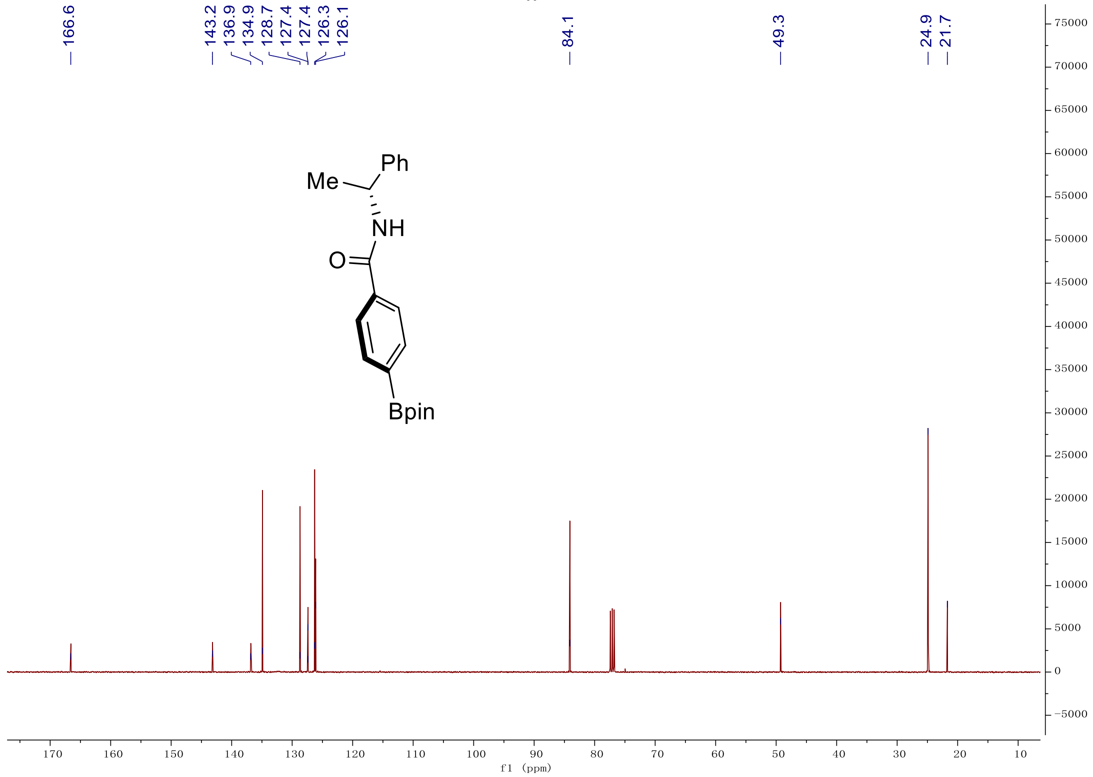
166.6
143.2 136.9 134.9 128.7 127.4 127.4 126.3 126.1
84.1
49.3
24.9 21.7
Me
Ph
NH
O
Bpin
f1 (ppm)

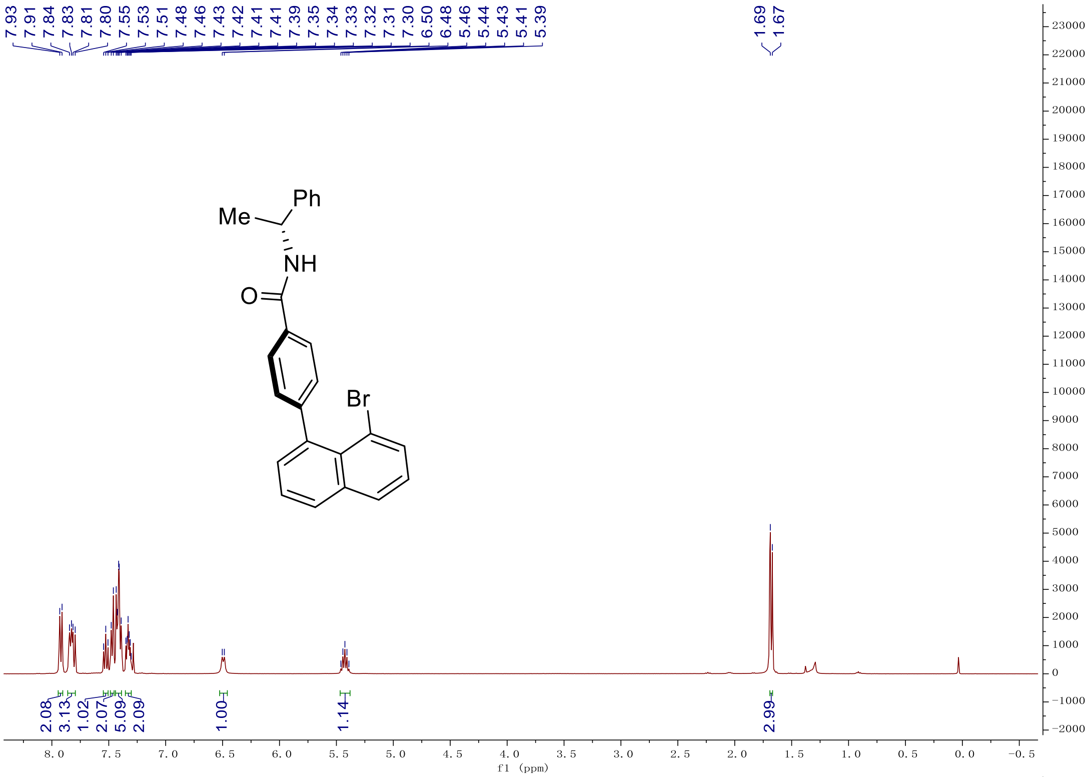
7.93
7.91
7.84
7.83
7.81
7.80
7.55
7.53
7.51
7.48
7.46
7.43
7.42
7.41
7.41
7.39
7.35
7.34
7.33
7.32
7.31
7.30
6.50
6.48
5.46
5.44
5.43
5.41
5.39
1.69
1.67
Me
Ph
NH
O
Br
2.08
3.13
1.02
2.07
5.09
2.09
1.00
1.14
2.99
f1 (ppm)

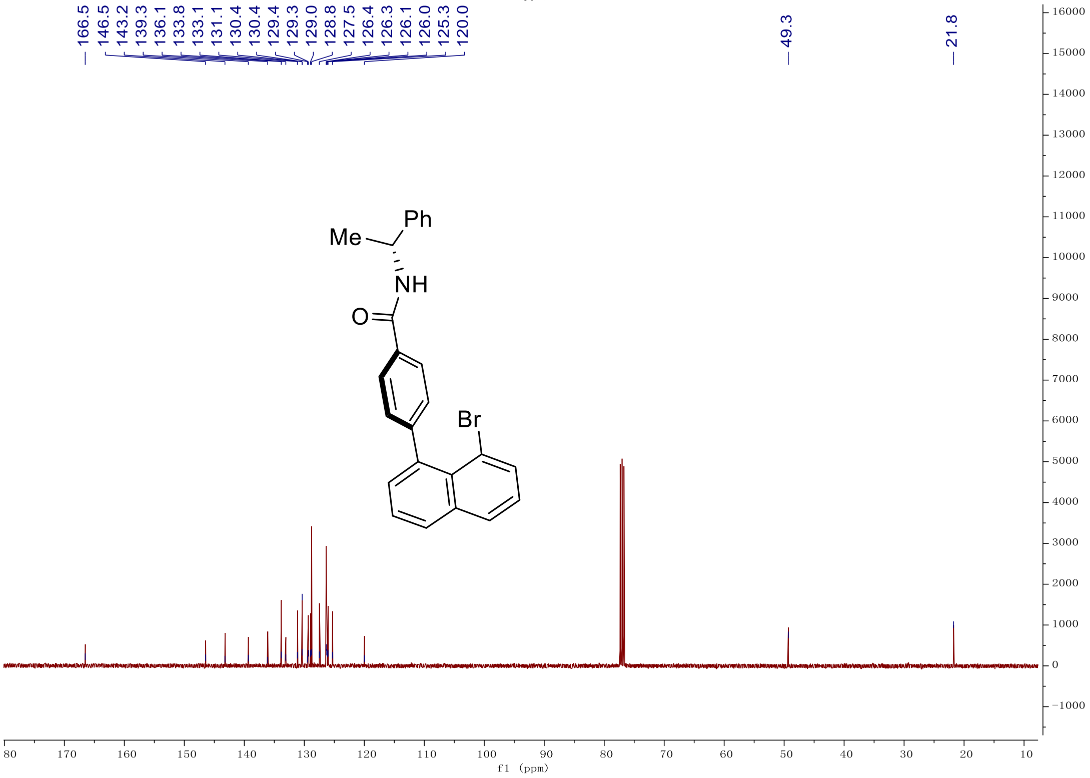
166.5
146.5
143.2
139.3
136.1
133.8
133.1
131.1
130.4
130.4
129.4
129.3
129.0
128.8
127.5
126.4
126.3
126.1
126.0
125.3
120.0
49.3
21.8
Me
Ph
NH
O
Br
f1 (ppm)

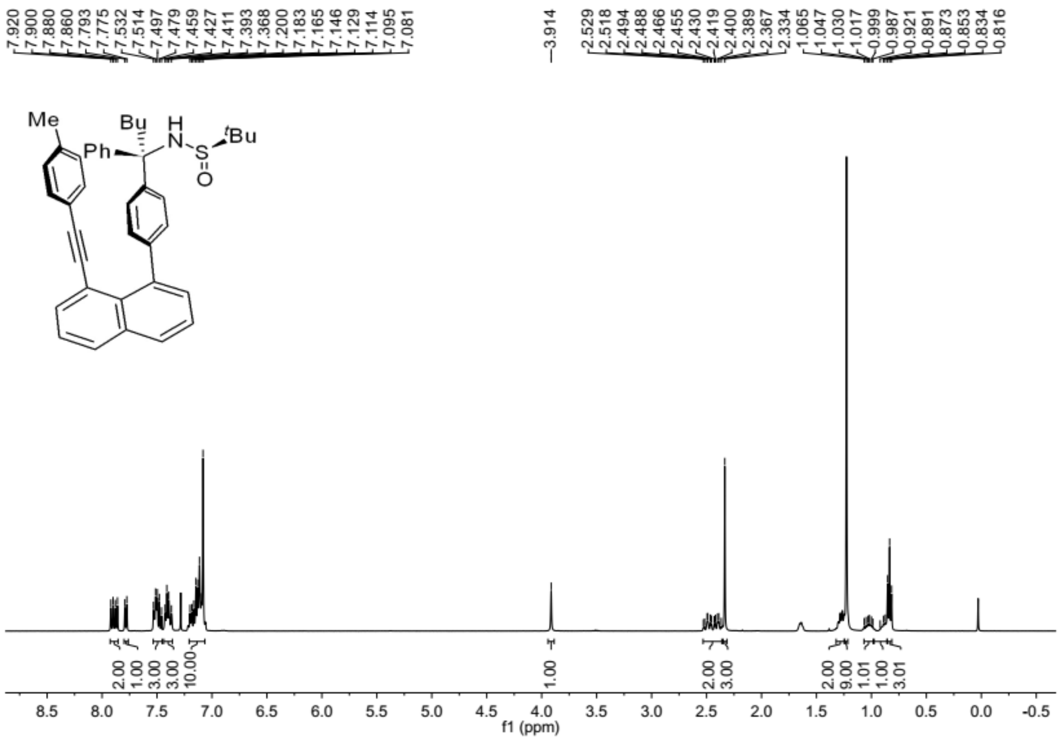
7.920
7.900
7.880
7.860
7.793
7.775
7.532
7.514
7.497
7.479
7.459
7.427
7.411
7.393
7.368
7.200
7.183
7.165
7.146
7.129
7.114
7.095
7.081
3.914
2.529
2.518
2.494
2.488
2.466
2.455
2.430
2.419
2.400
2.389
2.367
2.334
1.065
1.047
1.030
1.017
0.999
0.987
0.921
0.891
0.873
0.853
0.834
0.816
Me
Bu
H
N
Ph
S
tBu
O
2.00
1.00
3.00
3.00
10.00
1.00
2.00
3.00
2.00
9.00
1.01
1.00
3.01
f1 (ppm)

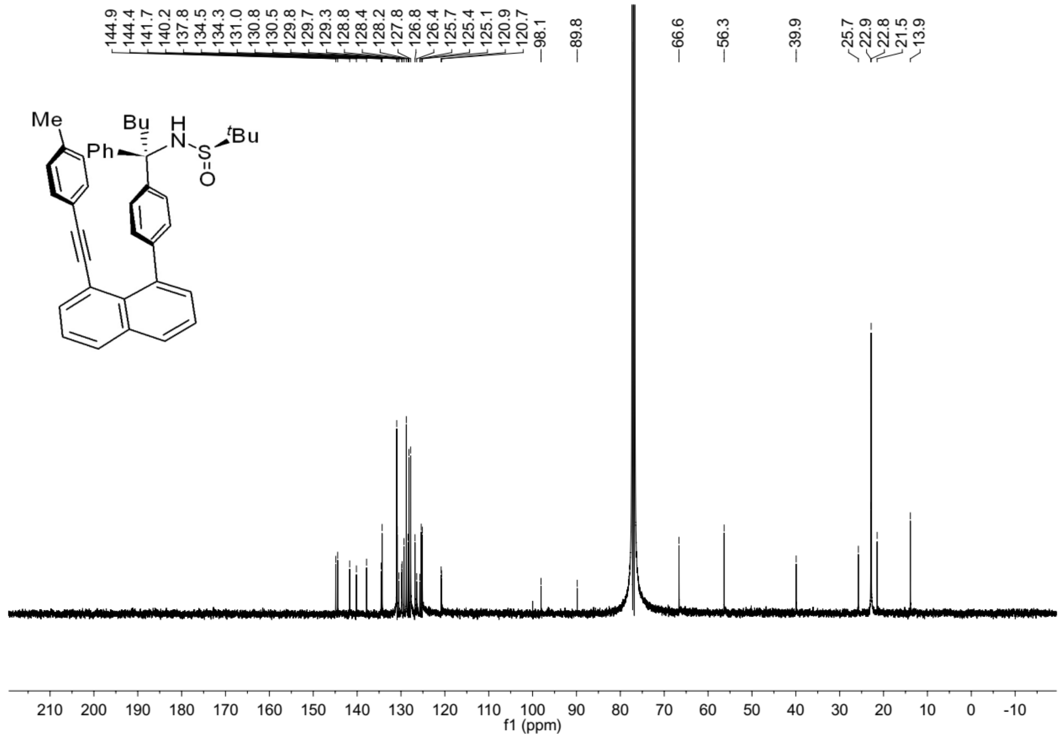
144.9
144.4
141.7
140.2
137.8
134.5
134.3
131.0
130.8
130.5
129.8
129.7
129.3
128.8
128.4
128.2
127.8
126.8
126.4
125.7
125.4
125.1
120.9
120.7
98.1
89.8
66.6
56.3
39.9
25.7
22.9
22.8
21.5
13.9
Me
Bu
H
N
Ph
S
tBu
O
f1 (ppm)

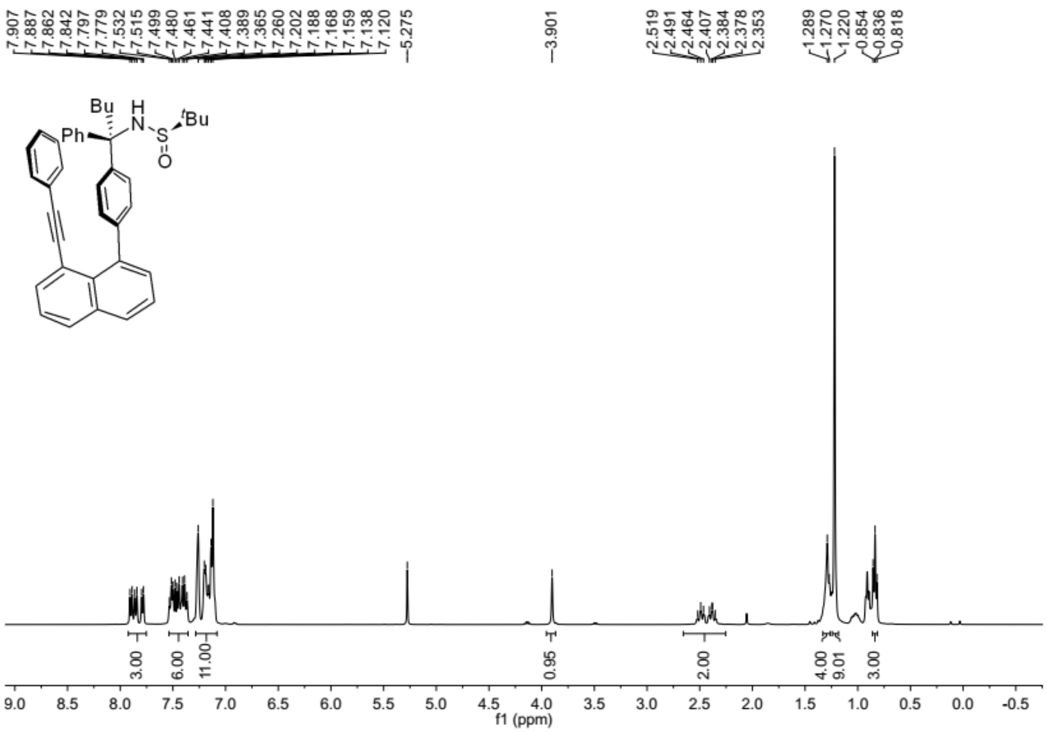

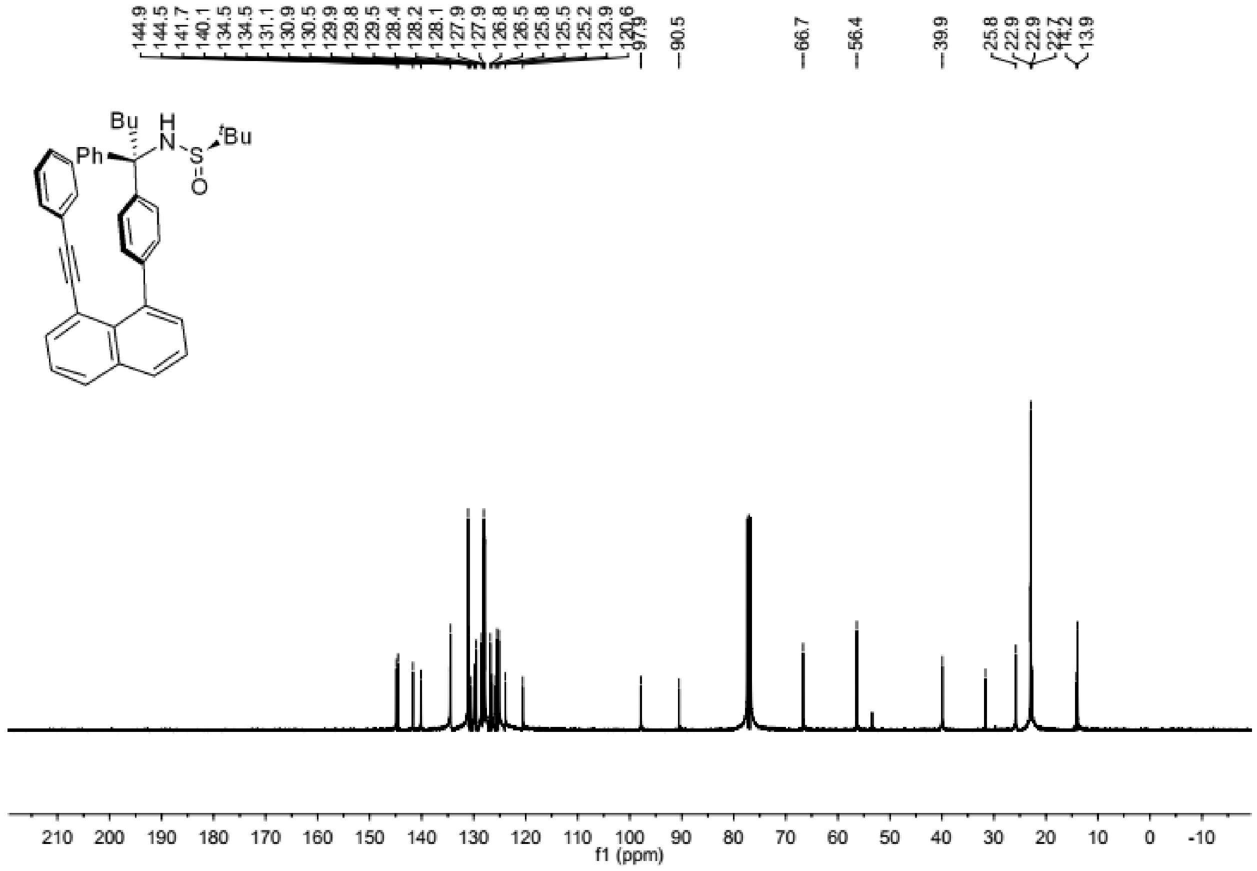

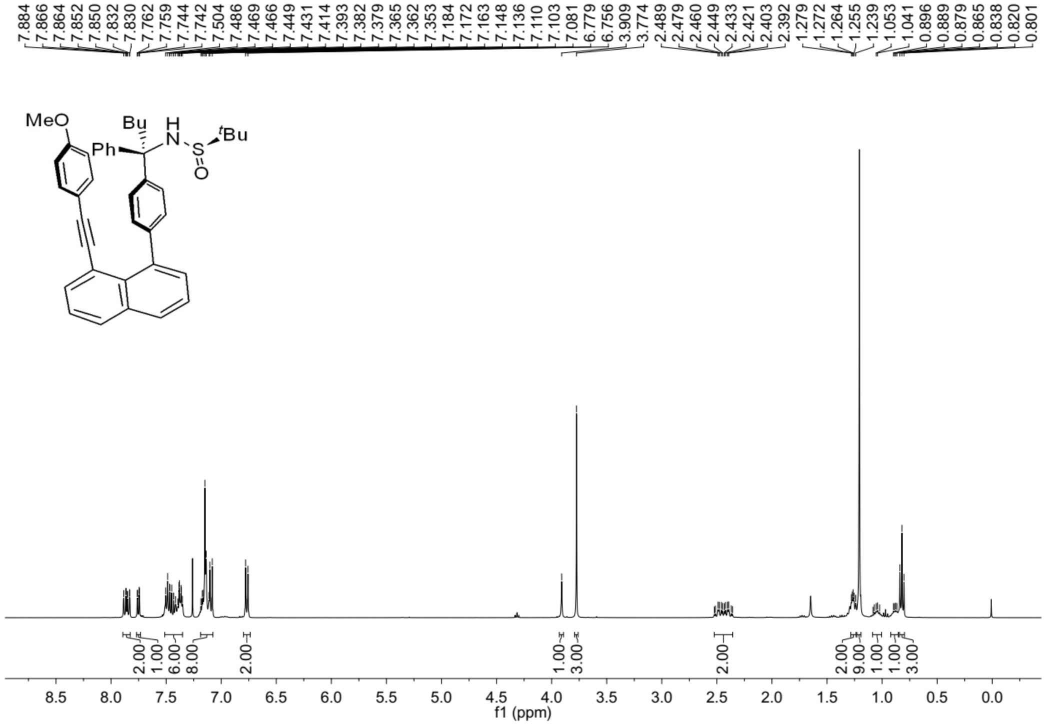

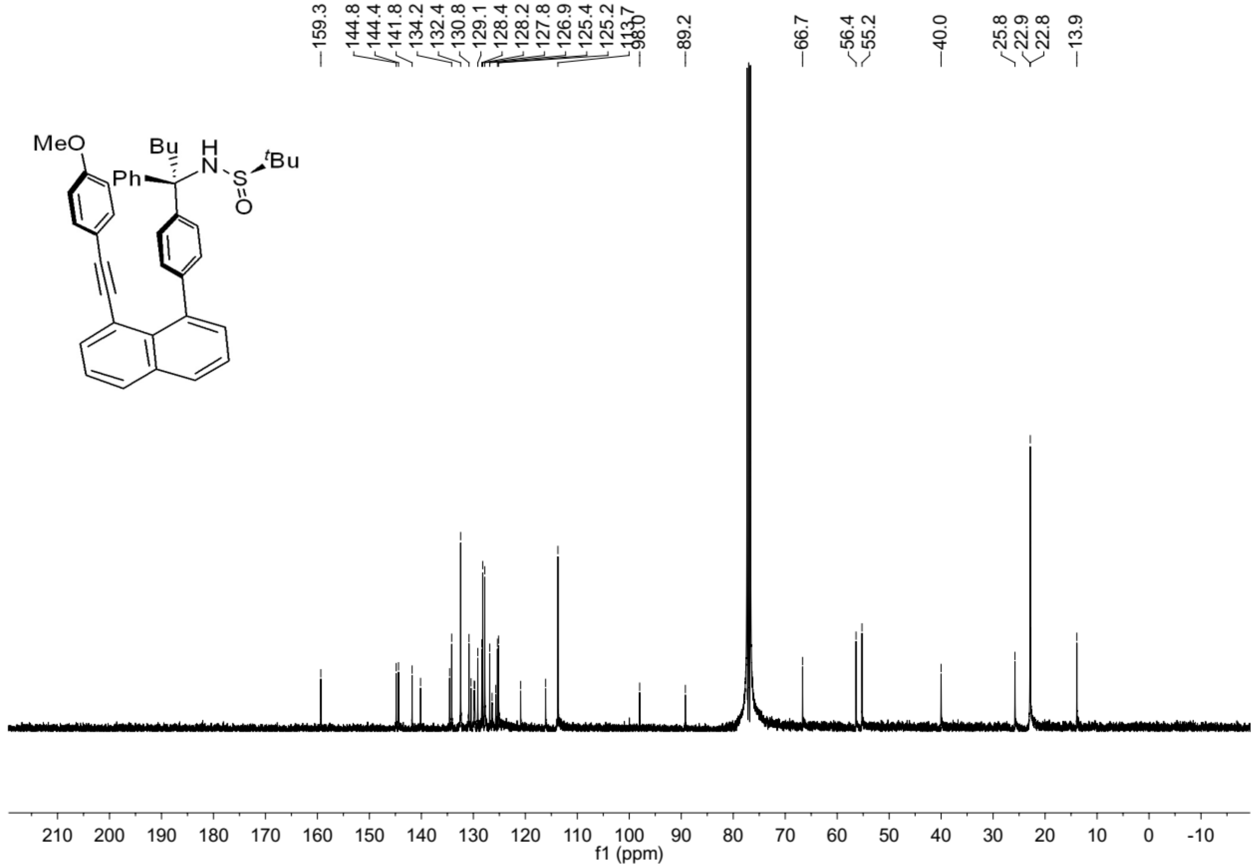

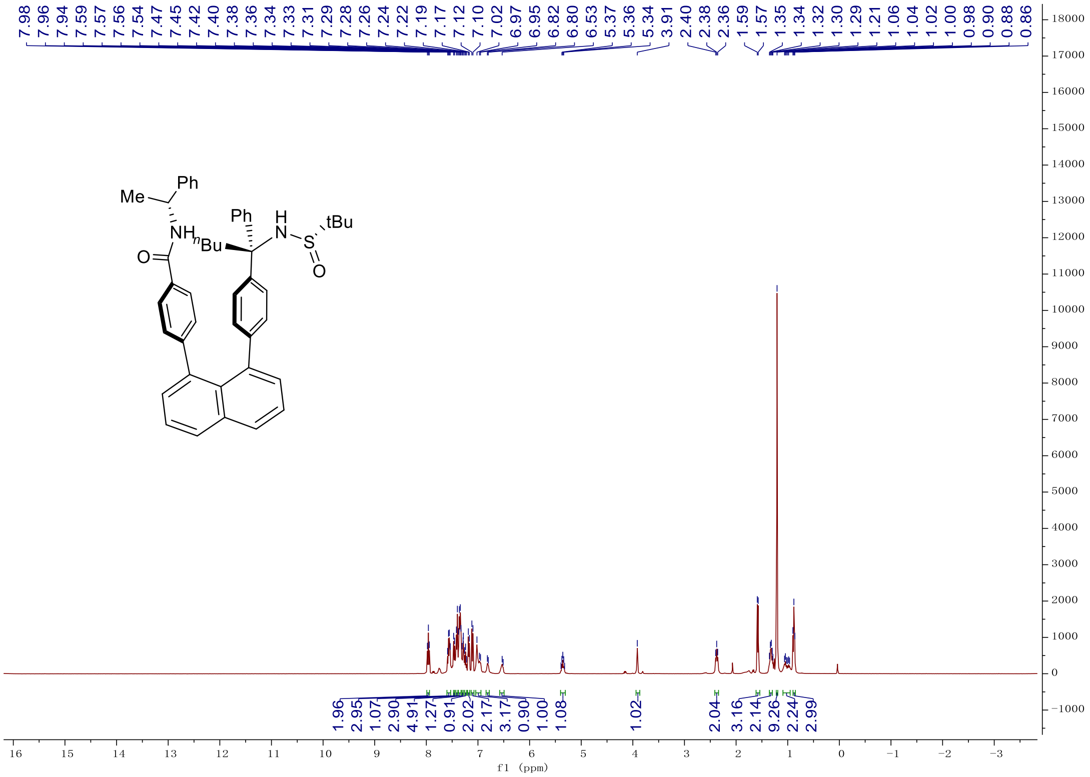

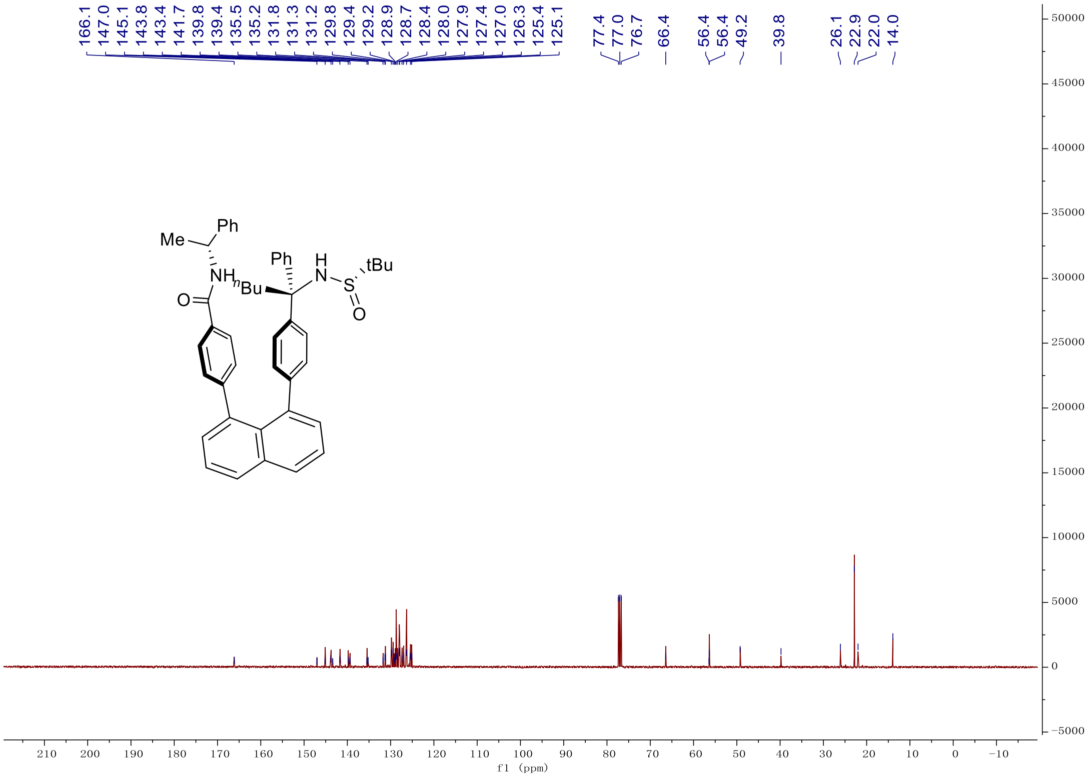

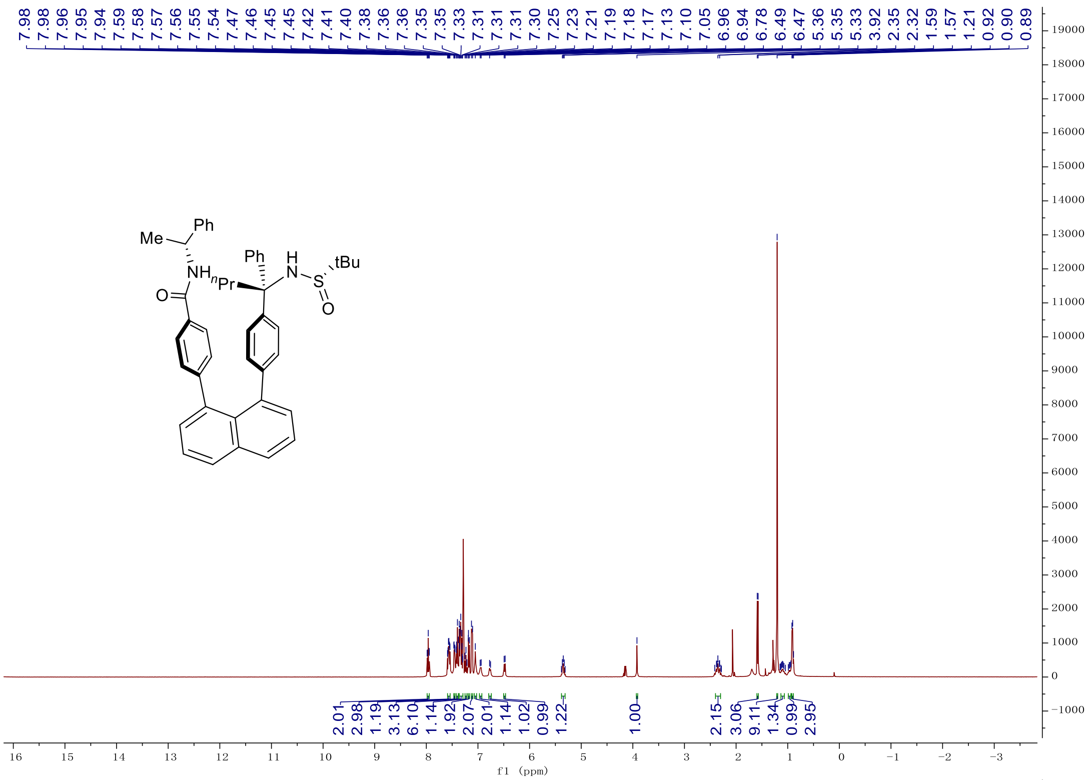

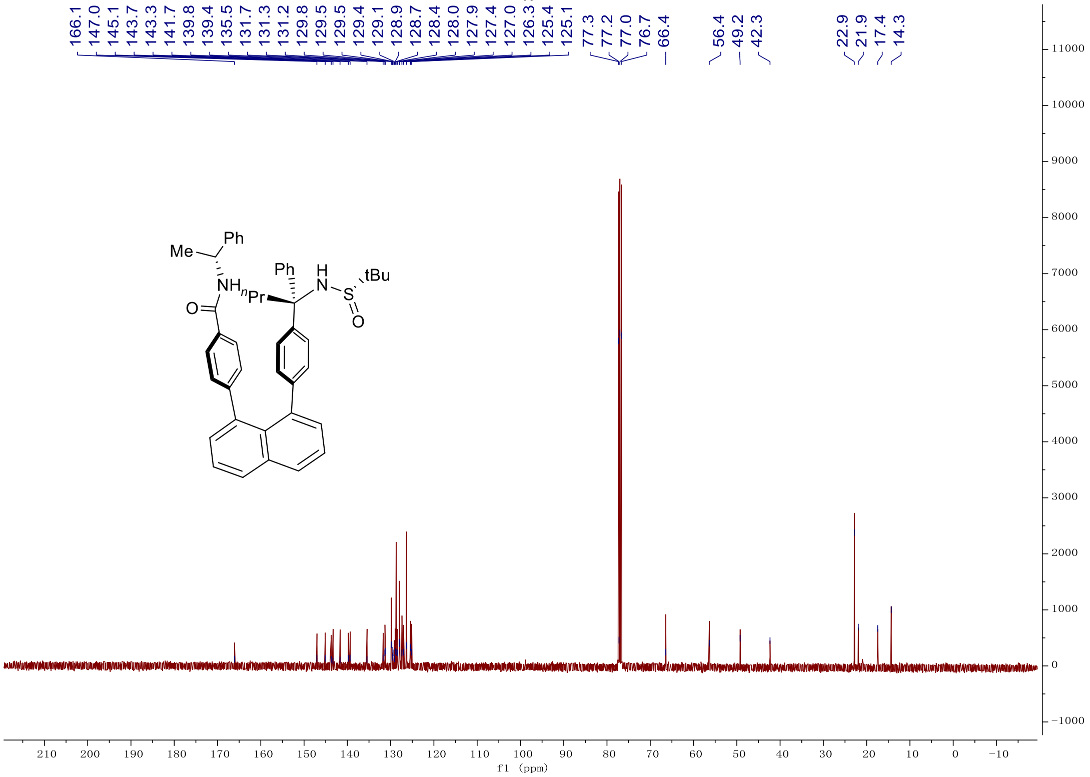

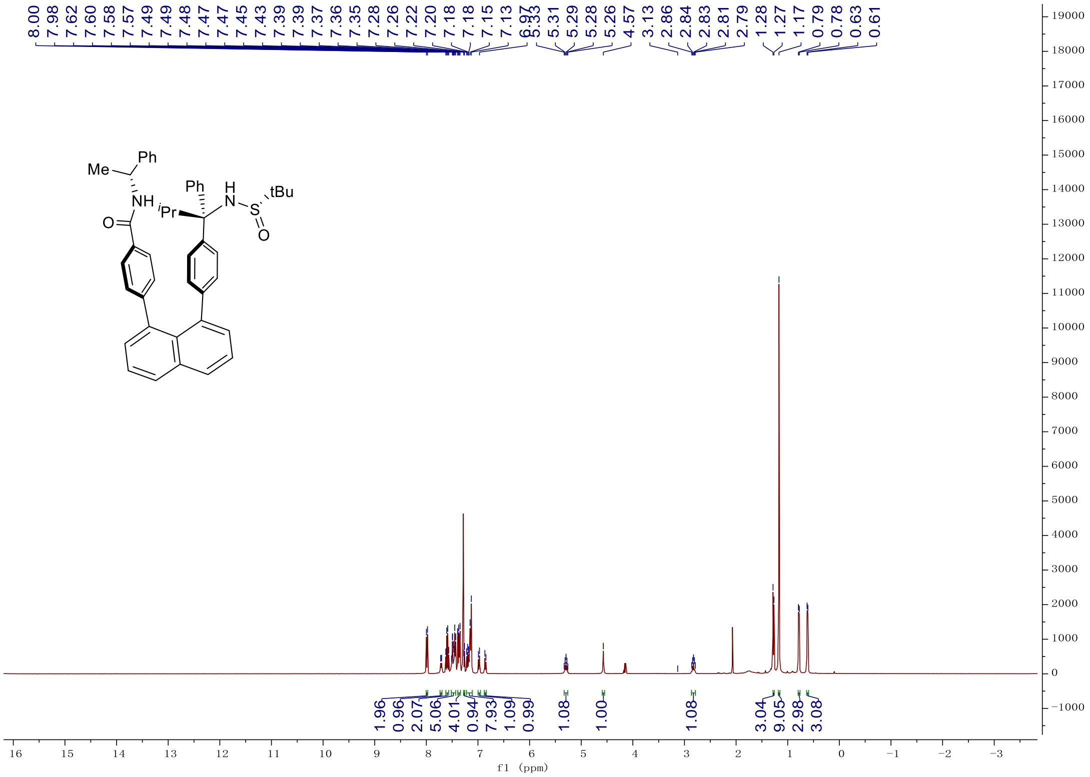
8.00 7.98 7.62 7.60 7.58 7.57 7.49 7.49 7.48 7.47 7.47 7.45 7.43 7.39 7.39 7.37 7.36 7.35 7.28 7.26 7.22 7.20 7.18 7.18 7.15 7.13 6.97 5.33 5.31 5.29 5.28 5.26 4.57 3.13 2.86 2.84 2.83 2.81 2.79 1.28 1.27 1.17 0.79 0.78 0.63 0.61
1.96 0.96 2.07 5.06 4.01 0.94 7.93 1.09 0.99 1.08 1.00 1.08 3.04 9.05 2.98 3.08
Me
Ph
NH
O
Ph
H
N
S
tBu
O
$^i$Pr
f1 (ppm)

$^1$H NMR of **10c**

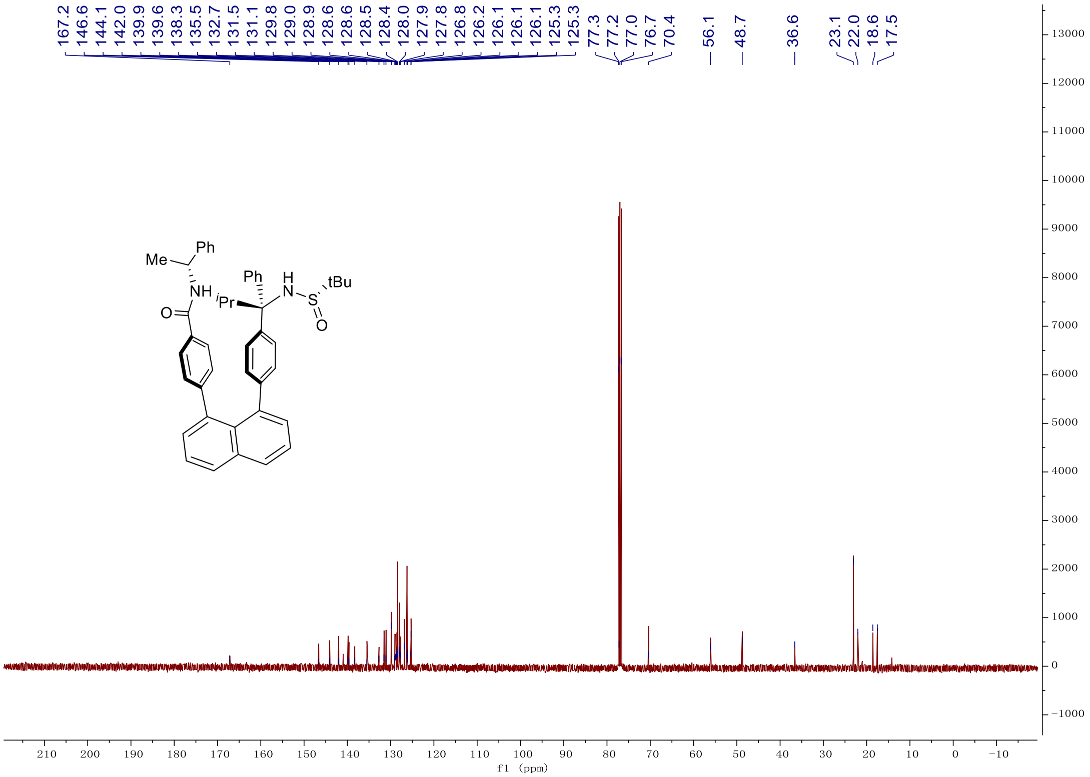
167.2 146.6 144.1 142.0 139.9 139.6 138.3 135.5 132.7 131.5 131.1 129.8 129.0 128.9 128.6 128.6 128.5 128.4 128.0 127.9 127.8 126.8 126.2 126.1 126.1 126.1 125.3 125.3 77.3 77.2 77.0 76.7 70.4 56.1 48.7 36.6 23.1 22.0 18.6 17.5
Me
Ph
NH
O
Ph
H
N
S
tBu
O
$^i$Pr
f1 (ppm)

## 3. Theoretical Calculations

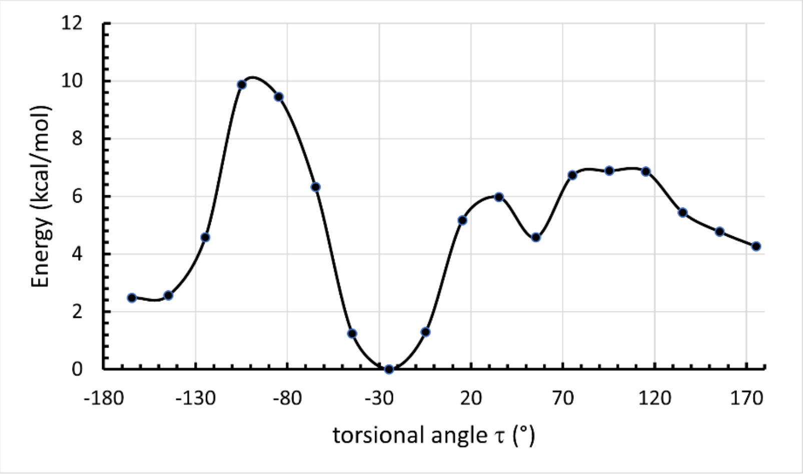


Figure S1. Torsional potential curve for Structure I-B keeping the torsional angle (Figure 11) always constant and optimizing the remaining geometry parameters. The torsional curve is periodic in τ; τ changes sign at 180°.

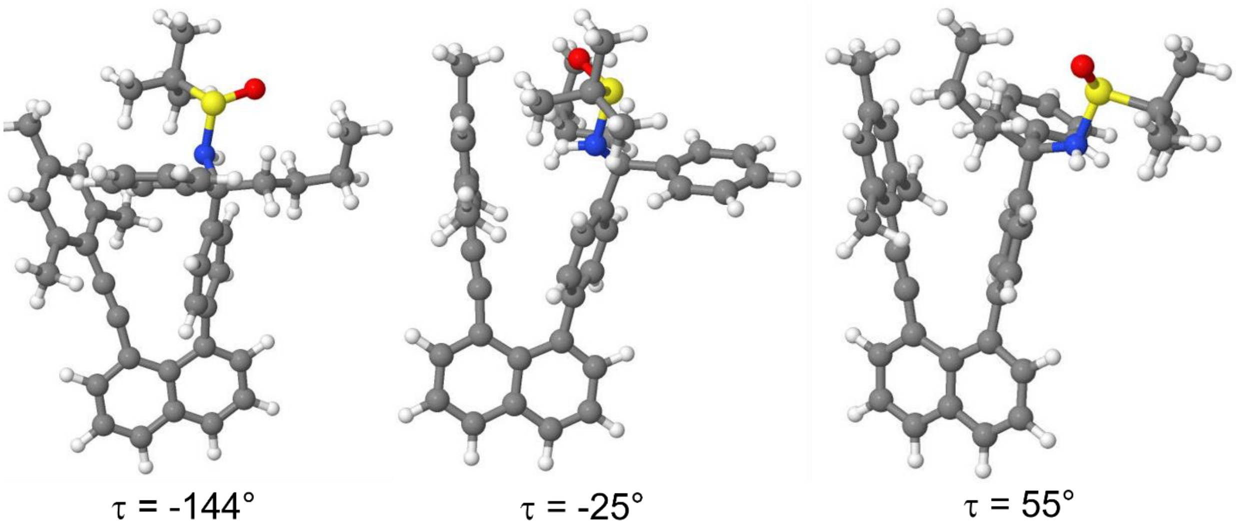


Figure S2. Structures for the three minima shown in Figure S1.

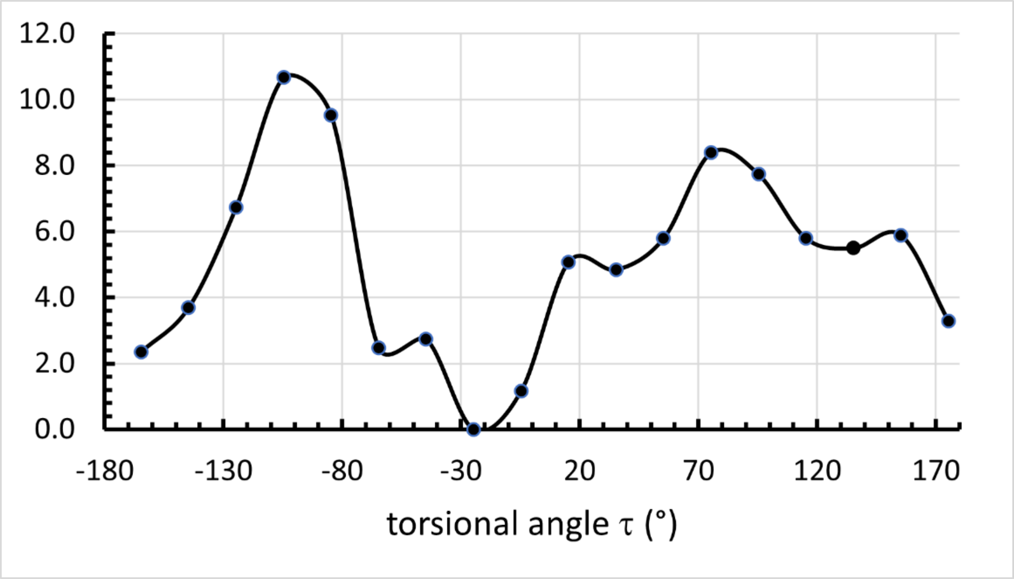


Figure S3. Torsional potential curve for Structure II-B keeping the torsional angle (Figure 11) always constant and optimizing the remaining geometry parameters. The torsional curve is periodic in τ; τ changes sign at 180°.

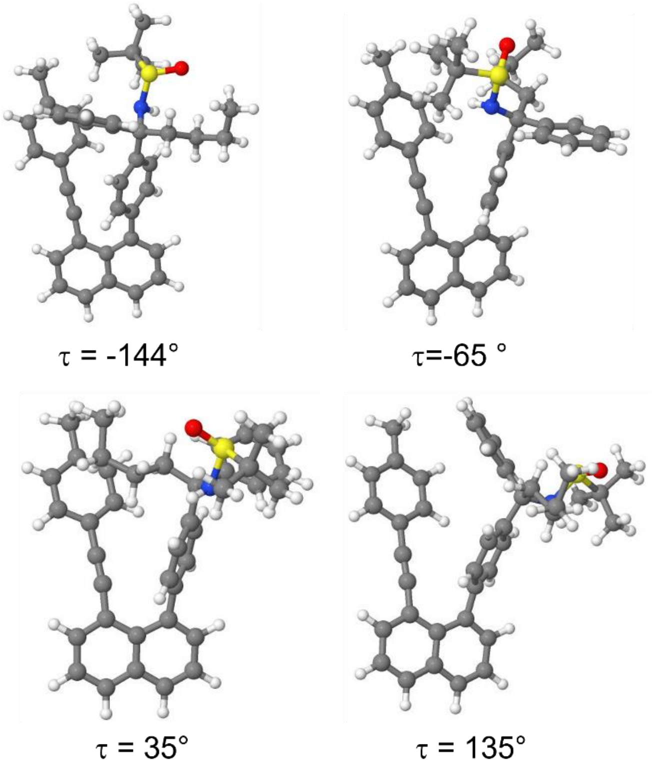


Figure S4. Structures for the five minima shown in Figure S3.

**Cartesian Coordinates** (Å)

**Structure I-B(a):** total energy -2155.816150 Hartree

| | | | |
|---|---|---|---|
| N | -2.500140 | 0.806044 | -0.568706 |
| O | -4.929575 | 1.912492 | -0.642041 |
| S | -4.196765 | 0.615734 | -0.328094 |
| C | -1.730165 | 1.330385 | 0.600392 |
| C | -2.141243 | 2.791658 | 0.954764 |
| C | -2.071252 | 3.795068 | -0.202506 |
| C | -2.312310 | 5.245488 | 0.248549 |
| C | -3.702155 | 5.493897 | 0.848251 |
| C | -1.980646 | 0.355636 | 1.767176 |
| C | -2.430758 | 0.773229 | 3.024090 |
| C | -2.645348 | -0.151030 | 4.052994 |
| C | -2.416357 | -1.507975 | 3.836529 |
| C | -1.957909 | -1.934583 | 2.585776 |
| C | -1.735194 | -1.013323 | 1.565367 |
| C | -0.230603 | 1.319164 | 0.239216 |
| C | 0.719648 | 1.298221 | 1.271991 |
| C | 2.081495 | 1.365681 | 1.001274 |
| C | 2.549447 | 1.475960 | -0.316998 |
| C | 1.606479 | 1.523441 | -1.347318 |
| C | 0.239947 | 1.429176 | -1.075091 |
| C | 4.004927 | 1.644585 | -0.583629 |
| C | 4.415097 | 2.856579 | -1.118206 |
| C | 2.389322 | -1.945288 | -0.112008 |
| C | 1.209181 | -2.721573 | -0.300772 |

| | | | |
|---|---|---|---|
| C | 0.720627 | -3.519978 | 0.763255 |
| C | 0.552757 | -2.728245 | -1.555851 |
| C | 1.398687 | -3.487580 | 2.109379 |
| C | 1.085963 | -1.907447 | -2.702382 |
| C | -4.510670 | -0.510943 | -1.809704 |
| C | -4.048027 | 0.180821 | -3.093542 |
| C | -3.779443 | -1.829627 | -1.554837 |
| C | 4.990028 | 0.669311 | -0.210128 |
| C | 5.779791 | 3.192505 | -1.248464 |
| C | 3.399929 | -1.285178 | 0.047878 |
| C | -0.407654 | -4.311146 | 0.547274 |
| C | -0.585954 | -3.520599 | -1.714325 |
| C | -6.033403 | -0.707080 | -1.815540 |
| C | 4.687110 | -0.684141 | 0.194001 |
| C | 6.376272 | 1.050930 | -0.268201 |
| C | 6.739973 | 2.318825 | -0.797385 |
| C | -1.086173 | -4.316154 | -0.677494 |
| C | 5.712165 | -1.513214 | 0.648603 |
| C | 7.382298 | 0.159815 | 0.192490 |
| C | -2.338733 | -5.138508 | -0.858306 |
| C | 7.053624 | -1.087353 | 0.671996 |
| H | -2.367946 | 1.397702 | -1.388240 |
| H | -3.166822 | 2.773039 | 1.336604 |
| H | -1.496702 | 3.139244 | 1.771955 |
| H | -2.826177 | 3.530315 | -0.953358 |
| H | -1.090915 | 3.735880 | -0.690694 |
| H | -1.541317 | 5.530249 | 0.978725 |
| H | -2.172890 | 5.905308 | -0.618123 |
| H | -3.845256 | 4.941183 | 1.783529 |

| | | | |
|---|---|---|---|
| H | -4.490231 | 5.177785 | 0.153654 |
| H | -3.851845 | 6.557350 | 1.067481 |
| H | -2.622639 | 1.820869 | 3.222842 |
| H | -2.995381 | 0.198620 | 5.020540 |
| H | -2.587544 | -2.228313 | 4.631888 |
| H | -1.768673 | -2.986709 | 2.402894 |
| H | -1.367896 | -1.355907 | 0.603205 |
| H | 0.387305 | 1.220354 | 2.302571 |
| H | 2.795372 | 1.334338 | 1.819593 |
| H | 1.943380 | 1.612283 | -2.376438 |
| H | -0.444497 | 1.441821 | -1.916007 |
| H | 3.658007 | 3.584029 | -1.397440 |
| H | 1.316816 | -2.489176 | 2.556819 |
| H | 2.468763 | -3.711138 | 2.026244 |
| H | 0.946335 | -4.206200 | 2.798950 |
| H | 2.117921 | -2.190110 | -2.944070 |
| H | 1.103028 | -0.843309 | -2.447230 |
| H | 0.474782 | -2.038015 | -3.600328 |
| H | -4.444795 | 1.200376 | -3.146724 |
| H | -4.419060 | -0.375961 | -3.961562 |
| H | -2.956238 | 0.213954 | -3.166023 |
| H | -6.553080 | 0.245785 | -1.951725 |
| H | -2.696730 | -1.692745 | -1.547864 |
| H | -4.076312 | -2.274333 | -0.597953 |
| H | -4.033482 | -2.542397 | -2.348396 |
| H | 6.059968 | 4.155462 | -1.665573 |
| H | -0.774183 | -4.936758 | 1.358759 |
| H | -1.094003 | -3.522151 | -2.675979 |

| | | | |
|---|---|---|---|
| H | -6.305556 | -1.373036 | -2.641967 |
| H | -6.383387 | -1.166677 | -0.883440 |
| H | 7.794025 | 2.580334 | -0.842453 |
| H | 5.463718 | -2.525267 | 0.952869 |
| H | 8.419522 | 0.482484 | 0.154746 |
| H | -3.200465 | -4.640083 | -0.395964 |
| H | -2.243575 | -6.122603 | -0.385781 |
| H | -2.574544 | -5.285795 | -1.916952 |
| H | 7.824399 | -1.762533 | 1.031827 |

**Structure I-B(b):** total energy -2155.819856 Hartree

| | | | |
|---|---|---|---|
| N | 2.245718 | 0.392184 | -0.497128 |
| O | 4.410891 | -1.072490 | 0.022329 |
| S | 3.953906 | 0.234809 | -0.612608 |
| C | 1.734891 | 1.179783 | 0.669817 |
| C | 2.185893 | 0.547751 | 2.014210 |
| C | 1.746269 | -0.903587 | 2.202632 |
| C | 2.068323 | -1.465209 | 3.594569 |
| C | 3.569235 | -1.579370 | 3.887464 |
| C | 2.247108 | 2.631229 | 0.512951 |
| C | 2.896512 | 3.322263 | 1.542423 |
| C | 3.335285 | 4.638247 | 1.358341 |
| C | 3.133806 | 5.286143 | 0.140882 |
| C | 2.481799 | 4.607616 | -0.893868 |
| C | 2.042152 | 3.299636 | -0.705265 |
| C | 0.195133 | 1.206963 | 0.545427 |
| C | -0.446601 | 1.029163 | -0.690005 |
| C | -1.833478 | 1.100931 | -0.801625 |

| | | | |
|---|---|---|---|
| C | -2.636782 | 1.340022 | 0.320708 |
| C | -1.997296 | 1.534601 | 1.552550 |
| C | -0.609498 | 1.470132 | 1.662872 |
| C | -4.113687 | 1.518954 | 0.206222 |
| C | -4.596505 | 2.779684 | 0.529963 |
| C | -2.356213 | -2.056571 | -0.170129 |
| C | -1.086008 | -2.697315 | -0.082711 |
| C | -0.272760 | -2.783160 | -1.240886 |
| C | -0.638957 | -3.237469 | 1.144660 |
| C | -0.762073 | -2.242932 | -2.561554 |
| C | -1.495128 | -3.118370 | 2.380706 |
| C | 4.073966 | -0.047716 | -2.473774 |
| C | 3.280057 | -1.294726 | -2.854871 |
| C | 3.560653 | 1.207148 | -3.182073 |
| C | -5.032799 | 0.508310 | -0.244893 |
| C | -5.955768 | 3.127879 | 0.390874 |
| C | -3.395987 | -1.437013 | -0.295199 |
| C | 0.984906 | -3.375357 | -1.131788 |
| C | 0.621805 | -3.836370 | 1.198056 |
| C | 5.574587 | -0.256413 | -2.725034 |
| C | -4.689667 | -0.870736 | -0.509824 |
| C | -6.414434 | 0.884218 | -0.416086 |
| C | -6.843887 | 2.199568 | -0.093722 |
| C | 1.462844 | -3.886528 | 0.082598 |
| C | -5.664680 | -1.752524 | -0.975194 |
| C | -7.365306 | -0.057450 | -0.892729 |
| C | 2.876753 | -4.403474 | 0.183131 |
| C | -6.996815 | -1.350438 | -1.180072 |

| | | | |
|---|---|---|---|
| H | 1.813858 | -0.534229 | -0.538098 |
| H | 3.278263 | 0.610004 | 2.065024 |
| H | 1.813491 | 1.149305 | 2.850149 |
| H | 2.233713 | -1.530215 | 1.449320 |
| H | 0.668577 | -0.983864 | 2.026811 |
| H | 1.587461 | -0.841690 | 4.362184 |
| H | 1.611399 | -2.459803 | 3.677964 |
| H | 4.054191 | -0.596837 | 3.914530 |
| H | 4.070887 | -2.173119 | 3.113426 |
| H | 3.748897 | -2.062211 | 4.855061 |
| H | 3.077241 | 2.848301 | 2.499748 |
| H | 3.839286 | 5.150798 | 2.173379 |
| H | 3.477198 | 6.307006 | -0.002340 |
| H | 2.313254 | 5.098521 | -1.848740 |
| H | 1.533352 | 2.786287 | -1.512248 |
| H | 0.144792 | 0.837395 | -1.577963 |
| H | -2.300092 | 0.961819 | -1.772488 |
| H | -2.595870 | 1.733686 | 2.437251 |
| H | -0.161080 | 1.629416 | 2.637387 |
| H | -3.890045 | 3.530735 | 0.871483 |
| H | -1.002922 | -1.177389 | -2.485245 |
| H | -1.680867 | -2.750042 | -2.880498 |
| H | -0.009574 | -2.372152 | -3.345015 |
| H | -2.486850 | -3.560172 | 2.227657 |
| H | -1.656185 | -2.063740 | 2.638461 |
| H | -1.025670 | -3.609502 | 3.238000 |
| H | 3.587254 | -2.147289 | -2.242139 |
| H | 3.466377 | -1.539774 | -3.906951 |
| H | 2.204310 | -1.137437 | -2.733508 |

| | | | |
|---|---|---|---|
| H | 5.948286 | -1.124232 | -2.173746 |
| H | 2.482876 | 1.322470 | -3.048364 |
| H | 4.054161 | 2.113510 | -2.813062 |
| H | 3.770115 | 1.121002 | -4.254868 |
| H | -6.284792 | 4.130791 | 0.646995 |
| H | 1.612164 | -3.443211 | -2.016057 |
| H | 0.972775 | -4.238948 | 2.145266 |
| H | 5.738960 | -0.428525 | -3.794619 |
| H | 6.158689 | 0.623881 | -2.431473 |
| H | -7.892063 | 2.451749 | -0.231843 |
| H | -5.378372 | -2.781604 | -1.168382 |
| H | -8.395491 | 0.265405 | -1.018743 |
| H | 3.578218 | -3.557844 | 0.176440 |
| H | 3.132907 | -5.047453 | -0.666327 |
| H | 3.036921 | -4.970200 | 1.105954 |
| H | -7.725538 | -2.067983 | -1.545292 |

**Structure I-B(c):** total energy -2155.812559 Hartree

| | | | |
|---|---|---|---|
| N | -2.676563 | -1.424453 | -0.287161 |
| O | -4.938133 | -0.569327 | -1.463624 |
| S | -4.382419 | -1.238512 | -0.214812 |
| C | -1.824239 | -0.282590 | 0.177119 |
| C | -2.085136 | 1.026186 | -0.636002 |
| C | -2.075856 | 0.937732 | -2.166911 |
| C | -2.607938 | 2.214078 | -2.845922 |
| C | -4.071083 | 2.552318 | -2.531574 |
| C | -2.140682 | -0.052897 | 1.669905 |

| | | | |
|---|---|---|---|
| C | -2.166755 | 1.224998 | 2.242766 |
| C | -2.397741 | 1.394087 | 3.611930 |
| C | -2.606795 | 0.288326 | 4.435081 |
| C | -2.573041 | -0.993299 | 3.877408 |
| C | -2.337854 | -1.158443 | 2.513759 |
| C | -0.360992 | -0.759107 | 0.016359 |
| C | 0.115734 | -1.240358 | -1.214076 |
| C | 1.442951 | -1.637641 | -1.373539 |
| C | 2.349186 | -1.575819 | -0.306358 |
| C | 1.868096 | -1.130827 | 0.931061 |
| C | 0.546483 | -0.722682 | 1.084743 |
| C | 3.747006 | -2.066720 | -0.459911 |
| C | 3.898415 | -3.380944 | -0.880670 |
| C | 2.862588 | 1.767934 | 0.022433 |
| C | 1.748654 | 2.652879 | -0.067173 |
| C | 1.311485 | 3.095635 | -1.339300 |
| C | 1.090246 | 3.088944 | 1.107152 |
| C | 1.988040 | 2.593485 | -2.590230 |
| C | 1.557132 | 2.622718 | 2.463305 |
| C | -4.818612 | -3.067691 | -0.419868 |
| C | -4.311074 | -3.562745 | -1.775967 |
| C | -4.223871 | -3.852893 | 0.750431 |
| C | 4.916296 | -1.293348 | -0.132534 |
| C | 5.158127 | -4.010586 | -0.950560 |
| C | 3.771395 | 0.962005 | 0.089153 |
| C | 0.242439 | 3.987706 | -1.408217 |
| C | 0.005919 | 3.961440 | 0.983811 |
| C | -6.354077 | -3.088364 | -0.377689 |
| C | 4.919063 | 0.117711 | 0.186430 |

| | | | |
|---|---|---|---|
| C | 6.192476 | -1.962649 | -0.154121 |
| C | 6.281244 | -3.319281 | -0.567174 |
| C | -0.435581 | 4.421645 | -0.261818 |
| C | 6.109558 | 0.744185 | 0.552882 |
| C | 7.375397 | -1.271896 | 0.222694 |
| C | -1.644691 | 5.317313 | -0.377867 |
| C | 7.334004 | 0.052484 | 0.589936 |
| H | -2.395300 | -1.756145 | -1.207556 |
| H | -3.066217 | 1.394081 | -0.317801 |
| H | -1.349463 | 1.776300 | -0.329117 |
| H | -2.691916 | 0.093943 | -2.496802 |
| H | -1.054438 | 0.764927 | -2.523465 |
| H | -1.970839 | 3.062319 | -2.570782 |
| H | -2.496121 | 2.087351 | -3.931428 |
| H | -4.199526 | 2.870559 | -1.490316 |
| H | -4.717398 | 1.682135 | -2.684950 |
| H | -4.424612 | 3.372342 | -3.167887 |
| H | -1.993108 | 2.105871 | 1.637620 |
| H | -2.411568 | 2.397485 | 4.029299 |
| H | -2.788984 | 0.420531 | 5.498098 |
| H | -2.725910 | -1.866948 | 4.505586 |
| H | -2.296867 | -2.154972 | 2.091162 |
| H | -0.540084 | -1.308671 | -2.075178 |
| H | 1.782312 | -1.991273 | -2.343257 |
| H | 2.540983 | -1.090098 | 1.782425 |
| H | 0.221337 | -0.376097 | 2.057300 |
| H | 3.007044 | -3.952617 | -1.122729 |
| H | 1.911429 | 1.501166 | -2.658288 |

| | | | |
|---|---|---|---|
| H | 3.058054 | 2.833764 | -2.591958 |
| H | 1.535128 | 3.026048 | -3.487058 |
| H | 2.561915 | 3.002940 | 2.687120 |
| H | 1.617315 | 1.530533 | 2.504214 |
| H | 0.879378 | 2.960509 | 3.252628 |
| H | -4.612618 | -2.875839 | -2.573304 |
| H | -4.742730 | -4.547569 | -1.987529 |
| H | -3.221678 | -3.673582 | -1.789589 |
| H | -6.779036 | -2.492135 | -1.190327 |
| H | -3.132501 | -3.871205 | 0.701587 |
| H | -4.523097 | -3.427352 | 1.715169 |
| H | -4.590965 | -4.885394 | 0.710258 |
| H | 5.226906 | -5.044369 | -1.276477 |
| H | -0.079953 | 4.345742 | -2.383069 |
| H | -0.507806 | 4.290691 | 1.884610 |
| H | -6.701842 | -4.121548 | -0.487908 |
| H | -6.735494 | -2.703507 | 0.575542 |
| H | 7.258193 | -3.795566 | -0.578913 |
| H | 6.083076 | 1.802755 | 0.791932 |
| H | 8.317935 | -1.812959 | 0.208629 |
| H | -2.540609 | 4.726185 | -0.611448 |
| H | -1.525580 | 6.051567 | -1.182142 |
| H | -1.839899 | 5.855625 | 0.555147 |
| H | 8.239377 | 0.576310 | 0.881814 |

**Structure II-B(a):** total energy -2077.170906 Hartree

| | | | |
|---|---|---|---|
| N | -2.481805000 | 0.736744000 | -0.575172000 |
| O | -4.819618001 | 2.022762000 | -0.543917000 |
| S | -4.181086000 | 0.666313000 | -0.280282000 |
| C | -1.643728000 | 1.151708000 | 0.589606000 |
| C | -1.955726000 | 2.615856000 | 1.022475000 |
| C | -1.816492000 | 3.659108000 | -0.092969000 |
| C | -1.927002000 | 5.104489001 | 0.418897000 |
| C | -3.280994000 | 5.440442001 | 1.056415000 |
| C | -1.908948000 | 0.132488000 | 1.714818000 |
| C | -2.333961000 | 0.502969000 | 2.994866000 |
| C | -2.573703000 | -0.464866000 | 3.977202000 |
| C | -2.395602000 | -1.816916000 | 3.690561000 |
| C | -1.962214000 | -2.196776000 | 2.415794000 |
| C | -1.714707000 | -1.232191000 | 1.442082000 |
| C | -0.157994000 | 1.073826000 | 0.185203000 |
| C | 0.811810000 | 1.186055000 | 1.194528000 |
| C | 2.167056000 | 1.213812000 | 0.891924000 |
| C | 2.610021000 | 1.138072000 | -0.438671000 |
| C | 1.648056000 | 1.023359000 | -1.446026000 |
| C | 0.284912000 | 0.977590000 | -1.137744000 |
| C | 4.060780000 | 1.300033000 | -0.743115000 |
| C | 4.436205001 | 2.441304000 | -1.435976000 |
| C | 2.540883000 | -2.187093000 | 0.052959000 |
| C | 1.331493000 | -2.887073000 | -0.218555000 |
| C | 0.867778000 | -3.911229000 | 0.629656000 |

| | | | |
|---|---|---|---|
| C | 0.581765000 | -2.577902000 | -1.371812000 |
| C | -4.631128001 | -0.400032000 | -1.769763000 |
| C | -4.138899000 | 0.268512000 | -3.054571000 |
| C | -4.014293000 | -1.781920000 | -1.550374000 |
| C | 5.076583001 | 0.419042000 | -0.239115000 |
| C | 5.790476001 | 2.802933000 | -1.603537000 |
| C | 3.555051000 | -1.534412000 | 0.213101000 |
| C | -0.311158000 | -4.589974001 | 0.335180000 |
| C | -0.593189000 | -3.267120000 | -1.654107000 |
| C | -6.164959001 | -0.460881000 | -1.740882000 |
| C | 4.817062001 | -0.883335000 | 0.331455000 |
| C | 6.448612001 | 0.839530000 | -0.333409000 |
| C | 6.774981001 | 2.036071000 | -1.027525000 |
| C | -1.069113000 | -4.276569001 | -0.803130000 |
| C | 5.860516001 | -1.607477000 | 0.906172000 |
| C | 7.476637001 | 0.055813000 | 0.256799000 |
| C | -2.373360000 | -4.982124001 | -1.081735000 |
| C | 7.184181001 | -1.128553000 | 0.894205000 |
| H | -2.331923000 | 1.359365000 | -1.369495000 |
| H | -2.978264000 | 2.650118000 | 1.411281000 |
| H | -1.288267000 | 2.884175000 | 1.850272000 |
| H | -2.600451000 | 3.490872000 | -0.842228000 |
| H | -0.851376000 | 3.533987000 | -0.599772000 |
| H | -1.122373000 | 5.294825001 | 1.143438000 |
| H | -1.748590000 | 5.785571001 | -0.423869000 |
| H | -3.453461000 | 4.862149001 | 1.971139000 |
| H | -4.103980000 | 5.219178001 | 0.365577000 |
| H | -3.338971000 | 6.502346001 | 1.321708000 |
| H | -2.488775000 | 1.545724000 | 3.246094000 |

| | | | |
|---|---|---|---|
| H | -2.904463000 | -0.153274000 | 4.964394001 |
| H | -2.586992000 | -2.569193000 | 4.451069001 |
| H | -1.807823000 | -3.244485000 | 2.178170000 |
| H | -1.368877000 | -1.536655000 | 0.460054000 |
| H | 0.498151000 | 1.249115000 | 2.232748000 |
| H | 2.896812000 | 1.299868000 | 1.692195000 |
| H | 1.965639000 | 0.955118000 | -2.482990000 |
| H | -0.420551000 | 0.854051000 | -1.951719000 |
| H | 3.658292000 | 3.099966000 | -1.811962000 |
| H | -4.446515001 | 1.319397000 | -3.083931000 |
| H | -4.576995001 | -0.240189000 | -3.920815000 |
| H | -3.050315000 | 0.207951000 | -3.151788000 |
| H | -6.601068001 | 0.535777000 | -1.854286000 |
| H | -2.924220000 | -1.731360000 | -1.539259000 |
| H | -4.348292001 | -2.224992000 | -0.605073000 |
| H | -4.325008001 | -2.449244000 | -2.362833000 |
| H | 6.042256001 | 3.708963000 | -2.147142000 |
| H | -0.653141000 | -5.376478001 | 1.003840000 |
| H | -1.152262000 | -3.018133000 | -2.552260000 |
| H | -6.514245001 | -1.089110000 | -2.567883000 |
| H | -6.532885001 | -0.899912000 | -0.805810000 |
| H | 7.819577001 | 2.328159000 | -1.098184000 |
| H | 5.644143001 | -2.581945000 | 1.333009000 |
| H | 8.502296001 | 0.409284000 | 0.190445000 |
| H | -3.193352000 | -4.512178001 | -0.523304000 |
| H | -2.333841000 | -6.033553001 | -0.777693000 |
| H | -2.635796000 | -4.938792001 | -2.143524000 |
| H | 7.972468001 | -1.719194000 | 1.351498000 |

| | | | |
|---|---|---|---|
| H | 1.437099000 | -4.166744000 | 1.518252000 |
| H | 0.927163000 | -1.790267000 | -2.032597000 |

**Structure II-B(b):** total energy -2077.174563 Hartree

| | | | |
|---|---|---|---|
| N | 2.304955 | 0.365003 | -0.435447 |
| O | 4.342679 | -1.267306 | 0.099574 |
| S | 4.007320 | 0.162877 | -0.305232 |
| C | 1.605996 | 0.920278 | 0.768081 |
| C | 1.843221 | 0.025804 | 2.015101 |
| C | 1.405081 | -1.429681 | 1.850703 |
| C | 1.589193 | -2.270523 | 3.122064 |
| C | 3.052780 | -2.432608 | 3.551941 |
| C | 2.124304 | 2.361529 | 0.986849 |
| C | 2.602055 | 2.818498 | 2.220762 |
| C | 3.055079 | 4.133685 | 2.373526 |
| C | 3.040203 | 5.015741 | 1.294604 |
| C | 2.559795 | 4.573020 | 0.057954 |
| C | 2.105100 | 3.264781 | -0.088676 |
| C | 0.107162 | 1.002573 | 0.400617 |
| C | -0.319302 | 1.090771 | -0.932253 |
| C | -1.673569 | 1.188906 | -1.254429 |
| C | -2.652571 | 1.179698 | -0.255490 |
| C | -2.228601 | 1.112105 | 1.079671 |
| C | -0.876866 | 1.034733 | 1.400475 |
| C | -4.099599 | 1.360309 | -0.569937 |
| C | -4.477535 | 2.585817 | -1.097878 |
| C | -2.520591 | -2.197407 | -0.124963 |
| C | -1.252800 | -2.805446 | -0.351262 |

| | | | |
|---|---|---|---|
| C | -0.435661 | -2.347690 | -1.406469 |
| C | -0.769282 | -3.842041 | 0.468464 |
| C | 4.430818 | 0.271061 | -2.139873 |
| C | 3.677736 | -0.810387 | -2.912997 |
| C | 4.078694 | 1.678569 | -2.624648 |
| C | -5.110156 | 0.392442 | -0.245257 |
| C | -5.832564 | 2.942776 | -1.267452 |
| C | -3.557886 | -1.579072 | 0.025429 |
| C | 0.830793 | -2.884741 | -1.599022 |
| C | 0.508204 | -4.363479 | 0.268931 |
| C | 5.946082 | 0.026171 | -2.191891 |
| C | -4.843025 | -0.973954 | 0.145353 |
| C | -6.488507 | 0.794641 | -0.342102 |
| C | -6.818303 | 2.075646 | -0.861592 |
| C | 1.340332 | -3.881353 | -0.749485 |
| C | -5.894035 | -1.795727 | 0.550954 |
| C | -7.521959 | -0.088845 | 0.070956 |
| C | 2.763706 | -4.354510 | -0.905907 |
| C | -7.228635 | -1.349258 | 0.538721 |
| H | 1.879954 | -0.519615 | -0.723722 |
| H | 2.913196 | 0.060649 | 2.246689 |
| H | 1.336466 | 0.458491 | 2.884463 |
| H | 1.981881 | -1.892962 | 1.045371 |
| H | 0.354481 | -1.466061 | 1.546589 |
| H | 1.007412 | -1.830244 | 3.945113 |
| H | 1.158137 | -3.262633 | 2.935545 |
| H | 3.491359 | -1.479877 | 3.870921 |
| H | 3.659800 | -2.809666 | 2.719868 |

| | | | |
|---|---|---|---|
| H | 3.145659 | -3.134204 | 4.389179 |
| H | 2.635739 | 2.160178 | 3.080436 |
| H | 3.423409 | 4.461027 | 3.342146 |
| H | 3.395079 | 6.035853 | 1.413024 |
| H | 2.537247 | 5.248088 | -0.793483 |
| H | 1.729751 | 2.936056 | -1.050345 |
| H | 0.411964 | 1.072353 | -1.732481 |
| H | -1.974313 | 1.251478 | -2.296836 |
| H | -2.968754 | 1.121127 | 1.875070 |
| H | -0.595639 | 0.994120 | 2.447232 |
| H | -3.702315 | 3.307204 | -1.340704 |
| H | 3.838463 | -1.794139 | -2.461022 |
| H | 4.045361 | -0.842969 | -3.945070 |
| H | 2.604621 | -0.599167 | -2.948967 |
| H | 6.198298 | -0.963231 | -1.799675 |
| H | 2.997777 | 1.833774 | -2.633019 |
| H | 4.533999 | 2.451195 | -1.994773 |
| H | 4.456186 | 1.808229 | -3.645845 |
| H | -6.085923 | 3.915879 | -1.678141 |
| H | 1.443163 | -2.522157 | -2.419449 |
| H | 0.873354 | -5.143327 | 0.932417 |
| H | 6.282729 | 0.082992 | -3.232974 |
| H | 6.496265 | 0.781520 | -1.618215 |
| H | -7.866848 | 2.352067 | -0.936241 |
| H | -5.670799 | -2.816664 | 0.844899 |
| H | -8.552339 | 0.250917 | 0.005874 |
| H | 3.453982 | -3.558957 | -0.593531 |
| H | 2.994353 | -4.593989 | -1.950831 |
| H | 2.965243 | -5.241703 | -0.297582 |

| | | | |
|---|---|---|---|
| H | -8.021255 | -2.017849 | 0.861370 |
| H | -0.800284 | -1.557194 | -2.053069 |
| H | -1.389405 | -4.216184 | 1.277595 |

**Structure II-B(c):** total energy -2077.166866 Hartree

| | | | |
|---|---|---|---|
| N | -2.624226 | -1.119094 | -0.740809 |
| O | -4.629175 | 0.301180 | -1.812737 |
| S | -4.322772 | -0.863832 | -0.882030 |
| C | -1.913185 | -0.287585 | 0.280228 |
| C | -1.937113 | 1.224659 | -0.095139 |
| C | -1.493492 | 1.547852 | -1.527945 |
| C | -1.304272 | 3.051952 | -1.779984 |
| C | -2.533699 | 3.908743 | -1.456631 |
| C | -2.597742 | -0.568456 | 1.630712 |
| C | -3.011477 | 0.442535 | 2.503324 |
| C | -3.606248 | 0.127588 | 3.731228 |
| C | -3.796090 | -1.202110 | 4.102314 |
| C | -3.377581 | -2.221830 | 3.239939 |
| C | -2.780999 | -1.905535 | 2.022637 |
| C | -0.445067 | -0.765848 | 0.351381 |
| C | 0.245359 | -1.253893 | -0.767045 |
| C | 1.593256 | -1.599188 | -0.688999 |
| C | 2.299444 | -1.488453 | 0.515673 |
| C | 1.610629 | -1.022251 | 1.641292 |
| C | 0.268413 | -0.655937 | 1.554793 |
| C | 3.702273 | -1.987709 | 0.592039 |
| C | 3.923441 | -3.141611 | 1.328204 |

| | | | |
|---|---|---|---|
| C | 2.991986 | 1.790402 | -0.313605 |
| C | 2.158805 | 2.893108 | 0.029652 |
| C | 2.248479 | 4.113182 | -0.671126 |
| C | 1.251593 | 2.803516 | 1.105345 |
| C | -4.693938 | -2.417966 | -1.890743 |
| C | -3.904406 | -2.382928 | -3.201036 |
| C | -4.354110 | -3.637522 | -1.031139 |
| C | 4.779184 | -1.418590 | -0.166217 |
| C | 5.162077 | -3.818446 | 1.301304 |
| C | 3.712828 | 0.839631 | -0.556977 |
| C | 1.447903 | 5.194032 | -0.312024 |
| C | 0.457746 | 3.892235 | 1.451571 |
| C | -6.202665 | -2.330829 | -2.163424 |
| C | 4.727247 | -0.128745 | -0.816017 |
| C | 6.007309 | -2.161408 | -0.261755 |
| C | 6.170177 | -3.357159 | 0.488919 |
| C | 0.532053 | 5.103206 | 0.746970 |
| C | 5.784414 | 0.274936 | -1.631894 |
| C | 7.059328 | -1.696525 | -1.096008 |
| C | -0.383779 | 6.253844 | 1.084151 |
| C | 6.936210 | -0.517120 | -1.794844 |
| H | -2.208129 | -0.999927 | -1.662883 |
| H | -2.960788 | 1.589060 | 0.038900 |
| H | -1.303152 | 1.768413 | 0.613708 |
| H | -2.245549 | 1.160209 | -2.225660 |
| H | -0.545785 | 1.043670 | -1.752879 |
| H | -0.451754 | 3.409050 | -1.194802 |
| H | -1.034314 | 3.191286 | -2.835740 |
| H | -2.749098 | 3.911778 | -0.381903 |

| | | | |
|---|---|---|---|
| H | -3.427212 | 3.535004 | -1.972606 |
| H | -2.376841 | 4.950132 | -1.761599 |
| H | -2.874715 | 1.486080 | 2.244241 |
| H | -3.920780 | 0.929916 | 4.393162 |
| H | -4.260864 | -1.445298 | 5.053857 |
| H | -3.513561 | -3.263292 | 3.519051 |
| H | -2.452201 | -2.697339 | 1.357671 |
| H | -0.252873 | -1.382066 | -1.721910 |
| H | 2.101511 | -1.973938 | -1.573032 |
| H | 2.132427 | -0.935394 | 2.590402 |
| H | -0.233469 | -0.287035 | 2.443230 |
| H | 3.098867 | -3.560393 | 1.898386 |
| H | -4.027841 | -1.415906 | -3.700007 |
| H | -4.278013 | -3.164974 | -3.871862 |
| H | -2.837781 | -2.571538 | -3.040734 |
| H | -6.447496 | -1.433258 | -2.738772 |
| H | -3.279648 | -3.702777 | -0.844288 |
| H | -4.873695 | -3.606904 | -0.066322 |
| H | -4.673777 | -4.545165 | -1.556822 |
| H | 5.293961 | -4.723556 | 1.887072 |
| H | 1.529051 | 6.124188 | -0.869240 |
| H | -0.241304 | 3.797516 | 2.279288 |
| H | -6.515334 | -3.208495 | -2.739902 |
| H | -6.779142 | -2.317185 | -1.230795 |
| H | 7.111408 | -3.895186 | 0.411419 |
| H | 5.725951 | 1.245341 | -2.114994 |
| H | 7.967169 | -2.289887 | -1.167508 |
| H | -1.328061 | 6.167195 | 0.529994 |

| | | | |
|---|---|---|---|
| H | 0.063929 | 7.216975 | 0.818192 |
| H | -0.632611 | 6.270939 | 2.150438 |
| H | 7.738870 | -0.169742 | -2.438650 |
| H | 1.174726 | 1.871919 | 1.654788 |
| H | 2.949486 | 4.203479 | -1.495596 |

**Structure II-B(d):** total energy -2077.165969 Hartree

| | | | |
|---|---|---|---|
| N | 2.703227 | -1.202941 | -0.291005 |
| O | 4.970982 | -1.919381 | 0.971327 |
| S | 4.421053 | -1.163040 | -0.229544 |
| C | 1.940879 | -0.216724 | 0.539586 |
| C | 2.244500 | -0.429603 | 2.049358 |
| C | 2.062131 | -1.868694 | 2.554860 |
| C | 2.098924 | -1.971349 | 4.087687 |
| C | 3.425215 | -1.517306 | 4.709992 |
| C | 2.351668 | 1.202534 | 0.079907 |
| C | 2.480666 | 2.266480 | 0.982268 |
| C | 2.930643 | 3.520465 | 0.558488 |
| C | 3.259115 | 3.738837 | -0.778837 |
| C | 3.099288 | 2.697254 | -1.697029 |
| C | 2.641155 | 1.450245 | -1.272519 |
| C | 0.451539 | -0.486882 | 0.209507 |
| C | -0.554630 | -0.437522 | 1.186585 |
| C | -1.861678 | -0.829466 | 0.903982 |
| C | -2.230749 | -1.261536 | -0.378581 |
| C | -1.245648 | -1.225426 | -1.378122 |
| C | 0.066951 | -0.852961 | -1.089241 |
| C | -3.542487 | -1.929852 | -0.628473 |

| | | | |
|---|---|---|---|
| C | -3.464215 | -3.204344 | -1.179700 |
| C | -3.420827 | 1.954066 | 0.043277 |
| C | -2.477425 | 3.008070 | -0.087133 |
| C | -2.755329 | 4.309387 | 0.374342 |
| C | -1.231643 | 2.757369 | -0.698899 |
| C | 4.716604 | -2.308970 | -1.706054 |
| C | 4.095202 | -3.678694 | -1.425552 |
| C | 4.142099 | -1.646916 | -2.960090 |
| C | -4.832698 | -1.423615 | -0.235948 |
| C | -4.588335 | -4.042849 | -1.319796 |
| C | -4.145450 | 0.980083 | 0.118712 |
| C | -1.811010 | 5.322671 | 0.228849 |
| C | -0.304223 | 3.780784 | -0.843404 |
| C | 6.246583 | -2.416532 | -1.787093 |
| C | -5.099057 | -0.070955 | 0.199610 |
| C | -5.962535 | -2.315311 | -0.313840 |
| C | -5.810830 | -3.616546 | -0.862595 |
| C | -0.571288 | 5.078726 | -0.379627 |
| C | -6.374786 | 0.283031 | 0.641865 |
| C | -7.242875 | -1.899994 | 0.141168 |
| C | 0.465541 | 6.168235 | -0.507796 |
| C | -7.441726 | -0.631801 | 0.635540 |
| H | 2.355092 | -2.146297 | -0.121696 |
| H | 3.279395 | -0.118473 | 2.221376 |
| H | 1.624114 | 0.243888 | 2.649818 |
| H | 2.858007 | -2.493888 | 2.135715 |
| H | 1.108205 | -2.274869 | 2.196827 |
| H | 1.273590 | -1.381458 | 4.512133 |

| | | | |
|---|---|---|---|
| H | 1.907521 | -3.014897 | 4.371436 |
| H | 3.602827 | -0.447922 | 4.549326 |
| H | 4.269522 | -2.062133 | 4.269865 |
| H | 3.435085 | -1.694287 | 5.791665 |
| H | 2.248997 | 2.129834 | 2.032004 |
| H | 3.031075 | 4.323593 | 1.283479 |
| H | 3.619763 | 4.710102 | -1.105093 |
| H | 3.335803 | 2.851762 | -2.746387 |
| H | 2.543699 | 0.648063 | -1.993586 |
| H | -0.321952 | -0.139349 | 2.202364 |
| H | -2.596965 | -0.835733 | 1.702533 |
| H | -1.501876 | -1.526569 | -2.390228 |
| H | 0.808192 | -0.886123 | -1.879394 |
| H | -2.488603 | -3.588170 | -1.462172 |
| H | 4.389546 | -4.038727 | -0.434281 |
| H | 4.451247 | -4.398032 | -2.171729 |
| H | 3.002302 | -3.656013 | -1.490698 |
| H | 6.659109 | -2.853193 | -0.873079 |
| H | 3.051639 | -1.590122 | -2.916261 |
| H | 4.540114 | -0.635190 | -3.099517 |
| H | 4.422424 | -2.241172 | -3.837880 |
| H | -4.470420 | -5.033270 | -1.749706 |
| H | -2.041183 | 6.321630 | 0.591884 |
| H | 0.647298 | 3.566986 | -1.316754 |
| H | 6.516596 | -3.060369 | -2.631547 |
| H | 6.710539 | -1.436185 | -1.947815 |
| H | -6.682647 | -4.263267 | -0.916962 |
| H | -6.542579 | 1.304589 | 0.968444 |
| H | -8.066619 | -2.606681 | 0.083897 |

| | | | |
|---|---|---|---|
| H | 0.014690 | 7.163829 | -0.443764 |
| H | 1.007445 | 6.093549 | -1.456680 |
| H | 1.213821 | 6.089423 | 0.291670 |
| H | -8.420525 | -0.320641 | 0.988251 |
| H | -3.712818 | 4.516693 | 0.843225 |
| H | -1.004309 | 1.757021 | -1.053100 |
